# Supplementary material for: HBM4EU Occupational Biomonitoring Study on e-Waste—Study Protocol
Source: Int J Environ Res Public Health. 2021 Dec 9;18(24):12987. doi: 10.3390/ijerph182412987 (PMC8701897; doi:10.3390/ijerph182412987)
Supplement: Supplementary file 1 [file ijerph-18-12987-s001.zip › Supplementary materials S1-S5.pdf]

Study Protocol

HBM4EU Occupational Biomonitoring Study on e-Waste—Study Protocol

Paul T. J. Scheepers [1,*], Radu Corneliu Duca [2,3], Karen S. Galea [4], Lode Godderis [3,5], Emilie Hardy [2], Lisbeth E. Knudsen [6], Elizabeth Leese [7], Henriqueta Louro [8,9], Selma Mahiout [10], Sophie Ndaw [11], Katrien Poels [3], Simo P. Porras [10], Maria J. Silva [8,9], Ana Maria Tavares [8], Jelle Verdonck [3], Susana Viegas [12,13], Tiina Santonen [10] and HBM4EU e-Waste Study Team †

1 Radboud Institute for Health Sciences, Radboudumc, P.O. Box 9101, 6500 HB Nijmegen, The Netherlands
2 Department of Health Protection, Laboratoire National de Santé (LNS), 1 rue Louis Rech, 3555 Luxembourg, Luxembourg; radu.duca@lns.etat.lu (R.C.D.); emilie.hardy@lns.etat.lu (E.H.)
3 Centre for Environment and Health, Department of Public Health and Primary Care, KU Leuven (University of Leuven), Kapucijnenvoer 35, 3000 Leuven, Belgium; lode.godderis@kuleuven.be (L.G.); katrien.poels@kuleuven.be (K.P.); jelle.verdonck@kuleuven.be (J.V.)
4 Institute of Occupational Medicine (IOM), Edinburgh EH14 4AP, UK; karen.Galea@iom-world.org
5 IDEWE, External Service for Prevention and Protection at Work, Interleuvenlaan 58, 3001 Heverlee, Belgium
6 Department of Public Health, University of Copenhagen, Øster Farimagsgade 5, 1353 Copenhagen, Denmark; liek@sund.ku.dk
7 Health & Safety Executive, Buxton SK17 9JN, UK; liz.leese@hse.gov.uk
8 INSA, National Institute of Health Dr. Ricardo Jorge, Department of Human Genetics, Av. Padre Cruz, 1600-609 Lisbon, Portugal; henriqueta.louro@insa.min-saude.pt (H.L.); m.joao.silva@insa.min-saude.pt (M.J.S.); a.tavares@insa.min-saude.pt (A.M.T.)
9 Centre for Toxicogenomics and Human Health, NOVA Medical School, Universidade NOVA de Lisboa, Campo dos Mártires da Pátria 130, 1169-056 Lisbon, Portugal
10 Finnish Institute of Occupational Health, Topeliuksenkatu 41 b, 00250 Helsinki, Finland; selma.mahiout@ttl.fi (S.M.); simo.porras@ttl.fi (S.P.P.); tiina.santonen@ttl.fi (T.S.)
11 INRS, French National Research and Safety Institute, 54500 Vandœuvre-lès-Nancy, France; sophie.ndaw@inrs.fr
12 NOVA National School of Public Health, Public Health Research Centre, Universidade NOVA de Lisboa, 1600–560 Lisbon, Portugal; susana.viegas@ensp.unl.pt
13 Comprehensive Health Research Center (CHRC), 1600–560 Lisbon, Portugal
* Correspondence: paul.scheepers@radboudumc.nl
† HBM4EU e-waste study members are listed in Acknowledgments.

Table of Contents

Supplementary material S1 Company information 4

Supplementary material S2 Participant information 8

Supplementary material S3 Informed consent 11

Supplementary material S4 - Questionnaires 14

Supplementary material S4.1 – Questionnaire 1 14

Supplementary material S4.2 – Questionnaire-2

Supplementary material S4.3 – Questionnaire-3

Supplementary material S5.1. SOP-1 Selection of participants and recruitment, information to the participants, informed consent 36

Supplementary material S5.2. SOP-2 Completion of company and worker questionnaires 43

Supplementary material S5.3. SOP-3 Blood sampling, including sample storage and transfer 56

Supplementary material S5.4. SOP-4 Urine sampling, including sample storage and transfer 76

Supplementary material S5.5. SOP-5 Settled dust 86

Supplementary material S5.6. SOP-6 Air sampling of inhalable and respirable dust fraction 95

Supplementary material S5.7. SOP-7 Obtaining hair samples 108

Supplementary material S5.8. SOP-8 Dermal sampling using wipes and wristbands 119

Supplementary material S5.9. SOP-9 Buccal cells sampling including sample storage and transfer 132

Supplementary material S5.10. SOP-10 Comparing occupational hygiene measurements with exposure estimates generated using the Advanced REACH Tool via the TREXMO model 143

Supplementary material S5.11. SOP-11 Communication plan for the occupational studies 154

Supplementary material S1 – Company information

HBM4EU occupational biomonitoring study on electronic -waste management

Information for Participating Companies

Introduction

We would like to invite your company to take part in HBM4EU (Human Biomonitoring for Europe), a pan-European study which aims to investigate workers' exposure to hazardous chemicals in the workplace.

HBM4EU is a European project running from 2017 to 2021 and including experts from 30 European countries and European Union agencies. It is funded by the European Commission's Horizon 2020 Program and by our national governments. Further details can be found at https://www.hbm4eu.eu/about-hbm4eu/.

We are reaching out to companies involved in electronic waste management i.e. involved in sorting, dismantling, shredding and pre-processing of electronic waste or are involved in further purification of electronic waste materials (metals or plastics) for re-use and re-sell. Your participation can further benefit your company's risk assessment & management and corporate responsibility practices, and ensure that you are complying with any upcoming regulatory measures concerning the occupational exposure to diisocyanates. Our role is completely funded and participation into the project does not result in any out-of-pocket costs for your company.

About the study

The aim of this study is to identify whether there is occupational exposure to hazardous chemicals during the e-waste management process. We will study the exposure to various metals, flame retardants, polychlorinated biphenyls and phthalates during the different phases of electronic waste management from the occupational hygiene and biological samples collected from the workers. The study will help to identify potential needs for improvements of occupational hygiene practice and to ensure the sustainable processing of e-waste.

From each participating company, we will need up to a total of 30 workers, as follows:

(a) up to 20 workers whose duties involve welding or surface treatment activities (however, if the company has e.g. multiple sites or multiple processes falling under the scope of the study, also higher number of participating workers is possible), and

(b) up to 10 workers who do not have such duties, to be used as a comparison group.

What does my company have to do if we agree to take part?

Once your company agrees to participate, the next steps are:

1. Following your signed consent, our research team will contact you to arrange a suitable time for our team members to visit your premises and speak to you and your workers about our study. An information leaflet will be given to your workers. Workers will then have to agree to participate by signing their own consent forms.
2. Our research team will visit your company on an agreed date to take biological and hygienic samples from participating workers and to ask them a few questions about their background and working routines, and to collect information on the relevant processes and work activities.
3. If we have your permission, we may take photographs of relevant processes and work activities, which will be amended such that workers or the company cannot be identified. This material may be also used for demonstration purposes in the information session in which results of the study are presented in your company.
4. Samples and information will be taken from your workers during working hours. For each worker, it will take 10-15 minutes to fill-in the questionnaire. Time will be needed also for blood samplings and urine samples.
5. Once samples are gathered, information regarding your company and workers' names will be coded and anonymity will be guaranteed.
6. Samples and information gathered from your workers will then go for analysis.

What are the benefits for the companies participating to the study?

This Pan-European study will help you to comply with your possible obligations under EU chemical legislation (REACH) and/or your obligations under the EU Directive on carcinogens or mutagens at work (Directive 2004/37/EC) and EU Chemical Agents Directive (Directive 98/24/EC).

By participating, your company will:

- Receive an electronic copy of the report with the collective results of the study published on the website of the HBM4EU project. The report will not contain any information identifying your company or your workers.
- Receive your company-specific hygienic report electronically. The report will include recommendations for the surveillance and monitoring procedures of your workers.
- You can also choose to receive an electronic report of the collective biomonitoring results of your workers, but will not receive individual results. Your workers will receive their personal results on exposure biomarker measurements.
- Your workers will gain awareness regarding the safety measures that they need to follow when carrying out activities in e-waste processing.

Does our company have to take part?

No, it is entirely up to you to decide whether or not to take part. If you do decide to take part, you will be asked to sign a consent form.

However, even if you sign the consent form you are still free to withdraw at any time and without giving reason and we will not approach any member of your staff to participate in the study. However, we will retain right to use any samples collected prior to the withdrawal in a confidential manner.

Are there any risks if my company joins HBM4EU?
There are no risks involved if your company joins HBM4EU, and your company's name will not be disclosed no matter what the results regarding chemical exposure.

How will my company's privacy be guaranteed?

- Participants are anonymous as their names are replaced with a code, and therefore cannot be traced as working for your company.
- Any information that may lead to the identification of a worker or your company will not be stated in any published reports i.e. country or company name.
- Computer security will be used to block any unauthorised access and all paper records will be securely stored.
- Information or samples provided to researchers will not include your company's registration numbers.
- Any results reported by this study will not contain information that may identify your company or staff. Only anonymous results of research conducted by the research team will be published and made available on the study's website: https://www.hbm4eu.eu/
- Our Data Protection Officer is at your disposal for questions or concerns related to data protection and may be reached at the following contact information: *[specify* (name and contact information of the Data Protection Officer of the legal entity)*]*.

Are there any costs involved?
There are no costs involved except brief working time of your staff members allocated for this study.

Who has reviewed the study?
The study is reviewed by the Bioethics committees and the Data protection commissioners of [Country]

Who do I contact if I would like more information about the study?

You can always reach out to [Name Surname of Research Coordinator of the Country]
Tel: [xxxxxxx] **Email:** [xxxxxxxx]

Supplementary material S2

Participant information

HBM4EU Occupational biomonitoring study on electronic waste management

Information for Participating Workers

You are invited to take part in a research study. Please read the following information to understand why the research is being done and what it involves. We are happy to answer any questions or concerns you may have.

About HBM4EU
People are exposed to a complex mixture of chemicals throughout their daily lives. These chemicals can be found in the environment, consumer products, food and drinking water and at workplaces.
Human biomonitoring involves collecting samples from people, e.g. blood, hair, saliva or urine, and measuring the levels of indicators of chemicals that are of interest. HBM4EU (Human Biomonitoring for Europe) is a European study, which aims to harmonize and use human biomonitoring to understand people's exposure to such chemicals and the related health risks, in order to improve chemical risk management. At workplaces, human biomonitoring can inform us on the need to reduce exposure. HBM4EU is funded by the European Commission and national governments. It includes experts from 30 countries and European Union agencies and will run from 2017 to 2021.
Learn more at https://www.hbm4eu.eu/.

About this study
We want to check if the current safety and control measures used at the workplaces across Europe can protect from the exposure to harmful substances in the recycling of electronic waste (e-waste) (such as metals, flame retardants, polychlorinated biphenyls and phthalates). In addition, we want to study new methods to assess the exposure to these chemicals.
The study has been approved by the [*national Bioethics Committee*] and complies with The General Data Protection Regulation (EU) 2016/679 (GDPR).

Why did you choose me?
We are inviting workers whose duties involve possible exposure to hazardous chemicals in the recycling of e-waste and workers without such duties, for comparison. You were chosen because

you work in recycling of e-waste, or we consider you as a suitable control person since your work tasks do not include such exposures. Your employer has consented to participate in the study and has agreed to you participating if you decide to do so.

What do I have to do if I agree to take part?
You don't have to change your usual routine since the study will take place during normal working hours. If you decide to participate, you will confirm your agreement by completing and signing the enclosed consent form. We will then find a suitable time to perform the study. You will meet the researchers during the work week for the collection of samples.

You will be asked to provide:

- Urine samples before the work shift at the beginning of your working week and after the work shift at the end of your working week. We will provide you with a collection bottle which you can give to the researcher.
- Two small samples of blood will be collected from the vein in your arm. These will be collected post-shift preferably in the end of the work week or at least after one or two days of work.
- A small sample of hair collected before the work shift at the beginning of your working week. We will provide instructions for the collection of hair samples.
- You will also need to fill a questionnaire which includes questions on your work tasks, personal protection and lifestyle aspects which may affect the results of the chemical analyses.

From some (not all) participants, we may also wish to collect personal air samples, which are collected by wearing a specific air sampling device on your work clothes, while you carry out your normal work tasks during one single work day. Wipe and wrist-band samples from your hands might also be collected during the work day in order to measure hand contamination.
Optionally and only if you consent to, we may take photographs to document your work area and practices. We will blur these materials to protect your identity and that of your company. You can still participate in the study without being present in photographs or video recordings.

What will happen to my samples, data and results?
We will replace your name with a code to protect your privacy and will transfer the samples to specialized laboratories for analysis.

Your samples will be examined to measure your exposure to metals, flame retardants, polychlorinated biphenyls and phthalates. Urine and blood samples might be additionally analyzed for the early, reversible cellular effects caused by the occupational chemical exposures in order to study the utility of these tests in the health surveillance of workers.

Your samples will not be analyzed for alcohol, prescription or illegal drugs.

Anonymized data collected from you and other participants will be stored and used for research purposes and may be combined with other data from different sources. Your anonymized samples will be stored at [specify place and length of storage] for use in future ethically approved biomonitoring studies to study chemical exposure. The researcher has the right to transfer your sample(s) and/or personal data, in coded form to protect my identity, to specialized laboratories to analyze the chemicals and markers specified in this leaflet.

Your *personal* results for measured metals and other chemicals will be reported to you [in approximately XX weeks/months by NNNN], unless you tell us you do not wish to receive them. When the results of the whole study will be finished, you will receive the summary of the collective results from all participating companies.

Your employer will receive the collective results of all the workers, but will not receive individual results. The *collective* results from all the participating companies will be published as a study report which will be openly accessed at https://www.hbm4eu.eu/

How will my privacy be guaranteed?
HBM4EU complies with the European Data Protection Regulation. We guarantee your anonymity by replacing your name with a code and protecting all electronic and paper records from unauthorized access. Published reports of the study will not contain information that can trace back to you or your company. Your employer or other third parties will not have access to your personal results, unless you consent to. Our Data Protection Officer is at your disposal for questions or concerns related to data protection and may be reached at the following contact information: *[specify* (name and contact information of the Data Protection Officer of the legal entity)*]*.

Why do you need my written consent?
Your written consent confirms that you volunteer to take part in the study, understand what is required from you and why. You will also confirm that we can contact you in the future to tell you about your personal results or for scientific, statistical or historical purposes.

How will I benefit if I participate?
The study will help to develop the safe working practices at your workplace. You will learn about your personal exposure to metals, flame retardants, polychlorinated biphenyls and phthalates during e-waste activities and obtain guidance how to reduce it.

Are there any risks if I join the HBM4EU study?
All sampling will be conducted by qualified and specially trained health professionals. There are no risks, other than possible minor discomfort during sample collection.

Can I quit the study?
We encourage you to discuss with us any concerns you may have, but you are free to withdraw the study at any time without consequences. We will, however, retain right to use any samples collected prior to the withdrawal in a confidential manner.

Are there any costs to me?

There are no costs to you. Your participation in the study will take place during your normal working hours (with the exception of the collection of urine samples first thing in the morning). You will not face salary deductions for your time commitment to the study.

Who do I contact if I'm unsure about anything or would like further information about the study?

Please contact [Name, Institute of National study Coordinator]

Tel: [xxxxxxx] **Email** [xxxxxxx]

Supplementary material S3 – Informed consent form

WORKER CERTIFICATE OF INFORMED CONSENT

Study description	
Title	HBM4EU Occupational biomonitoring study on electronic -waste management

Participant identifier	
Name	Tele-phone
Company	Email

Participant Code		Initials
1	I have read the companion "Information for Participating Workers" leaflet. I have had the opportunity to consider the information, ask any questions I wished to and received satisfactory answers.	
2	I understand that my participation is voluntary and that I am free to withdraw at any time without giving any reason, and without my medical care or legal rights being affected. The research team will, however, retain right to use any samples collected prior to the withdrawal in a confidential manner.	
3	The [*Specify organization*] will be able to contact me during working hours to collect my personal samples and information after I have consented to participate in this study.	
4	I consent that the [*Specify organization*] has the right transfer my sample(s) and/or personal data, in coded form to protect my identity, to specialized laboratories to analyze the chemicals and markers specified in the "Information for Participating Workers" leaflet. I understand that the Data Protection Officer of the *[specify* (name of the legal entity)*]* is at my disposal for questions or concerns related to data protection and may be reached at the following contact information: *[specify* (name and contact information of the Data Protection Officer of the legal entity)*]*.	
5	I consent that the [*Specify organization*] has the right to store my sample(s) and personal data in a biobank for [*no of years*] for future ethically approved biomonitoring studies.	
6	I understand that the [*Specify organization*] may contact me in the future regarding the use of my stored sample(s) and personal data. My contact details may be stored exclusively for this purpose and will not be disclosed to any third party.	
7	I consent to the use of cameras by the [*Specify organization*] as follows:<br>☐ I **do not** wish to be photographed during the research visit	

	☐ I give permission for photography, under the condition that the photographs will be blurred to protect my identity in any published reports and presentations.	
8	I understand that I will not benefit financially by taking part in this study.	
9	I understand that I will receive information on my personal results concerning exposure to metals, flame retardants, polychlorinated biphenyls and phthalates [*Specify substances*] I will receive this information from [*Specify for each country]*.<br>☐ I **do not** wish to be informed about the outcome of the study<br>In addition, when the whole study is completed, I will receive the summary of the final collective results of the study. I want to receive this by email/by post to the following address:<br>____	

....

Name of Participant Date Signature of Participant

Statement by the researcher/person taking consent

I confirm that the participant was given an opportunity to ask questions about the study, and all the questions asked by the participant have been answered correctly and to the best of my ability. I confirm that the individual has not been coerced into giving consent, and the consent has been given freely and voluntarily.

....

Name of Researcher / person taking the consent Date Signature of Researcher / person taking the consent

Supplementary material S4 - Questionnaires

Supplementary material S4.1 – Questionnaire 1

QUESTIONNAIRE FOR WORKPLACES (self-administered by company representative)

We would be grateful if you can complete this short questionnaire concerning your companies' activities relating to the **handling and recycling of e-waste (electrical and/or electronic equipment) only.** Whilst we understand that your company may be involved in the handling / recycling of other waste materials, these are not within the scope of our current project.

Please return it directly to the researcher once completed.

1. Company and Occupational Health care information

Name of the Company/Organisation:...

ID number of the company: (to be filled by the researcher):………………………………………………………………………

NACE Rev.2 code (to be filled by researcher):…………………………………………………………………………………………

Name and position of the company representative:...

Name of the Contractor/employment agency if workers are not directly employed:………………………………..

Name of the Contractor/employment agency representative:……………………………………………………………….

Name of the occupational health care provider (person/company)?...

Do all workers at the site have access to these services? If not specify:……………………………………………………….

Name of the department where the work is carried out:...

Site address:...

Country: ....

Industrial sector:..

Description of the workplace (nature of the business and what is the waste/raw material being handled and recycled):

....

....

1.1 Specifically, what kind of electric and electronic devices are received for processing? (Please describe)

....

....

....

1.2 Appropriately, what are the quantities of e-waste which are processed?

Annual quantities processed: ___________ tonnes

Highest processing month ___________

Lowest processing month____________

1.3 What kind of substances and components are extracted from e-waste in your company?

Via Manual methods (e.g. waste is moved or separated manually):

....

Via Mechanical (e.g. available equipment to move and separate the waste):

....

1.4 Could you describe what kind of information and/or training were given to the workers about the health risks in your company related to hazardous chemicals from e-waste processing activities?

....

....

2. Operational conditions

Please identify the various e-waste activities which are undertaken at your site, how frequently these take place and the numbers of workers typically involved. Please also indicate in general terms the types of risk management measures which are used.

**Job**	**Takes place at your site?** (tick if apply)	**Frequency of operations - daily, weekly, monthly, other, don't know)**	**Quantities processed per week (tonnes or kgs)**	**No. workers involved per day**	**Enclosed process? (yes / no)**	**Local exhaust ventilation in place? (Yes/ No)**	**RPE* worn ? (Yes / no)**	**Other PPE** worn ? (Yes / no)**
1. Collecting of e-waste	☐							
2. Transporting of e-waste	☐							
3. Sorting of e-waste	☐							
4. Repairing of electrical / electronic devices	☐							
5. Manual dismantling of e-waste	☐							
6. Cutting / shredding of e-waste	☐							
7. Compacting of e-waste	☐							
8. Burning / incinerating of e-waste	☐							
9. Removing wires	☐							
10. Burning wires	☐							
11. Smelting and recycling batteries	☐							
12. Producing secondary raw material (polymer processing)	☐							
13. Producing secondary raw material (metal melting)	☐							
14. Storage or simple handling of these wastes	☐							
15. Other tasks? Please, describe ____________________ ____________________ __________________	☐							

*RPE respiratory protective equipment; **PPE – personal protective equipment

3. Previous measurements

3.1 Did you collect environmental samples (e.g. air, dust) for metals or other chemical at work-place?

(Please circle) Yes No Don´t know

3.2 Were workers asked to carry equipment for personal air sampling for metals or other chemical substances?

(Please circle) Yes No Don´t know

If yes, have any of the following types of measurements been collected from your workers at the site?

Measurements	Tick all that apply	Years collected
Environmental or personal air samples	☐	
Dermal exposure measurements	☐	
Blood samples from workers	☐	
Urine samples from workers	☐	
Other (please specify)	☐	
	☐	

3.3 Would you be willing to allow the researchers to have access to the results of these measurements (in a confidential manner)?

(Please circle) Yes No

If yes, contact person and contact details (e-mail, phone number):

Name: ....

Phone number: ....

Email: ....

3.4 Do you have company's own occupational health care or are these services provided by an external service provider?

(Please circle) Own External

3.5 Which workers have access to occupational health provider(s)?

……………………………………………………………………………………

……………………………………………………………………………………………

…………………………………………………………………………………………….

3.6 Contact person and details of the Occupational Health and Safety department (if available):

Name: ……………………………………………………………

Phone number: ……………………………………………………………

Email: ……………………………………………………………

4. Hygiene facilities and procedures

**Hygiene facilities in the company** (please tick box if apply)	☐ Possibility to wash hands (hot and cold water)<br>☐ Possibility to take shower (hot and cold water)<br>☐ Separate lockers for working clothes<br>☐ Specific place for the storage of respiratory protective equipment<br>☐ Other (please specify)……………………………………<br>☐ ………………………………………………………………
**What is the procedure for washing work clothes?** (please, tick corresponding)	☐ Washing is organized by employer<br>☐ Work clothes are washed by workers at homes<br>☐ Work clothes are not washed as disposable items are worn<br>☐ Work clothes are not washed for other reasons
**How often are working clothes washed, if applicable?** (please, tick corresponding)	☐ At least once a week<br>☐ At least once in two weeks<br>☐ At least once a month<br>☐ Less than once a month, non-systematically
**What kind of cleaning methods are used in the facility?** (please, tick corresponding)	☐ HEPA-filtered vacuums<br>☐ Wet mops<br>☐ Sweeping with a broom<br>☐ Other (please, specify …………………………………………………)
**Is there any system for maintenance of ventilation**? (please, tick corresponding)	☐ Ventilation is cleaned on regular basis (please, specify how often ………………………………………...)<br>☐ Filters are changed on regular basis (please, specify how often ……………………………………….)<br>☐ Cleaning is formally recorded and retained<br>☐ Other (please, specify …………………………………………………)

Thank you for filling the questionnaire!

Please return it directly to the researcher once completed.

Supplementary material S4.2 – Questionnaire-2

POST-SHIFT QUESTIONNAIRE FOR WORKERS/CONTROLS (interviewed by researcher)

Background information about worker/control

**Urine sample**	**Date collected:**<br>**Sample code:**	**Time:**
**Blood sample**	**Date collected:**<br>**Sample code:**	**Time:**
**Hair sample**	**Date collected:**<br>**Sample code:**	**Time:**
**Air sample (personal)**	**Date collected:**	**Sample code:**
**Wipe sample(s) (personal)**	**Date collected:**	**Sample code(s):**
**Wrist-band (personal)**	**Date collected:**	**Sample code(s):**
**Settled dust (environmental)**	**Date collected:**<br>**Time:**	**Location:**<br>**Sample code:**
**Company name and name of department**		

**Worker name**	
**Worker position**	
**Sex** (please circle)	**Male** **Female**
**Date of birth (dd/mm/yyyy)**	

Interviewer: Name, position and contact details (e-mail, phone number)

Please separate this sheet (pages 3 and 4) from the Questionnaire

----

Supplementary material S4.3 – Questionnaire-3

QUESTIONNAIRE - FOR WORKERS/CONTROLS (continues; interviewed by researcher)

**What is your length (cm or feet/inches)**	……………. cm / ………… ft ………… inches	
**What is your current weight (kg or stones/lb)**	……………. kg / ………… St ………… lb	
**Occupation**	Free description:	ISCO08 code
**Is the work done predominantly** (please circle)	Inside Outside	
**Duration of work shifts (hours) and number and duration of breaks**		
**Typical overtime per week (hours)**		
**How many days have you worked after the last time you had a "day off"?**		
**Type of work shifts** (please tick box)	☐ Fixed day<br>☐ Fixed night<br>☐ Rotating day/back<br>☐ Rotating day/back/night<br>☐ Other (please specify)……………………………………………………	
**Home address** (for the delivery of results in case they cannot to be delivered to work address)		

**Home location and related characteristics** (please circle)	Urban Rural
**Are there industrial plants, incinerators or landfill sites in the surroundings of your house?** (please circle)	Yes No<br>If yes, approximately how far away from your house is the closest one (km)?
**Please describe the vehicular traffic density in the surroundings of your home address** (please circle)	☐ Pedestrian road (very low density)<br>☐ Quiet street (low density)<br>☐ Residential road (medium density)<br>☐ Main Road (heavy density)<br>☐ Highway (very heavy density)
**Cigarette smoking** (please circle)	Yes No Former smoker
**Cigarette smoking** (continues)	Approximate number of cigarettes/day ____________<br>Number of years you have smoked ________________<br>If former smoker, how many years ago did you stop smoking?_______<br>Approximate number of cigarettes/day you smoked ______________<br>Number of years you smoked ___________
**Do you smoke electronic cigarettes?** (please circle)	Yes No Former e-cigarette

**E-cigarettes** (continues)	How many times you usually recharge the tank of your e-cigarette in a day? __________<br>Please specify how many milliliters corresponds to each refill:<br>____________<br>Number of years you have smoked e-cigarettes ____________<br>If former e-cigarettes smoker, how many years ago did you stop smoking? _____________<br>How many milliliters you approximately use in a day?____________<br>Number of years you smoked e-cigarettes ___________
**Do you use any other tobacco products?** (please circle)	Yes No Former user<br>If yes or former user, please specify:____
**Other tobacco products** (continues)	Approximate number of tobacco product/day _________________<br>Number of years you have used __________________________<br>If former user, how many years ago did you stop?________________<br>Approximate number of product/day you used __________________<br>Number of years used ___________________
**During the past 3 months, have you had a medical x-ray or Computerised Axial Tomography (CAT) scan?** (please circle)	Yes No
**Have you previously, or are you currently, being treated for cancer?** (please circle)	Yes No
**Alcohol consumption**	Yes No<br>How often do you typicially drink alcohol? (please, circle)<br>Daily weekly monthly<br>Please specify the type of alcoholic beverage (e.g. wine, beer, whiskey, etc)

**Alcohol consumption** (continues)	On average, how many days in a month do you have at least one alcoholic beverage?<br><br>On a typical day that you drink alcohol, how many drinks do you usually have?
**Consumption of other beverages** (please circle)	Coffee Tea Energy drinks<br>On average, how many times in a typical day?<br>Coffee ………. Tea ………. Energy drinks ……….
**Dietary habits** (please circle)	Mixed Vegetarian Vegan<br>Other (please specify)
**Consumption of seafood and fish**	How often do you eat fish and / or sea food?<br>□ Never<br>□ 1-2 days per week<br>□ 3-4 days per week<br>□ Every day
**Do you wash your hands after or during the work shifts?** (please, tick corre-sponding)	After work<br>□ Always<br>□ Sometimes<br>□ Never
	Before eating, drinking<br>□ Always<br>□ Sometimes<br>□ Never
	Before smoking<br>□ Always<br>□ Sometimes<br>□ Never
	Before using restroom<br>□ Always<br>□ Sometimes<br>□ Never

**Use of food supplements**<br><br>(e.g. diet pills) (please circle)	Yes No<br><br>If yes, please specify:
**Recreational activities, secondary occupations or hobbies which may cause may cause additional metal/other relevant exposure (e.g. welding, paint spraying, metal works, electronic devices repair, firefighting, hobbies involving shooting)**	Yes No<br><br>If yes, please specify:<br><br>____<br><br>____<br><br>Duration:<br><br>____<br><br>____
**Do you have implants which may contain metals? (please circle)**	Yes No Don't know<br><br>If yes, how long?__________<br><br>Do you know what type of implants? (please specify)<br><br>____
**Do you have dental fillings? (please circle)**	Yes No<br><br>If yes, do you know what material they are made? (please specify)<br><br>____

COVID-19 Symptoms and diagnosis

**Have you had one or more of the following symptoms during the past 14 days?** (please tick box if apply)	☐ **Cough**<br>☐ **Sore throat**<br>☐ **Headache**<br>☐ **Muscle ache/pain**<br>☐ **Fever**<br>☐ **Dyspnoea (difficult breathing)**<br>☐ **Reduced or loss of senses of taste and smell**<br>☐ **Nausea or vomiting**

	☐ **Diarrhoea**<br>☐ **Chest pains**<br>☐ **Skin rashes**
**Have you been diagnosed with coronavirus disease (COVID-19) based on a test by a doctor/general practitioner/other?** (please circle)	**Yes** **No**<br><br>**If yes, when?**
**Have you ever been hospitalised due to COVID-19?** (please circle)	**Yes** **No**<br><br>**If yes, when?**
**Have you been vaccinated past week?** (please circle)	**Yes** **No**<br><br>**If yes, against what?**
**Have you been vaccinated against severe acute respiratory syndrome coronavirus 2 (SARS-CoV-2)?** (please circle)	**Yes** **No**<br><br>**If yes, please give the date** (dd/mm/yyyy)**?**

Occupational history including the present occupation

For the needs of the study we would like to know your previous workplaces in this company and in others companies where you have worked before.

Please fulfill the following table:

Job description (describe also changes in the workplace in the actual company)	Starting date (MM/YY)	Finishing date (MM/YY)

Q3 Questionnaire JOB DESCRIPTIONS (interviewed by researcher during the sampling)

Job description: E-waste recycling (please list the type of work tasks you have been involved in today)

	Work task	**Duration of the task in a work shift** (hours/ minutes)	**Frequency of the task** (x times per week)	**Process type** (manual or automatic)	**RPE* used** (add the corresponding numbers)*	**PPE** used** (add the corresponding numbers)**	**LEV used***** (yes / no) (add the corresponding numbers)***
1	**Collecting of e-waste**						
2	**Transporting of e-waste**						
3	**Sorting of e-waste**						
4	**Repairing of electrical / electronic devices**						
5	**Manual dismantling of e-waste**						
6	**Cutting / shredding of e-waste**						
7	**Compacting of e-waste**						
8	**Burning / incinerating of e-waste**						
9	**Removing wires**						
10	**Burning wires**						
11	**Smelting and recycling batteries**						

	Work task	**Duration of the task in a work shift** (hours/ minutes)	**Frequency of the task** (x times per week)	**Process type** (manual or automatic)	**RPE* used** (add the corresponding numbers)*	**PPE** used** (add the corresponding numbers)**	**LEV used***** (yes / no) (add the corresponding numbers)***
**12**	**Producing secondary raw material (polymer processing)**						
**13**	**Producing secondary raw material (metal melting)**						
**14**	**Landfilling**						
**15**	**Cleaning and maintenance of equipment**						
**16**	**Maintenance**						
**17**	**Cleaning of production facilities/buildings**						
**18**	**Other** (please specify)						

*RPE (respiratory protective equipment) worn:

1. Reusable particulate respirator (please specify how often the filters are changed)
2. Disposable particulate respirator
3. Other respiratory protection equipment (please specify: e.g. vapors protection besides particles)

**PPE (Personal protective equipment) worn:

1. Re-usable working clothes (coveralls)
2. Disposable working clothes (protection suit)
3. Working shoes / boots
4. Arm guards

5. Cut resistant gloves
6. Reusable gloves
7. Disposable gloves
8. Safety glasses/goggles
9. Face protection
10. Other (please specify)

*** Where the Local Exhaust ventilation is located:
1. in a certain space/ area
2. dedicated to a specific machine:

**If used, has your respiratory protection equipment (mask) been fit tested?** (please circle)	**Yes** **No**<br><br>**If yes, when?**
**Have you received information, instruction or training on the use of safe work practices when carrying out e-waste related work?** (please circle)	**Yes** **No**<br><br>**If yes, when?**
**What kind of general ventilation is available in the workplace?**	☐ **Natural (e.g. open doors / windows)**<br>☐ **Mechanical (e.g. fans, blowers)**<br>☐ **Both**<br>☐ **Do not know**
**Have the work conditions been normal during the work day?** (please circle)	**Yes** **No**<br><br>**If not normal, please specify (e.g. problems with mask or extraction not working):**

Job description: hydrometallurgical processing (please list the type of work tasks you have been involved in today)

	**Work task**	**Duration of the task in a work shift** (hours/ minutes)	**Fre-quency of the task** (x times per week)	**Process type** (man-ual or auto-matic)	**RPE* used** (add the cor-responding numbers)*	**PPE* used** (add the cor-responding numbers)*	**LEV used***** (yes / no) (add the cor-responding numbers)***
1	**Leaching**						
2	**Recovery of met-als from leachate - precipitation**						
3	**Recovery of met-als from leachate – solvent extrac-tion**						
4	**Recovery of met-als from leachate – adsorption of activated carbon**						
5	**Recovery of met-als from leachate – ion exchange**						
6	**Other** (please specify)						

*RPE (respiratory protective equipment) worn:

4. Reusable particulate respirator (please specify how often the filters are changed)
5. Disposable particulate respirator
6. Other respiratory protection equipment (please specify: e.g. vapors protection besides particles)

**PPE (Personal protective equipment) worn:

11. Re-usable working clothes (coveralls)
12. Disposable working clothes (protection suit)
13. Working shoes / boots
14. Arm guards
15. Cut resistant gloves
16. Reusable gloves
17. Disposable gloves
18. Safety glasses/goggles
19. Face protection

20. Other (please specify)

*** Where the Local Exhaust ventilation is located:

3. in a certain space/ area
4. dedicated to a specific machine:

**If used, has your respiratory protection equipment (mask) been fit tested?** (please circle)	**Yes** **No**<br><br>**If yes, when?**
**Have you received information, instruction or training on the use of safe work practices when carrying out e-waste related work?** (please circle)	**Yes** **No**<br><br>**If yes, when?**
**What kind of general ventilation is available in the workplace?**	☐ **Natural (e.g. open doors / windows)**<br>☐ **Mechanical (e.g. fans, blowers)**<br>☐ **Both**<br>☐ **Do not know**
**Have the work conditions been normal during the work day?** (please circle)	**Yes** **No**<br><br>**If not normal, please specify (e.g. problems with mask or extraction not working):**

Job description: biometallurgical processing (please list the type of work tasks you have been involved in today)

	**Work task**	**Duration of the task in a work shift** (hours/ minutes)	**Frequency of the task** (x times per week)	**Process type** (manual or automatic)	**RPE* used** (add the corre-sponding numbers)*	**PPE* used** (add the corre-sponding numbers)*	**LEV used***** (yes / no) (add the corre-sponding num-bers)***
1	**Bioleaching**						
2	**Biosorption**						
3	**Other** (please specify)						
4	**Other** (please specify						

*RPE (respiratory protective equipment) worn:

7. Reusable particulate respirator (please specify how often the filters are changed)
8. Disposable particulate respirator
9. Other respiratory protection equipment (please specify: e.g. vapors protection besides particles)

**PPE (Personal protective equipment) worn:

21. Re-usable working clothes (coveralls)
22. Disposable working clothes (protection suit)
23. Working shoes / boots
24. Arm guards
25. Cut resistant gloves
26. Reusable gloves
27. Disposable gloves
28. Safety glasses/goggles
29. Face protection
30. Other (please specify)

*** Where the Local Exhaust ventilation is located:

5. in a certain space/ area
6. dedicated to a specific machine:

**If used, has your respiratory protection equipment (mask) been fit tested?** (please circle)	**Yes** **No**<br><br>**If yes, when?**

**Have you received information, instruction or training on the use of safe work practices when carrying out e-waste related work?** (please circle)	**Yes** **No**<br><br>**If yes, when?**
**What kind of general ventilation is available in the workplace?**	☐ **Natural (e.g. open doors / windows)**<br>☐ **Mechanical (e.g. fans, blowers)**<br>☐ **Both**<br>☐ **Do not know**
**Have the work conditions been normal during the work day?** (please circle)	**Yes** **No**<br><br>**If not normal, please specify (e.g. problems with mask or extraction not working):**

Supplementary material S5.1

SOP-1 Selection of participants and recruitment, information to the participants, informed consent

Authors and Acknowledgements

Lead authors:

This document has been developed by Susana Viegas from Escola Superior de Tecnologia da Saúde de Lisboa (ESTeSL), Instituto Politécnico de Lisboa.

Contributions were received from: Karen Galea (IOM, UK), Sophie Ndaw (INRS, France), Paul Scheepers (Radboudumc, the Netherlands)

This document has been created for the HBM4EU project. HBM4EU has received funding from the European Union's Horizon 2020 research and innovation programme under grant agreement No 733032.

Summary

Most relevant aspects:

- ✓ Workers are from companies performing e-waste management processes.
- ✓ Workers from both genders and ages ranging from 18-70 years will be considered.
- ✓ Control subjects should match for age, gender and smoking status from companies in the same geographical area but working on activities not related with e-waste management processes.
- ✓ Additional criteria should be followed for workers and controls in the case of biomarkers of effect.
- ✓ SOP details the specific documents to be delivered to companies, workers and controls.

1 Introduction

Standard Operation Procedure 1 (SOP 1) is focussed on the selection of participants and recruitment, information to the participants and informed consent. This was developed with due consideration of the contents of the "SOP1: Standard operating procedure for selection of participants and recruitment, information to the participants, informed consent (SOP 1)" (lead authors Carina Ladeira, Edna Ribeiro, Susana Viegas, ESTeSL) developed for the HBM4EU occupational biomonitoring study on hexavalent chromium and other harmful chemicals (Porras et al., 2019).

Study design and participants

The study is focussed on companies performing e-waste management processes located in several countries from Europe, namely: Belgium, Finland, Germany, Hungary, Latvia, Luxembourg, The Netherlands, Poland, Portugal and United Kingdom.

The target population will be workers from companies performing e-waste management processes such as:

1) Sorting e-waste from household and industrial waste streams (by hand or semi-automatic)
2) Dismantling to split casings from electronic components such as circuit boards and batteries (often by hand)
3) Shredding and pre-processing (e.g. on a belt by electrostatic, density, magnetism, colour separation)
4) Further purification of e-waste materials for re-use and re-sell (melting metals to a product that can be re-used and polymer processing to a granulated product that can be re-used).

Target population

The target population will be workers involved in the above-described e-waste processes. In addition, a control population of workers will also be recruited. These workers will be from the same geographical region but working in offices/administrative tasks.

Both genders will be eligible, with ages ranging from 18-70 years. For some analyses, e.g., effect biomarkers characterization, specific inclusion/exclusion criteria are defined because those biomarkers are affected by several confounding factors that should be reduced to avoid results misinterpretation.

Sample size

The target population size for this project is 25 - 50 workers *per* country. The sample size is indicative and may need further justification due to expected population variability of the biomarkers. Additionally, 25 workers (controls) *per* country will be also engaged in the study. Overall, this would result in 500 exposed workers and 250 control workers.

Selection of sampling locations

Sampling will be conducted in previously identified companies located in the ten countries participating in the study, namely: Belgium, Finland, Germany, Hungary, Latvia, Luxembourg, The Netherlands, Poland, Portugal and United Kingdom. Companies will be contacted, informed about the study aims and invited to participate. The same approach will be followed for the workers' engagement in the study.

Selection of participants, their recruitment and information

Recruitment and information provision will be undertaken in the local language. For this purpose, a two-step approach will be undertaken, the first one for the company itself and the second one related to the companies' workers. Workers should be given sufficient time to reach a decision regarding their participation and be given the opportunity to ask any questions about their role and contribution to the research team.

Company recruitment

The company's responsible will be contacted by phone contact or e-mail. Upon company expressing interest in participating in the study, the information leaflet, "INFORMATION FOR PARTICIPATING COMPANIES" will be issued. Where country specific rule requires its use (e.g., Belgium) upon a company agreeing to participate in the study an authorised representative of the Company must complete the ***"EMPLOYER CERTIFICATE OF INFORMED CONSENT"***. Where country specific rules do not require it's use completion of this certificate is not necessary. Then, request to the company responsible of a list of names of those involved in e-waste management processes so that we can approach them to explain the project and invite them to participate.

Worker recruitment

In most countries recruitment cannot start before ethics approval has been obtained.

1. Establishment of a direct contact between the researcher and the worker, which is recommended to be done through a direct face-to-face meeting. Information on the study scope and actions to be developed (sample collection and filling in a questionnaire) will be provided to the workers. The information leaflet on the study *"INFORMATION FOR PARTICIPATING WORKERS"* will be distributed and discussed during the first contact with the workers. Within this contact, a reasonable period of time to clarify all workers queries regarding the project is mandatory. The workers should fill in the "WORKER CERTIFICATE OF INFORMED CONSENT" if they are willing to give their informed consent to participate in the study.

Therefore, and following workers' acceptance to participate in the study, we will recruit workers directly involved in the processes already described in Section 2.

In addition, we will recruit a group of unexposed (control) subjects, individually matched to the subjects for age (plus/minus 5-years), gender and smoking status (current smoker/ex-smoker/non-smokers). These control subjects will be selected from companies in the same geographical area but involved in activities not related with e-waste management processes. The same approach used for workers will be followed to recruit controls.

All the subjects should be in good health and present at work during the planned period of the study. Blood samples to be used in in genotoxicity biomarkers study should only be obtained from workers that, in addition to the above criteria, should fulfil the following inclusion criteria: i) are under the age of 50; ii) are non-smokers or ex-smokers for more than six months; iii) have not been subjected to a medical exam such as a medical X-ray or Computerised Axial Tomography (CAT) scan in the last 3-months; iv) have not been diagnosed for cancer.

Results communication

Results communication should follow SOP 9 (Communication Plan). Moreover, the definition of which results, to whom and how the HBM results will be communicated should be defined in the beginning of the study following the General Data Protection Regulation requirements.

Informed Consent form

Prior to the workers contact, and where country specific rule requires its use (e.g., Belgium), the company should analyse and sign the Informed Consent Form ***("EMPLOYER CERTIFICATE OF INFORMED CONSENT")***. The workers and controls accepting to participate in the study must sign the consent form ("WORKER CERTIFICATE OF INFORMED CONSENT") before the collection of any information or samples.

The Informed Consent form can be signed only after receiving information explaining the aims of the study and all details required by the appropriate ethical regulations in each country. The researcher must be available for clarification during the reading of the consent form by the participants (company representative, workers and controls). The workers should have the time and opportunity to ask questions to the researcher and not be under pressure to decide.

The Informed Consent forms should be co-signed by the researcher and be archived and kept during all the study duration in each institution that participates in the sample collection (not less than 5 years). Identification of companies and workers will not be used in all the process of samples handling and storage to guarantee the confidentiality needed.

Assignment of participant and sample codes

A standardised convention will be used to assign unique identification codes for all samples collected. The identification code convention is as follows:

E-waste (E) - Country ID (XX) - Company ID (XX) - Participant ID (XXX) - Sample ID (AX/BX/LCX/RCX/HX/SDX/UX/WX/WBX)

'E' is to denote that the samples and data relate to the e-waste occupational study.

Country ID 'XX' is the country code, using the ISO Alpha-2 country codes for the participating countries (http://www.nationsonline.org/oneworld/country_code_list.htm).

Country	ISO Alpha-2 country codes
Belgium	BE
Finland	FI
Germany	DE
Latvia	LV
Luxembourg	LU
Poland	PL
Portugal	PT
The Netherlands	NL
United Kingdom	UK

Company ID 'XX' is a two-digit running number of companies in each country (e.g. 01 for the first company recruited, 02 the second and so forth).

Participant ID 'XXX' is a three-digit running number of participants in each country (e.g. 001 for the first participant recruited, 002 the second and so forth).

Sample ID 'AX/BX/LCX/…' is one or two letters (A/B/LC/RC/H/SD/U/W/WB) to identify the type of sample collected, followed by an one-digit identifier (X) to identify the running number of each type of sample for that worker (e.g. 1 for the first sample, 2 for the second and so forth). The letter code applied for the sample types is as follows:

Type of sample collected	Sample type code
Air	A
Blood	B
Buccal cells	LC (left cheek) or RC (right cheek)
Hair	H
Settled dust	SD
Urine	U

Wipe	W
Wrist band	WB

The following scenario is provided to illustrate the application of this convention.

A worker is recruited in Portugal. He is working in the first company recruited. He is the first worker recruited in that company and is providing his first two wipe samples. The sample identification codes assigned are therefore:

E-PT-01-001-W1

E-PT-01-001-W2

References

Porras S, Ladeira C, Ribeiro E, Viegas S, Uuksulainen S, Santonen T, Galea K, Cherrie J, Louro H, Ventura C, Silva MJ, Leese E, Jones K, Hanser O, Ndaw S, Robert A, Duca R-C, Poels K, Godderis L, Kiilunen M, Norppa H, Veijalainen H, Parshintsev E, Tuomi T, Ruggieri F, Alimonti A, Koch H, Bousoumah R, Antoine G, Jacoby N, Musgrove D. (2019) HBM4EU occupational biomonitoring study on hexavalent chromium and other harmful chemicals. Standard Operating Procedures (SOPs). WP8 - Targeted field work surveys and alignment at EU level. Available from URL: https://www.hbm4eu.eu/online-library/ (last accessed 22nd February 2021).

Supplementary material S5.2

SOP-2 Completion of company and worker questionnaires

Authors and Acknowledgements

Lead authors:

This document has been developed by Susana Viegas (ESTeSL), Portugal

Contributions were received from: Liz Leese (HSE, UK), Karen Galea (IOM, UK), Sophie Ndaw (INRS, France), Lode Godderis (KU Leuven, Belgium)

This SOP was developed with due consideration of the contents of the "SOP2: Standard operating procedure for completion of company and worker questionnaires" (lead authors Sanni Uuksulainen (FIOH, Finland), Simo Porras (FIOH) and Karen Galea (IOM, UK)) developed for the HBM4EU occupational biomonitoring study on hexavalent chromium and other harmful chemicals (Porras et al., 2019).

This document has been created for the HBM4EU project. HBM4EU has received funding from the European Union's Horizon 2020 research and innovation programme under grant agreement No 733032.

Summary

Most relevant aspects

This SOP defines:

- ✓ Instructions to fill in the workplace questionnaire
- ✓ Instructions for completion of the workers post-shift and controls questionnaire
- ✓ Instructions to provide job descriptions on different activities

2 General introduction

This SOP 2 – "Completion of company and worker questionnaires" is designed to support a targeted occupational study on E-waste performed under task 8.5.

This SOP has been created with the premise in mind that every participating country is obliged to try, as far as is reasonably practicable, to follow the HBM4EU documents to achieve comparable data in a (as much as possible) harmonised and consistent manner.

As stated in SOP 1 (Selection of participants and recruitment, information to the participants, informed consent), the target population are workers from companies performing e-waste management processes located in several European countries. The exact processes used in the companies and by workers will be specified during the collection of contextual information, which is guided on this SOP.

Two questionnaires will be used to collect relevant contextual information for the study:

1. Self-completed company questionnaire
    - to be completed by the company representative, prior to the sampling campaign commencing
2. Interview led post-shift worker questionnaire
    - to be completed by the researcher while interviewing the worker, and
    - to be completed as close as possible to the end of work shift.

It should be noted that some questions (related with workplace contextual information) will not be applicable to the control group (those participants not working in E-waste management processes) and these questions will be clearly identified as such. Additionally, it´s important to clarify that the home address is questioned to guarantee that the participants receive their individual HBM results (as detailed in the participant information leaflet) report when available. Moreover, the questions related with alcohol and tobacco consumption, diet, specific diseases and medical examinations are needed because biomarkers of effect are going to be analysed in the samples of some countries. In this case, the countries that are not going to consider biomarkers of effect should not ask these questions.

It is important that information entered into the hard copy questionnaires is recorded as per the instructions given in this SOP as this information will need to be entered into the data template for the overall E-waste study.

3 Instructions to fill in the workplace questionnaire (self-administered by company representative)

This questionnaire is to be completed by the company representative before the sample collection campaign starts. The questionnaire should all be self-explanatory however, further information is provided below should the company representative require additional details. This explanatory information also acts as an aide memoir for the researcher.

Please ask the representative to fill in the questionnaire and return it directly to you (researcher) once completed.

Questionnaire explanatory text:

- **Company and occupational health care information –** This section is to be completed by all respondents
  - Information on sector and description of the workplace are needed to be able to present the study results in aggregated form. Sector of use and nature of the business can be described in free text.
  - The researcher will fill the NACE Rev.2 code. Copy corresponding NACE code (with 4 digits) and label text from the link. The classification is available in several EU-languages at the following URL:
    http://ec.europa.eu/eurostat/ramon/nomenclatures/index.cfm?TargetUrl=LST_NOM_DTL&StrNom=NACE_REV2&StrLanguageCode=EN&IntPcKey=&StrLayoutCode=HIERARCHIC

Most of the sectors of use in this study belong to the following classes: 38.12 Collection of hazardous waste; 38.22 Treatment and disposal of hazardous waste; 38.31 Dismantling of wrecks and 38.32 Recovery of sorted materials classes.

- **Operational conditions -** This section is to be completed by all respondents.
  - The work tasks performed in the company need to be ticked, which will then direct the company representative to the sections that they need to complete. Sections of the questionnaire relating to work tasks not relevant to the company are omitted.
  - The researcher should double check that the corresponding sections of the ticked work tasks are completed.

- **Section 1 - Previous measurements:** This section is to be completed only if the companies have undertaken previous environmental or biomonitoring campaigns to evaluate workers' exposure to chemical substances.
  - Background information about the previous exposure measurements is very important when estimating the risks of exposure. If data on previous measurements is available for the researchers, the exposure trends can be determined. The researcher should highlight the confidential nature of providing the previous measurement data.
  - If the company representative is unaware of the type of previous measurements that have been collected or the years in question, this should be recorded as free text on the questionnaire as 'Don't know'. A request should then be made for this information to be followed up and provided, where possible, by no later than the time of completion of the sampling campaign at the site.

- **Section 2 - Hygiene facilities and procedures:** This section is to be completed with the most detailed information, signalizing the boxes and providing additional information if available.

Upon return of the questionnaire, the researcher should ensure that all questions are completed and that handwriting is legible. In the event that it is difficult to read the handwriting, the researcher should ask for clarification and rewrite the response in their own writing. The researcher is also required to complete the NACE Rev.2. Finally, for questions where a range of options relate to an answer e.g. grams or liters; daily, days/week or days/month, the researcher should double check with the respondent which option their response relates to.

4 Instructions for completion of the workers post-shift and controls questionnaire (interviewed by researcher)

This is an interview-led questionnaire with the responses being entered by the researcher. The interview-led questionnaire is to be completed as close as possible to the end of work shift where possible. The researcher can ask the site if the workers can finish a little earlier to allow the completion of the questionnaire. If this is not possible, the questionnaire should be filled in the next possible moment with due consideration of worker and researcher availability. The administration of the questionnaire should take place (where possible) in a quiet area, free from distractions.

Questionnaire is divided into three parts

- Background information about worker
  - To be completed for both exposed workers and controls
- Job description and personal habits
  - To be completed for the exposed workers and controls
  - Personal habits (cigarette, alcohol consumption, diet) asked are relevant for data analysis.
- Occupational history
  - To be completed for both exposed workers and controls. Please ask about all jobs lasting more than 12 months since leaving school or full-time education.

The interviewer should pay attention especially to the following matters and it is recommended that capital letters are used to record free text answers to aid in reading at the time of data entry:

1. **Background information about workers (to be completed for both exposed workers and control group)**

- **Worker ID:**

A standardised convention will be used to assign unique identification codes for all workers and controls. The identification code convention is as follows:

E-waste (E) - Country ID (XX) - Company ID (XX) - Participant ID (XXX)

'E' is to denote that the samples and data relate to the e-waste occupational study.

- Country ID 'XX' is the country code, using the ISO Alpha-2 country codes for the participating countries (http://www.nationsonline.org/oneworld/country_code_list.htm).

Country	ISO Alpha-2 country codes
Belgium	BE

Finland	FI
Germany	DE
Latvia	LV
Luxembourg	LU
Poland	PL
Portugal	PT
The Netherlands	NL
United Kingdom	UK

Company ID 'XX' is a two-digit running number of companies in each country (e.g. 01 for the first company recruited, 02 the second and so forth).

Participant ID 'XXX' is a three-digit running number of participants in each country (e.g. 001 for the first participant recruited, 002 the second and so forth).

The following scenario is provided to illustrate the application of this convention.

A worker is recruited in Portugal. He is working in the first company recruited and he is the first worker recruited in that company. The worker identification code assigned is therefore: E-PT-01-001

- **Information related to the sample collection:** Fill in the information regarding the samples collected. Pay attention to record the actual sampling time for each sample. Note that not all samples may be collected. In which case "N/A" (not applicable) should be recorded in the relevant box.

A standardised convention will be used to assign unique identification codes for all samples collected. The identification code convention is as follows:

E-waste (E) - Country ID (XX) - Company ID (XX) - Participant ID (XXX) - Sample ID (AX/BX/LCX/RCX/HX/SDX/UX/WX/WBX)

'E' is to denote that the samples and data relate to the e-waste occupational study. Country ID 'XX', Company ID 'XX' and Participant ID 'XXX' are as above.

Sample ID 'AX/BX/LCX/…' is one or two letters (A/B/LC/RC/H/SD/U/W/WB) to identify the type of sample collected, followed by an one-digit identifier (X) to identify the running number of each type of sample for that worker (e.g. 1 for the first sample, 2 for the second and so forth). The letter code applied for the sample types is as follows:

**Type of sample collected**	**Sample type code**
Air	A
Blood	B
Buccal cells	LC (left cheek) or RC (right cheek)
Hair	H

Settled dust	SD
Urine	U
Wipe	W
Wrist band	WB

The following scenario is provided to illustrate the application of this convention.

A worker is recruited in Portugal. He is working in the first company recruited. He is the first worker recruited in that company and is providing his first two wipe samples. The sample identification codes assigned are therefore:

E-PT-01-001-W1

E-PT-01-001-W2

- **Company name and department:** Company name will already be known but name of department will need to be requested

- **Worker name**: Name of the worker is needed in order to be able contact the worker in the future to tell him/her about his/her personal results or regarding the use of his/her stored sample(s) and personal data in the future studies. The name will be replaced with a code to protect worker's privacy.

- **Sex:** The background exposure to chemicals may differ in men and women. Circle response. If an individual advises, they do not wish to respond to this question, it should be left blank.

- **Date of birth:** This question is essential to identify potential differences in human exposures, as well as susceptibility associated with the age. Record as dd/mm/yyyy.

The front page of the worker questionnaire, containing the personal information, should now be removed from the rest of the questionnaire. Before doing so please check that the remaining pages of the worker questionnaire have the relevant unique identification codes entered.

- **Height and current weight:** This information is used to calculate body mass index (BMI). Researcher to take care in ensuring correct units are assigned.

- **Free description of occupation:** Please describe as detailed as possible to help to choose the relevant ISCO-code. Copy corresponding ISCO-08 code (with 4 digits) and text label from the link. The classification is available in English, German and French at the following URL:
  http://ec.europa.eu/eurostat/ramon/nomenclatures/index.cfm?TargetUrl=LST_NOM_DTL&StrNom=CL_ISCO08&StrLanguageCode=EN&IntPcKey=&StrLayoutCode=HIERARCHIC

  - **Outside or Inside work:** Exposure may vary depending whether the work is done outside or inside. Air flows or wind conditions may lower the exposure. Circle response.

- **Duration of work shifts:** Please enter typical duration of a work shift with partial hours being recorded as follows: 30 mins is 0.5 hours; 15 mins is 0.25 hours. Therefore, a 7 and a half hour work shift would be recorded as 7.5 hours.

- **Type of work shifts:** Note 'back' shift typically refers to a shift starting in the afternoon and finishing in the evening, e.g. 14.00-22.00

- **Home address** is needed in order to be able contact the worker in the future to tell him/her about his/her personal results or regarding the use of his/her stored sample(s) and personal data in the future studies. Worker's contact details will be stored exclusively for this purpose and will not be disclosed to any third party.

- **Location and related characteristics**: These questions aim to characterize the environment where the participant lives, as differences could exist in human exposure associated with the area of residence. Urban areas are very developed, meaning there is a density of human structures such as houses, commercial buildings, roads, bridges, and railways. "Urban area" can refer to towns, cities, and suburbs. In general, a "Rural area" is a geographic area that is located outside towns and cities. In other words, whatever is not urban is considered rural. Circle response

- **Industrial plants, incinerators or landfill sites in the surroundings of house:** It is necessary to collect information on facilities considered as potential sources of exposure to pollutants, which might lead to differences in human exposure levels. Likewise, this question provides information on the general characteristics of the living environment (e.g. if the house is located in a heavily industrialized area there might be high background exposure). Circle response and in the event that a Yes response is given prompt for the distance in km. In the event that the respondent indicates that they do not know the answer to this question 'Don't know' should be recorded.

- **Vehicular traffic density:** Traffic density may have an impact on exposure to chromium, cadmium and lead. Circle response.

- **Smoking habits (including all tobacco and e-cigarette products)**: Information on smoking habits and passive exposure to tobacco smoke have to be collected since these are well known sources of exposure to a wide variety of substances such as Cr and Cd. The researcher should circle whether the respondent is a current smoker and if not, a former smoker. To assist the respondent in estimating the number of cigarettes smoked per day, a standard cigarette pack contains 20 cigarettes.

- **Metal containing implants**: This question is used to identify persons with artificial joints etc. in their body. Their data may need to be treated separately in some data analysis.

- **Dental fillings:** Amalgam fillings could be a source of exposure to metals.

- **Medical X-ray or Computerised Axial Tomography (CAT):** This question is asked because such treatment during the last 3-months may affect on the genotoxicity markers. If the workers responds 'yes' their blood sample number Tube 1 will not be analysed for these biomarkers and a note of this should be made on the corresponding blood sample information form (see blood sampling SOP 3).

- **Cancer:** If the worker has been treated for cancer, it may affect on the genotoxicity markers. If the workers responds 'yes' their blood sample number Tube 1 will not be analysed for these biomarkers and a note of this should be made on the corresponding blood sample information form (see blood sampling SOP 3).

- **Alcohol consumption:** Alcohol has been identified as an important confounder in many epidemiological studies. When asking how many drinks a participant usually has it may help to ask, how many pints of beer/ glasses of wine/ glasses of spirit etc so to help them provide an estimated number. The types of alcoholic beverage do not need to be noted. The researcer should highlight that the samples will not be analyzed for alcohol (nor for prescription or illegal drugs).

- **Consumption of other beverages:** This question assesses the possible exposure to chemicals via other beverages (like coffee, tea and energy drinks). Energy drinks will include, for example, sports drinks or gels, Red Bull or other caffinated drinks. Circle which additional beverages are consumed and for those indicated how many times in a typical working day.

- **Dietary habits:** Certain foodstuffs can be the source of exposure to chemicals. Other dietary habits may include gluten free, lactose free diets and should be recorded as free text.

- **Use of food supplements**: Diet pills may contain chromium. Human metabolism of some xenobiotics could be affected/modulated by the concentration of vitamins.

- **Recreational activities or hobbies:** Some recreational activities may result in exposure to metals and others substances that are going to be studied in the scope of this project. If the respondent indicates that they do have recreational activities that may cause additional exposure, details of what they do and the substances they use should be recorded.

- **COVID-19 Symptoms and diagnosis:** This information is collected since recent viral infection and vaccination will interfere in the inflammatory markers to be analysed.

2. Occupational history (to be completed for both exposed workers and control group)

The information on occupational history and the exposure years are used when assessing the total cumulative exposure. This is important especially with the accumulative chemicals as their effects may show up even after long lag period. Please list all the possible work periods lasting more than 12 months in the activities mentioned.

Researcher should start by asking about the respondents' current job (mentioning that this will be discussed in more detail if they are an exposed worker) and then ask the respondent to work back from this job through the jobs they have had. As a prompt it may be helpful to ask what year the respondent left school to ensure that all work periods are covered. In the event that the respondents work did not involve any of the activities of interest the activity boxes should be left unticked. In the event that the respondent did not work during a particular time period, for example, due to a period of study, unemployment or maternity leave this should be recorded as 'not employed' for the time period in question with the activity boxes left unticked.

Start and finish years should be recorded as YYYY, e.g. 1990.

Job description – The researcher should record which work task was performed by the participant which will then guide the next set of questions to be asked.

In the event that the respondent indicates that they did not complete any of these activities (in other words they are part of the control group), they should be advised that the questionnaire is complete and thanked for their contribution to the project.

4. Job descriptions for different e-waste activities (to be completed for only the exposed workers)

Job descriptions in E-waste (interviewed by researcher during the sampling)

- Please select all type of work tasks the worker has been involved in today and not only the main task. Please consider that the information is divided into three different processes, namely E-waste recycling, hydrometallurgical processing and biometallurgical processing.
- Ask the requested contextual information:
    - Duration (hours/min) and frequency of the tasks (times per week)
    - Process type – record manual or automatic.
    - Risk management by personal protection equipment (PPE) and respiratory protective equipment (RPE). Show Flash Card 1 (see Appendix 1) to assist respondent in their response concerning PPE use and record the relevant numbers on the form. If 'other' PPE is used, record '8' and details of what was used.
    - If local exhaust ventilation (LEV) was in use or not as this is very important in assessing adequate risk management of exposure. Record 'yes' or 'no'
- It is important to know whether the RPE (mask) has been fit tested. To assist the respondent fit testing can be explained as a method of checking that a tight-fitting face piece matches the wearer's facial features and seals adequately to their face. If they respond yes, that they have been face fit tested the year of testing should be recorded as 4-digits, e.g. 2017.
- Record as 'yes' or 'no' whether the worker has received information, instruction or training on the use of safe working practices for this activity
- Hygiene facilities – tick all that apply. In the event that 'other' is indicated, further details should be recorded as free text.
- If the work conditions were not normal, please specify all the possible problems during the working day (e.g. problems with mask or extraction not working) as this may impact on the level of exposure.

Completion of questionnaire

Upon completion of the questionnaire, participants should be informed that the interview is now complete and thanked for their contribution to the project.

5. Storing and reporting of gathered information

The gathered hard copy questionnaires should be placed in secure storage in the manner described below, which is accessible only to designated members of the project team.

The front page of the worker questionnaire, which contains the personal information, should be removed from the rest of the questionnaire. Before doing so check that the remaining pages of the worker questionnaire have the relevant unique identification codes entered. The first page of the worker questionnaire should be stored in a secure physical storage, separate from the rest of the coded questionnaire so to ensure confidentiality of the collected information.

The company questionnaire should also be stored in a secure physical storage. The company questionnaire should remain intact, e.g. there is no need to remove the front page.

The hard copy questionnaire data should be entered into the central electronic template in a timely manner. Care must be taken to follow the instructions accompanying the data template. The template must NOT be modified in any way - DO NOT add / remove columns, or alter the drop down lists, or merge cells. In the event that the template is modified or data has been provided which does not follow the instructions, templates will be returned to the data provider for correction.

Both hard copy and electronic data should be archived in compliance with relevant national and European data protection legislation.

References

Porras S, Ladeira C, Ribeiro E, Viegas S, Uuksulainen S, Santonen T, Galea K, Cherrie J, Louro H, Ventura C, Silva MJ, Leese E, Jones K, Hanser O, Ndaw S, Robert A, Duca R-C, Poels K, Godderis L, Kiilunen M, Norppa H, Veijalainen H, Parshintsev E, Tuomi T, Ruggieri F, Alimonti A, Koch H, Bousoumah R, Antoine G, Jacoby N, Musgrove D. (2019) HBM4EU occupational biomonitoring study on hexavalent chromium and other harmful chemicals. Standard Operating Procedures (SOPs). WP8 - Targeted field work surveys and alignment at EU level. Available from URL: https://www.hbm4eu.eu/online-library/ (last accessed 22nd February 2021).

Appendix 1: Flash Card 1: PPE and RPE used for e-waste

PPE (Personal protective equipment) worn:

1. Re-usable working clothes (coveralls)
2. Disposable working clothes (protection suit)
3. Working shoes / boots
4. Arm guards
5. Cut resistant gloves
6. Reusable gloves
7. Disposable gloves
8. Safety glasses/goggles
9. Face protection
10. Other (please specify)

RPE (respiratory protective equipment) worn:

1. Reusable particulate respirator (please specify how often the filters are changed)
2. Disposable particulate respirator
3. Other respiratory protection equipment (please specify: e.g. vapors protection besides particles)

Supplementary material S5.3

SOP-3 Blood sampling, including sample storage and transfer

Authors and Acknowledgements

Lead authors

Ana Maria Tavares, Henriqueta Louro, Célia Ventura, Maria João Silva, National Institute of Health Dr. Ricardo Jorge (INSA), Lisbon, Portugal.

Contributions were received from:

Radia Bousoumah (INRS, France), Sophie Ndaw (INRS), Adrian Covaci (UAntwerpen, Belgium), Thomas Göen (IPASUM, Germany), Paul Scheepers (Radboudumc, the Netherlands), Elizabeth Leese (HSE, UK), Jelle Verdonck (KuLeuven, Belgium) and Simo Porras (FIOH, Finland).

The text has been partially adapted for the E-waste occupational study based on the document SOP 3: Procedure for obtaining human samples, prepared under WP 7, Task 7.2, D 7.3.

The draft version was revised upon suggestions made by laboratories with expertise in E-waste analysis.

This document has been created for the HBM4EU project. HBM4EU has received funding from the European Union's Horizon 2020 research and innovation programme under grant agreement No 733032.

Introduction

Within the Human Biomonitoring Initiative (HBM4EU project), several priority chemicals were identified, which may be of concern for the European population.

E-waste stream contains many composite materials such as circuit boards, cathode ray tubes, flat screen monitors, batteries, connectors and transformers, plastic casings, and cables. These materials render a broad range of hazardous ingredients, including toxic metals, polybrominated diphenyl ether (PBDE) and organophosphate ester (OPE) flame retardants, phthalates, polychlorinated biphenyls (PCBs), hexabromocyclododecanes (HBCDs), polychlorinated dibenzo-*p*-dioxins (PCDD), polybrominated dibenzo-*p*-dioxins (PBDD) and polychlorinated dibenzofurans (PCDF). Plastic materials may contain chemicals that were legal at the time they were manufactured but are now either restricted or banned, such as lead, PCBs, some phthalates, and flame retardants (Grant et al. 2013). Occupational exposure to a mixture of such pollutants during e-waste handling and recycling is a matter of concern at European workplaces.

In the context of the present occupational study, biomarkers of exposure to chromium (Cr), cadmium (Cd), mercury (Hg), lead (Pb), brominated and organophosphate flame retardants (BFRs and OFRs), polychlorinated biphenyls (PCBs) and phthalates will be analysed in biological specimens of workers involved in different steps in the chain of waste processing. Most of these chemicals can damage DNA, resulting in genome instability, which, in turn, is a crucial event in cell transformation towards malignancy. According to the International Agency for Research on Cancer (IARC) classification, Cr(VI), Cd and PCBs are carcinogenic to humans, while Pb is classified as probably carcinogenic (Bakhiyi et al. 2018). Although the mechanisms underlying the carcinogenetic effect are still unclear for some of these substances (and mixtures), indirect genotoxicity due to oxidative damage to nucleobases, induction of membrane lipid peroxidation, DNA methylation, and dysfunction of DNA repair have been shown (Wang et al. 2018). Accordingly, a significant relationship between the duration of exposure to e-waste and DNA damage in lymphocytes and spermatozoa among

recycling e-waste workers was recently reported (Wang et al. 2018). Based on this knowledge, the analysis of biomarkers of early biological effects (effect biomarkers) was also included in this study.

To summarise, blood will be the biological specimen used to analyse the following biomarkers: i) Cr, Cd, Pb, PCBs, and BFRs (exposure biomarkers), and ii) micronuclei (MN) in peripheral blood lymphocytes (PBLs) and reticulocytes, epigenetic markers, oxidative stress, telomere length and inflammation markers (effect biomarkers).

This Standard Operating Procedure (SOP) for blood sampling provides the general procedure for the collection, storage and transfer of human blood samples to be analysed within the e-waste occupational study under HBM4EU and is based on a SOP developed within a previous occupational study (Santonen et al., 2019).

Precautions in the pre-analytical phase

The pre-analytical phase comprises all actions and aspects that occur prior to the analytical phase and should be considered as part of the laboratory work. This phase involves the collection, handling, transport and conservation, distribution, and storage of samples until analyses, which this SOP addresses.

It is essential to avoid, or at least minimise, samples misidentification and possible sources of contamination. In this regard, two main groups of factors should be considered:

Influencing factors:
Metal atoms can form ionic, covalent, and coordinate bonds. Ligands containing oxygen, nitrogen, or sulfur are preferentially bonded. Consequently, many important biological compounds, such as proteins and nucleic acids, are targets for an interaction with metals. If the ligands are organic molecules with more than one group capable of coordination, metals can form stable complexes known as chelates (examples of chelating agents: BAL, EDTA, DMPS)1 (Greim and Snyder 2008). These properties must be taken into account when choosing the materials used for sample collection and analysis. Moreover, workers can be exposed to common e-waste chemicals through other sources besides occupational ones, e.g., food (Cd, BFRs and PCBs), and water (Cr, Cd, and Pb) (Grant et al. 2013). Furthermore, some of the chemicals, particularly, Cr and Cd, can also be inhaled from tobacco smoke. Therefore, apart from the exposure in occupational settings, also the exposure of workers through the referred sources should be assessed by the questionnaire.

Samples identification will be approached in more detailed below (see 3.4).

Interfering factors

It is essential to identify and avoid possible sources of external contamination at the sampling site, as well as sample contamination due to inappropriate skin disinfection or to the use of non-sterile equipment/materials. Likewise, alterations due to adsorption of the substances under analysis to the vials wall should be also avoided.

Particularly, to reduce interferences in analysis the following recommendations must be followed:

[1] BAL : British Anti-Lewisite, EDTA : Éthylene diamine tetraacetique acid, DMPS : 2,3-dimercapto-1-propanesulphonic acid

- The skin should be disinfected with alcohol and not with povidone iodine;
- Powder-free gloves should be used;
- For Cr, Pb and Cd analyses, appropriate tubes "for trace element" detection, "metal free" or "for lead testing" should be used to collect blood in order to eliminate any possible metals contamination (CDC 2018);
- EDTA anticoagulant is preferable (CDC, 2018);
- For venous blood collection it is recommended to use needles coated inside with silicone, although this is not consensual.

In addition, good aseptic techniques should always be employed in the collection of blood samples.

Blood Sampling

Blood collection schedule

One blood sample will be collected from each (exposed or non-exposed) worker, following signed informed consent of the participant (see SOP 1: Selection of participants and recruitment, information to the participants, informed consent).

The optimum timing for the sampling would be on the 3rd - 5th day of a working week (assuming a 5-day working week). In addition, because samples have to be processed within 24 h for Cr determination in RBC or to be shipped to the laboratories that will perform the biomarkers analyses, **blood collections should be done between Wednesday and Friday and shipped on Wednesday or Monday, respectively**, to avoid being in transit for a long time, especially on weekend (see Annex 2, fig. A.1). **Please inform in advance by email the laboratories that are going to receive samples about the number, date of samples collection and date estimated for samples arrival.**

Basic information shall be collected through an individual questionnaire with the support of the researcher or technician to avoid interpretation errors [see SOP2: procedure for completion of company and worker questionnaires].

In addition, at the sampling time, **the following information should be recorded in the Blood Sampling Form** (Annex 1):

- Unique sample code attributed to worker and used to label sample tubes, for unambiguous identification of the specimens and related documents (questionnaires, personal data, etc.)
- Date and time of blood collection
- Number of tubes collected and their destination (according to the type of analysis and Lab that will perform it).

Note: A **Material Transfer Agreement** has to be previously signed between the laboratories that will exchange blood samples for analyses.

Sampling material

The following materials and equipment will be necessary for blood sampling and fractionation:

- **Tubes with anticoagulant**:
  - 2 Tubes - **tubes 1 and 5** - with sodium heparin (volume: 3 mL per tube) for cytogenetic effect biomarkers (micronuclei);
  - 1 Tube – **tube 2** – with K_2 EDTA (volume: 3 mL) for DNA-based effect biomarkers (epigenetics, telomere length); appropriate for -80ºC (e.g., cryotubes Bio-one);
  - 2 Tube - **tubes 3 and 4** - with K_2 EDTA (volume: 3 mL in tube 3 and 6 mL in tube 4). Tube 3 will be used for analysis of Cd and Pb in total blood while tube 4 will be centrifuged to separate plasma and red blood cells (RBC) for BFRs and PCBs measurement in plasma, inflammation markers in plasma and Cr in RBC (RBC-Cr, only for workers with high U-Cr) (see details in section 4.1). Tubes for trace elements should be used to minimise the background contamination, e.g. *Greiner* Vacuette® Trace Elements, 3 ml or BD Vacutainer® Trace Element tubes (royal blue stopper)

- Vials for plasma and RBC storage must be suitable for trace elements (metal free) (e.g., ICP-MS autosampler tubes) or pre-treated with HNO3 [see 2.b)]; vials for plasma that will be used for BFRs and PCBs determination should be first rinsed with hexane [see 4.1)]
- Regular phlebotomy syringe with a stainless-steel needle; the use of a silicone-coated needle or butterfly is recommended (e.g. Sarstedt 21G for metal analysis ref. 85.1162.600); the vacutainer system can be optionally used
- powder-free disposable gloves
- 70% alcohol swabs for skin disinfection
- Labels
- Garrottes/tourniquets
- Adhesive bandages or tapes
- Container for disposal of used needles after venepuncture
- Bench centrifuge, refrigerated
- Pipettes for collecting the plasma and buffy coat
- NaCl solution (0.9%)
- Refrigerator for samples storage at +4ºC
- Dry ice
- Freezer samples for storage at -80°C
- Containers appropriate for blood samples shipment (at + 4ºC and -80ºC)

Instructions for blood sampling

The collection of blood samples requires a clean, quiet and confined space, the availability of sterile material for blood collection and staff trained in phlebotomy knowing the special precautions related to the handling of biological material, according to each country rules.

Blood sampling must only be done by personnel trained in phlebotomy techniques. In general, the blood is collected by venous puncture and manipulated under sterile conditions. The trained personnel shall be in charge of the procedure and shall use adequate personal protection equipment (lab coat and gloves). WHO (2010) provides the best practices on drawing blood and these should be followed (Annex 3). In general:

1. Keep the blood handling area clean and free of dust

2. Use only the supplies provided by the study responsible as detailed in Section 3.2; wear talc-free gloves
3. Prepare the 5 tubes and label them with the code number and other relevant information (date, time of collection)
4. Record relevant details in the record form (Annex 1 and Annex 2, fig. A.2);
5. Prepare the volunteer for phlebotomy;
6. Place the garrotte in the forearm and disinfect the collection site with 70% alcohol;
7. Collect approximately 18 mL (see table I) of venous blood by phlebotomy, loosen the garrotte and press a cotton ball with 70% alcohol against the puncture site;
8. Immediately distribute the blood from the syringe into the 5 labelled tubes, filling them to the mark to avoid the risk of haemolysis. **Tubes 3 and 4 should be the 1st tubes to be filled** to avoid contamination of phlebotomy needle when puncturing the rubber stopper of other tubes;
9. Invert each tube gently 8 times, in order to mix the sample with the anticoagulant. After mixing, keep tube 3 upright until further processing to avoid contact with stopper;
10. Check that the worker is okay and provide a plaster for puncture site as necessary.

Table I: Distribution of samples according to the analyses to be performed.

		**Tube 1**	**Tube 2**	**Tube 3***	**Tube 4***	**Tube 5**	**Volume of Blood collected**
		**Na heparin**	**K_2 EDTA**	**K_2 EDTA**	**K_2 EDTA**	**Na heparin**	
**Use**	**Fraction/ volume**	**3 mL**	**3 mL**	**3 mL**	**6 mL**	**3 mL**	
B-Pb and B-Cd	Whole blood	0	0	**X**	0	0	
PCBs and BFRs	Plasma	0	0	**0**	**X**	0	
RBC-Cr**	RBC	0	0	0	**X**	0	**18 mL**
Micronucleus in PBL	Whole blood	**X**	0	0	0	0	
Micronucleus in RET	Reticulocytes	0	0	0	**0**	**X**	
Oxidative stress	Whole blood	0	**X**	0	0	0	
Epigenetics	Whole blood	0	**X**	0	0	0	
Inflammatory markers		0	0	0	**X**	0	

Important: *Tubes 3 and 4 must be appropriate for trace elements analysis and must be the 1st to be filled; ** store the RBC samples and analyse (or send to the analysing laboratory) only those which had elevated U-Cr (limit value to be defined)*

A scheme for blood distribution among tubes and for shipment is additionally provided in Annex 2 (fig. A.3).

Sample traceability

A standardised convention will be used to assign unique identification codes for all samples collected. The identification code convention is as follows:

E-waste (E) - Country ID (XX) - Company ID (XX) - Participant ID (XXX) - Sample ID (AX/BX/LCX/RCX/HX/SDX/UX/WX/WBX)

'E' is to denote that the samples and data relate to the e-waste occupational study.

1. Country ID 'XX' is the country code, using the ISO Alpha-2 country codes for the participating countries (http://www.nationsonline.org/oneworld/country_code_list.htm).

Country	ISO Alpha-2 country codes
Belgium	BE
Finland	FI
Germany	DE
Latvia	LV
Luxembourg	LU
Poland	PL
Portugal	PT
The Netherlands	NL
United Kingdom	UK

Company ID 'XX' is a two-digit running number of companies in each country (e.g. 01 for the first company recruited, 02 the second and so forth).

Participant ID 'XXX' is a three-digit running number of participants in each country (e.g. 001 for the first participant recruited, 002 the second and so forth).

Sample ID 'BX' where B denotes the type of sample collected (blood), followed by an one-digit identifier (X) to identify the running number of each type of sample for that worker (e.g. 1 for the first sample, 2 for the second and so forth).

The following scenario is provided to illustrate the application of this convention. A worker is recruited in Portugal. He is working in the first company recruited. He is the first worker recruited in that company and is providing a first blood sample. The sample identification code assigned is therefore E-PT-01-001-B1

Conservation, transport, and storage of the samples

Processing and onsite storage of collected blood samples

All tubes should be transported from the site of collection to the nearest laboratory (local laboratory in the country of origin), as soon as possible (preferentially less than 2h after sampling), for further processing and/or expedition to the different partner laboratories involved in biomarkers' analyses.

The above referred transportation should occur at +4ºC (max +10°C) using frozen ice packs placed at the bottom and along the sides of the styrofoam box, but making sure that the samples do not freeze (Annex 2, fig. A.4). Tube **2** can equally be transported at +4 ºC and immediately frozen at –20ºC or -80ºC after arrival to the local laboratory or can be immediately frozen at -80ºC at the site of collection (and transported at that temperature). Please note that tube 2 must be compatible with -80ºC storage (e.g., cryotubes Bio-one).

For onsite storage in the local laboratory until shipment the following procedure must be followed immediately after arrival:

- **Check the blood sampling form** to confirm the number and type of tubes received per individual and to proceed accordingly.

- **Tube 1** - keep at room temperature protected from light (wrapped in aluminium foil) until shipment to INSA (Portugal).

- **Tube 2** - keep frozen at -80°C until shipment to KuLeuven with dry ice or until DNA extraction and DNA shipment to KuLeuven.

- **Tube 3** – keep at +4ºC until expedition to the analytical laboratory for analysis of Pb and Cd.

- **Tube 4** - will be used for plasma and RBC separation preferably within 8 h of the specimen collection, maximum 24 h, to avoid haemolysis. The minimum volume of plasma needed for PCBs and BFRs analyses is 2 mL. The remaining shall be criopreserved at – 80°C to analyse inflammation markers. Separation can be done as described by Devoy et al. (2016) to allow the use of RCB for Cr determination. Briefly:

    1. Record the volume V_i of the total blood in the tube (or mark the blood volume on the tube) and determine the haematocrit 1 (HT1);
    2. Centrifuge the total blood sample for 10 min at 1 000–2 000 x g (or 5 min at 2700 x g)
    3. Separate 2 mL of the supernatant (plasma 1) in a PP tube (beforehand washed with hexane and rinsed with purified water) and the remaining volume (plasma 2) in another PP tube; care should be taken to avoid collection of RBC;
    4. Dilute the RBC pellet with NaCl solution (0.9%) up to the initial volume Vi;
    5. Gently agitate at room temperature for 10 min;
    6. Centrifuge for 10 min at 1 000–2 000 x g (or 5 min at 2700 x g);

7. Discard the supernatant (washing phase);
8. Perform 2 more washings. Before the last centrifugation, measure the HT2[2];
9. After removing the last washing phase, fill the tube containing RBCs with 1% Triton X-100 in deionised water/0.2% HNO33 up to the initial volume V.
10. Storage: RBC - store at room temperature up to 3 days or at -20°C for longer periods. It is possible to keep the samples at -20°C for 3 months.

- **Tube 5** - keep at +4 - 8 ºC (do not freeze) protected from light and send to FIOH (Finland).

Transportation of the samples to the analytical laboratories

As a general rule, samples should be shipped to the laboratory that will perform analyses as soon as possible. During transportation, the storage conditions recommended above should be maintained (see Annex 2, fig. A.4), as follows:

- **Tube 1** - Pack the whole blood samples at +4°C (max +10°C) while assuring that they do not freeze during transportation to INSA (Portugal). They must arrive within 1–4 days after sampling

- **Tube 2** - Pack the frozen whole blood samples with dry ice (-80°C) and ship them to K Leuven (KU Leuven will then send whole blood to NIOM, Poland) or send isolated DNA at -20°C.

- **Tube 3** - Pack the whole blood samples at +4°C and ship them to the analytical laboratories, if different from the local laboratory

- **Tube 4** - Following plasma separation and RBC preparation (fractions can be refrigerated up to 3 days) – send RBC to the local laboratory for Cr-RBC measurement and ship the plasma 1 vial (minimum 2 mL) refrigerated (+4ºC) to University of Antwerp for PCBs and BFRs analyses; send plasma 2 at -80 ºC to KU Leuven for inflammation markers.

- **Tube 5** – Deliver the sample to the genotoxicology laboratory, FIOH within one week (preferably 1-4 days) after the sampling. The samples should be transported protected from light at +4°C (max +10°C).

To **ensure samples transportation at +4°C** (max +10°C) ice packs shall be used, placed at the bottom and along the sides of the styrofoam box, making sure, however, that the samples will not freeze. To

---

[2] The HT2 value allows to convert the final Cr concentration from µg/L of the sample analysed into µg/L of RBC. The ratio HT2:HT1 allows the correction of the RBCs loss along the washing steps.

[3] The % of HNO_3 must not exceed 0.2%, otherwise the sample coagulates. Prepare the solution (1L) with 10 mL of triton X-100 and 2 mL HNO_3 in deionised water. The solution must be sonicated 1 h in a bath to dissolve Triton completely. This final procedure is adequate if Cr is going to be measured by Atomic Absorption Spectrophotometry with Graphite Furnace. For an ICP-MS analysis the Triton/HNO_3 must be replaced by 1% Triton X-100/0.2% NH_4OH.

ensure transportation at -80°C, samples shall be immersed in dry ice using an adequate styrofoam box (see Annex 2, fig. A.4).

A shipping date should be previously arranged between the sample collectors and the laboratory. When arrangements have been finalized, the addressee should be informed of the time and means of transportation.

The deliverable report *D.7.2 "Strategy and SOPs for human sample exchange, including ethical demands"* includes all information related to the proper conservation and transport of the samples in human biomonitoring studies as well as the conditions of storage until the chemical analysis. The recommendations there referred and included in D7.2. should be followed, namely:

- Standard operating procedure for Sample Exchange on a pan-European level to be used in the HBM4EU initiative
- Shipping Category B Biological Substances
- Pro-Forma Invoice
- Sample Transfer Protocol (Manifest)
- Data Transfer Template.

The storage conditions described in the section 4.1. should be maintained until analysis, unless other specific procedure exists in the analytical lab. In addition, the blood remaining after testing will be preserved at least up to the end of the project, unless otherwise stated by national rules. Further procedures are described in each methodology SOP.

Data reporting

The data generated respects:

- The total number of workers or controls from whom blood samples were collected and the number stratified per occupational setting and per country.
- The total number of workers or controls that generated data on blood, plasma, or RBC exposure biomarkers
- the number of workers or controls that generated data on blood, plasma or RBC exposure biomarkers stratified per biomarker
- The total number of workers or controls that generated data on blood, plasma, or RBC effect biomarkers
- the number of workers or controls that generated data on blood, plasma or RBC effect biomarkers stratified per biomarker
- Values of haematocrits 1 and 2 that will be used in RBC-Cr determination.

The following information should be obtained from the laboratory undertaking the analysis which is to be entered into the data template:

- Biomarker concentration (μg/L)

  If concentration is below the limit of quantification (LOQ), the result is replaced by **<**LOQ (for example, **<0.23** if 0.23 is the LOQ). Data below LOQ should not be given as an empty cell, zero concentration or free text (i.e. <LOQ, not detected, n.d., LOQ/2) - please ensure that the **<** is used to identify the result as <LOQ.

- Analytical method used
- LOQ of the analytical method (µg/L)
- Method to calculate the LOQ (the laboratory should test that the reported LOQ concentration can be analysed accurately (RSD <20 %))
- Haematocrit 2 (HT2) values

Care must be taken to follow the instructions accompanying the data template. The template must NOT be modified in any way - DO NOT add / remove columns, or alter the drop down lists, or merge cells. In the event that the template is modified or data has been provided which does not follow the instructions, templates will be returned to the data provider for correction.

References

Bakhiyi, B,, Gravel. S., Ceballos. D., Flynn, M.A., Zayed, J. 2018. Has the Question of E-Waste Opened a Pandora's Box? An Overview of Unpredictable Issues and Challenges. *Environment International* 110: 173–92. doi:10.1016/j.envint.2017.10.021.

CDC. 2018. "Improving the Collection and Management of Human S.mples Used for Measuring Environmental Chemicals and Nutrition Indicators." Vol. 1.3. https://www.cdc.gov/biomonitoring/pdf/Human_Sample_Collection-508.pdf.

Devoy, J., Géhin, A., Müller, S., Melczer, M., Remy,A., Antoine, G., Sponne, I. 2016. Evaluation of chromium in red blood cells as an indicator of exposure to hexavalent chromium: An in vitro study. *Toxicology Letters* 255 (2016) 63–70. doi: 10.1016/j.toxlet.2016.05.008.

Grant, K., Goldizen,F.C., Sly, P.D., Brune, M.N., Neira, M., van den Berg,M., Norman, R.E. 2013. Health Consequences of Exposure to E-Waste: A Systematic Review. *The Lancet Global Health* 1 (6). doi:10.1016/S2214-109X(13)70101-3.

Greim, H., and Snyder R. 2008. Toxicology and Risk Assessment: A Comprehensive Introduction. John Wiley & Sons Ltd, England. doi:10.1002/9780470868959.

Santonen, T., Alimonti, A., Bocca, B., Duca, R.C., Galea, K.S., Godderis, L., Göen, T., et al. 2019. Setting up a collaborative European human biological monitoring study on occupational exposure to hexavalent chromium. *Environmental Research*, 177: 108583

Wang, Y., Sun, X., Fang, L., Li, K., Yang, P., Du, L., Ji, K. et al. 2018. Genomic Instability in Adult Men Involved in Processing Electronic Waste in Northern China. *Environment International* 117: 69–81. doi:10.1016/j.envint.2018.04.027.

WHO - World Health Organization. 2010. WHO guidelines on drawing blood: best practices in phlebotomy. ISBN 9789241599221. Geneva, Switzerland. https://www.ncbi.nlm.nih.gov/books/NBK138650/pdf/Bookshelf_NBK138650.pdf.

Annex 1 – Blood Sampling form

Confidential Data – do not send with sample

Worker Identification:

Country: ____

Company name and name of department: ______________________________

✂ -- ◄◄

Send with sample for analysis

***Questionnaire*:**

Date: __________

Code Number: ____________

Blood Sample:

Date: __________ Time of sampling: ____________________

Blood Code Number: ______________________________

Number of Tubes collected:__________

Volume of Blood per tube: __________ml

Destination of Tubes:

1 - ____

2- ____

3- ____

4 - ____

5 - ____

Notes/Observations:

Annex 2 – Schemes for blood collection and transportation

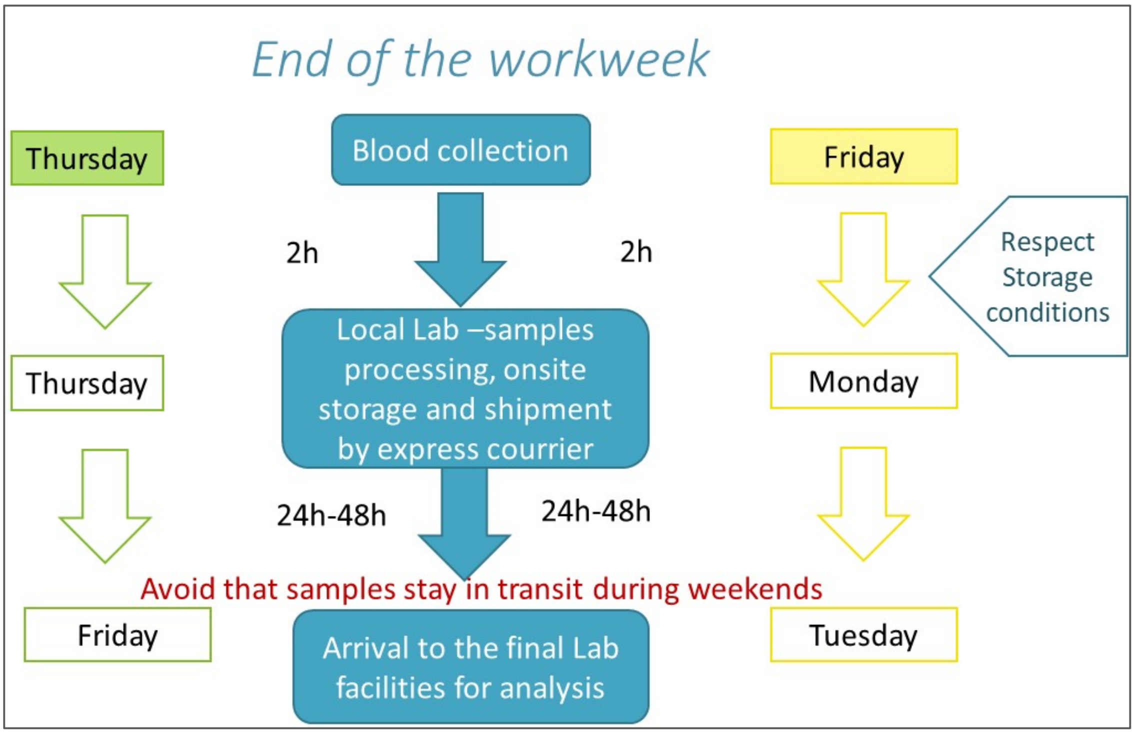

Fig. A.1 -Timing for blood collection: Wednesday or Friday afternoon

- ☐ *Informed consent* was understood and signed
- ☐ *Questionnaire was filled in and criteria for inclusion were met*
- ☐ *A code was attributed to the worker/control and to each sample*
- ☐ *The Blood Sampling Form was filled in (send to the receiver Lab)*
- ☐ *The Sample Transfer Protocol was filled in (send to the receiver Lab)*
- ☐ According to previous agreement between the samples' provider and the receiver Laboratory, the following biomarkers shall be analysed:
  - ☐ *Metals (prepare tube 3: 3mL)*
  - ☐ *BFRs, PCBs, inflammation and/or RBC-Cr (prepare tube 4: 6 mL)*
  - ☐ *Micronuclei in lymphocytes (prepare tube 1: 3 mL)*
  - ☐ *Micronuclei in reticulocytes (prepare tube 5: 3 mL)*
  - ☐ *Epigenetic markers or telomere lenght (prepare tube 2: 3 mL)*
- ☐ *Collect ______mL of blood*
- ☐ A transport box and appropriate containers are available for blood transfer

Fig. A.2 - Check list for blood collection

(A)

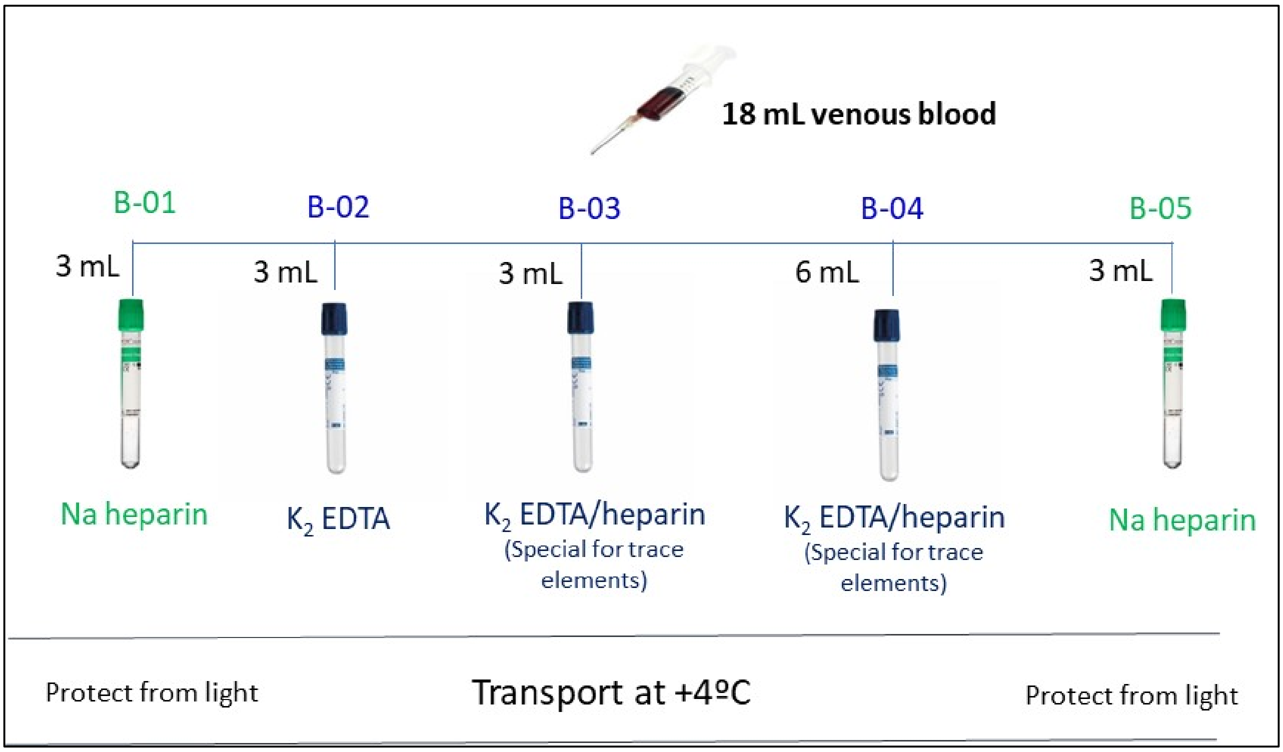

(B)

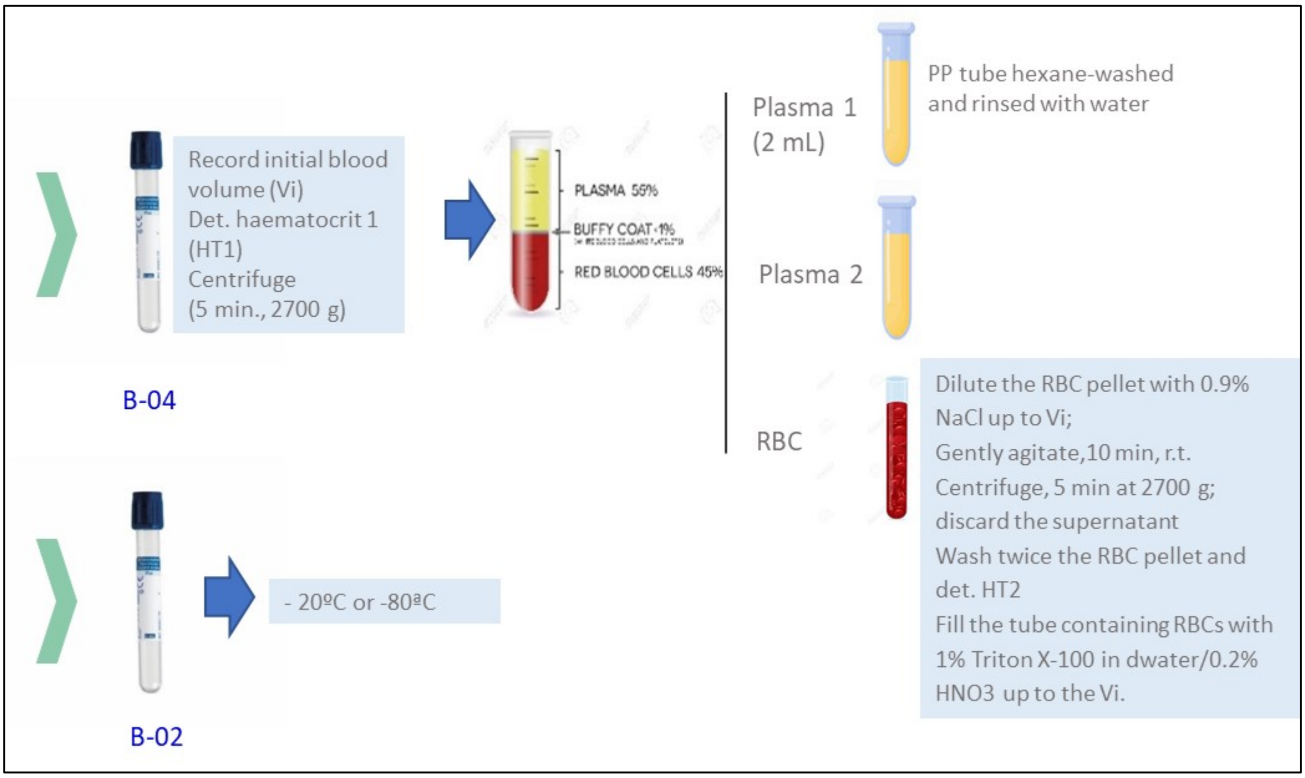

(C)

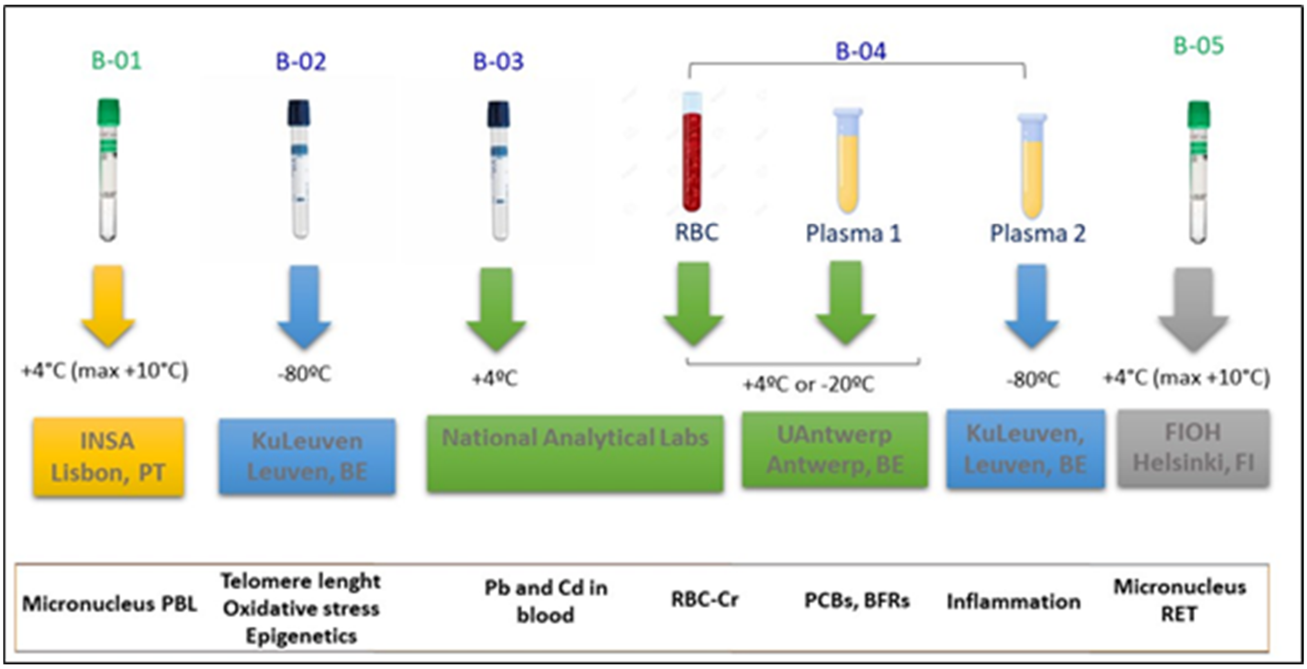

Fig. A.3 - Blood sampling, processing and transportation (A) from the collection site to the local laboratory, (B) onsite processing and (C) from the local laboratory to the laboratories where biomarkers will be analysed.

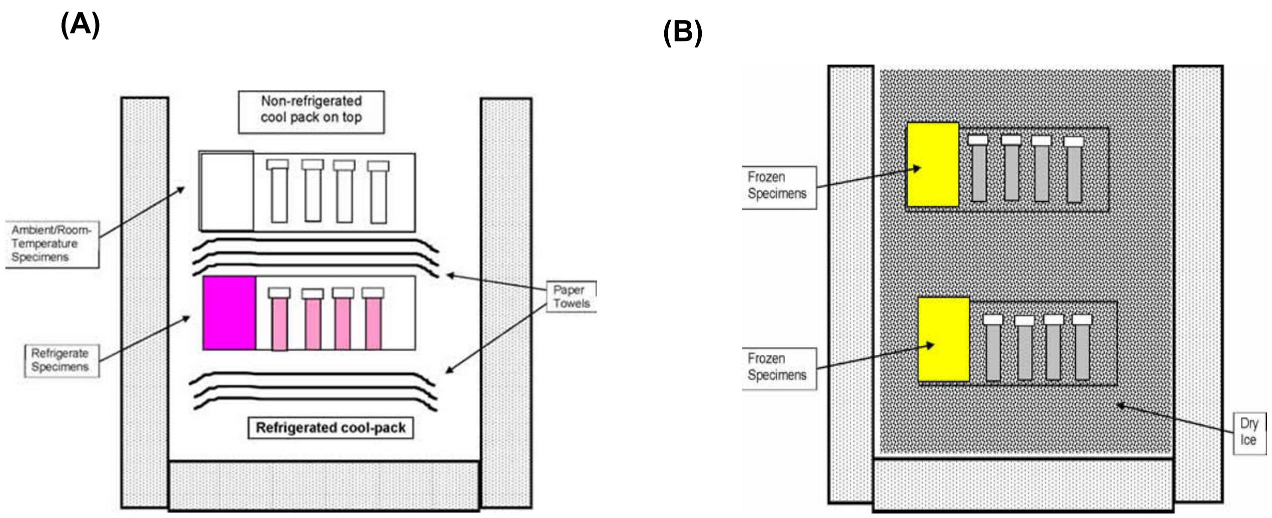

Fig. A.4 – Specimens integrity during transportation - Containers for transportation of samples (A) refrigerated and at room temperature and (B) for transportation of frozen samples (Source: Mayo Clinics)

Annex 3 - An illustration of best practices in phlebotomy (WHO, 2010)

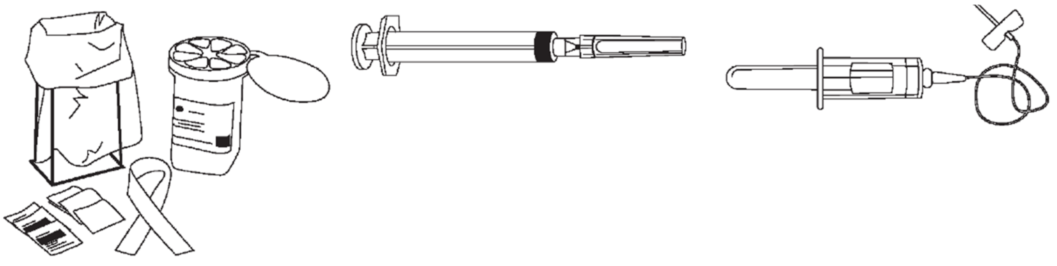

1. Assemble equipment and include needle and syringe or vacuum tube, depending on which is to be used.

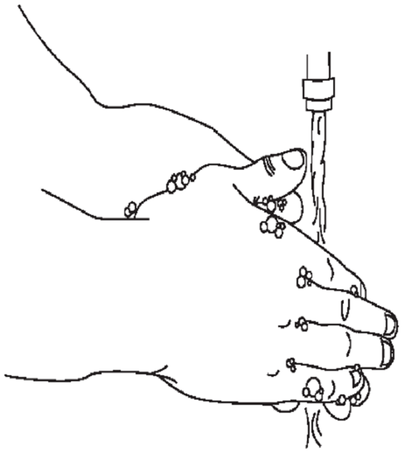

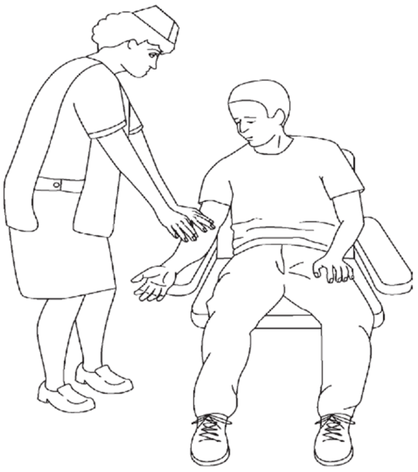

2. Perform hand hygiene (if using soap and water, dry hands with single-use towels).

3. Identify and prepare the patient.

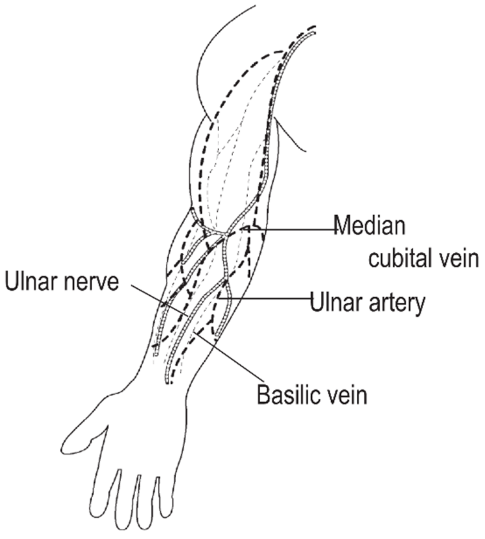

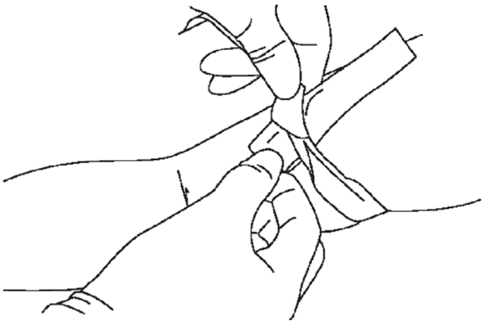

4. Select the site, preferably at the antecubital area (i.e. the bend of the elbow). Warming the arm with a hot pack, or hanging the hand down may make it easier to see the veins. Palpate the area to locate the anatomic landmarks. DO NOT touch the site once alcohol or other antiseptic has been applied.

5. Apply a tourniquet, about 4–5 finger widths above the selected venepuncture site.

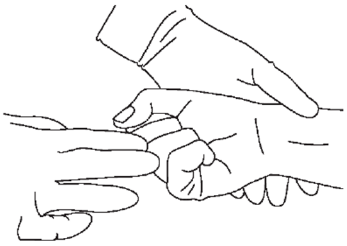

6. Ask the patient to form a fist so that the veins are more prominent.

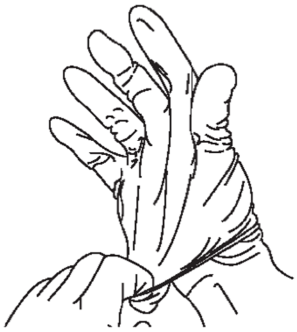

7. Put on well-fitting, non-sterile gloves.

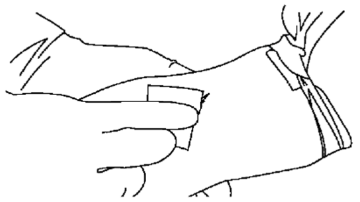

8. Disinfect the site using 70% isopropyl alcohol for 30 seconds and allow to dry completely (30 seconds).

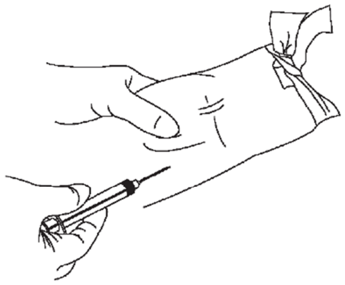

9. Anchor the vein by holding the patient's arm and placing a thumb BELOW the venepuncture site.

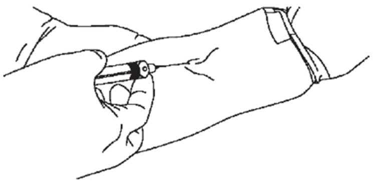

10. Enter the vein swiftly at a 30 degree angle.

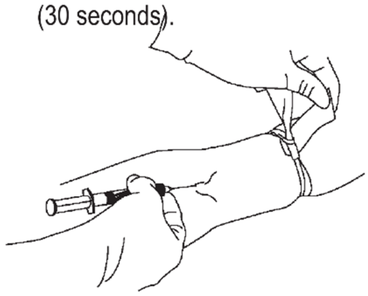

11. Once sufficient blood has been collected, release the tourniquet BEFORE withdrawing the needle.

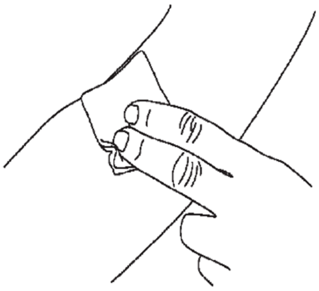

12. Withdraw the needle gently and then give the patient a clean gauze or dry cotton-wool ball to apply to the site with gentle pressure.

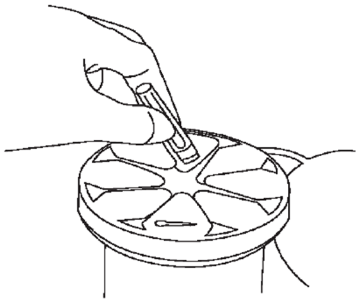

13. Discard the used needle and syringe or blood-sampling device into a puncture-resistant container.

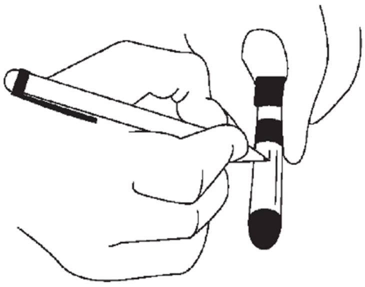

14. Check the label and forms for accuracy.

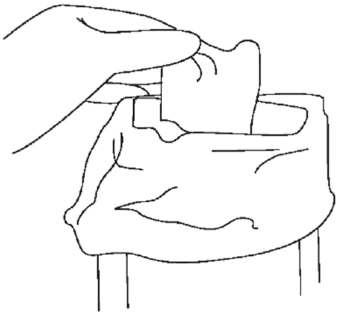

15. Discard sharps and broken glass into the sharps container. Place items that can drip blood or body fluids into the infectious waste.

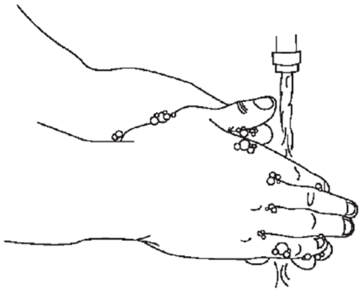

16. Remove gloves and place them in the general waste. Perform hand hygiene. If using soap and water, dry hands with single-use towels.

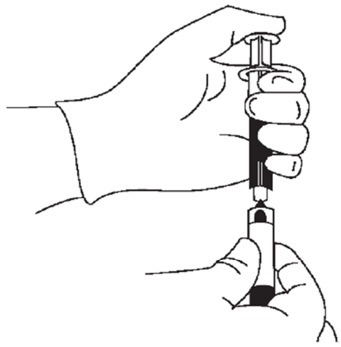

1. If the tube does not have a rubber stopper, press the plunger in slowly to reduce haemolysis (this is safer than removing the needle).

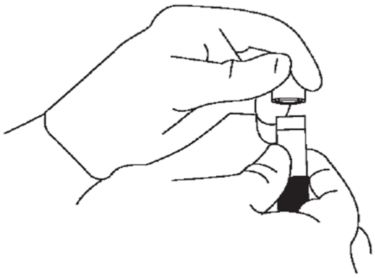

2. Place the stopper in the tube.

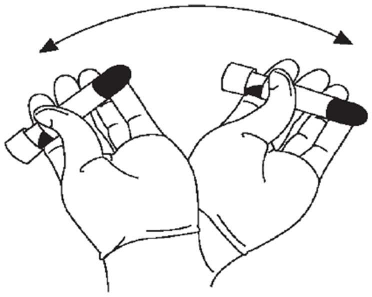

3. Following laboratory instructions, invert the sample gently to mix the additives with the blood before dispatch.

Supplementary material S5.4

SOP-4 Urine sampling, including sample storage and transfer

Authors and Acknowledgements

Lead authors:

This document has been developed by Sophie Ndaw (INRS, France), Kate Jones (HSL, UK), Radia Bousoumah (INRS).

Contributions were received from:

Karen Galea (IOM, UK), Simo Porras (FIOH, Finland), Paul Scheepers (Radboudumc, the Netherlands), Elizabeth Leese (HSL), Maurice Van Dael (Radboudumc) and Adrian Covaci (UAntwerp)

This document has been created for the HBM4EU project. HBM4EU has received funding from the European Union's Horizon 2020 research and innovation programme under grant agreement No 733032.

Scope

This Standard Operating Procedure (SOP) is focused on the collection of urine samples for the e-waste study.

When should urine sample be collected?

Urine samples will be collected from participants during the same working week.

Two urine samples will be collected from exposed workers:

- (1) before the start of the first shift of the work week (for example pre-shift on day 1).

- (2) post-shift sample towards the end of the workweek (preferably day 4 or day 5).

Controls will collect one post-shift urine sample during the working week.

Material required for urine collection and storage

Below is a list with different materials to collect and store urine samples:

- Wide-mouth polypropylene bottle (100 ml or more) for urine collection
- 5 mL polyethylene storage tube
- Labels
- Nitrile or similar disposable gloves
- Biological hermetic bags or plastic bags with a zipper
- Pipettes and tips (3 mL)
- Modesty bags
- Tube containers
- Freezers
- Cool boxes
- Posters with the hand washing and sampling procedures (Appendix 1)
- Trash cans
- Urine sample record sheet (Appendix 2)

The research team should ensure that bottles, tubes and tips are free from background contamination by metals, flame retardants or phthalates.

Participants instruction

Participants should be orally informed of the sampling procedure. In order to remind about the guidelines, a poster with the sampling procedure can be displayed in the toilet where the sampling will be done (Appendix 1). Participants will be advised of the following points before urine sampling:

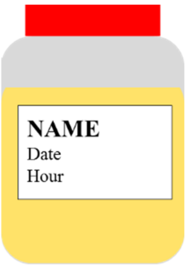

- Participants will be requested to remove their work clothes (overalls) before the urine collection;
- Participants will be asked to wash their hands thoroughly with soap and water; Hands will be dried using fresh disposable paper towels (which can be provided as necessary) or hand dryers. Reusable fabric towels must not be used.
- That they will be required to include details of their name, the date and the hour of urine collection on the bottle label.
- That the urine sample must be immediately returned to the research team.

Researcher precautions

When handling each urine sample from the participants, researchers much always wear a new pair of disposable nitrile gloves.

Care should be taken to avoid cross contamination of samples. It is recommended that urine samples are handled in an area considered to be free of potential contamination e.g. office space, medical room etc. It is also important that researchers ensure that the sampling location itself does not become contaminated.

Researchers should ensure that a site-specific risk assessment of their work practices is undertaken prior to commencing the measurement campaign and that all necessary health and safety precautions are adopted and followed.

Urine sampling procedure

The following procedures should be used to collect urine samples:

- Distribute labelled wide-mouth bottle to the participant, along with biological hermetic bags to place the bottle inside.
- Ask the participant to complete the label with the required information (name, date, hour).
- Advise the participant to remove their work clothes (overalls) and wash their hands with soap and water in accordance with the provided instructions (Appendix 1) before collecting their urine.
- Advise the participant to collect the sample of the void (does not have to be 'midstream' such as sometimes requested in hospitals)

- Advise the participant to screw firmly and to place the bottle in the biological and hermetic bag provided to avoid any leak after collecting its urine.
- The participants should return the urine sample to the research team straight away. They can use a modesty bag, left at disposal by the research team, for the transport.
- The researcher checks that the required label details are recorded and that these are correct and legible.

Urine samples processing and traceability

After collection, urine samples are homogenized and distributed in 7 aliquot tubes for analysis and storage:

- 3 aliquots of 4 mL of urine in a 5 mL tube, for
    - metals and creatinine analysis;
    - organophosphate flame retardants analysis;
    - phthalates analysis;
- 2 aliquots of 2 ml of urine in a 5 ml tube for metabolomic studies
- 2 aliquots of 4 mL of urine in a 5 mL tube, for storage.

To ensure that the aliquot tubes are properly labelled, a standardised convention will be used to assign unique identification codes for all samples collected. The identification code convention is as follows:

E-waste (E) - Country ID (XX) - Company ID (XX) - Participant ID (XXX) - Sample ID (AX/BX/LCX/RCX/HX/SDX/UX/WX/WBX)

'E' is to denote that the samples and data relate to the e-waste occupational study.

Country ID 'XX' is the country code, using the ISO Alpha-2 country codes for the participating countries (http://www.nationsonline.org/oneworld/country_code_list.htm).

Country	ISO Alpha-2 country codes
Belgium	BE
Finland	FI
Germany	DE
Latvia	LV
Luxembourg	LU
Poland	PL
Portugal	PT
The Netherlands	NL
United Kingdom	UK

Company ID ‘XX’ is a two-digit running number of companies in each country (e.g. 01 for the first company recruited, 02 the second and so forth).

Participant ID 'XXX' is a three-digit running number of participants in each country (e.g. 001 for the first participant recruited, 002 the second and so forth).

Sample ID ‘UX’ where U denotes the type of sample collected (urine), followed by an one-digit identifier (X) to identify the running number of each type of sample for that worker (e.g. 1 for the first sample, 2 for the second and so forth).

The following scenario is provided to illustrate the application of this convention. A worker is recruited in UK. He is working in the first company recruited. He is the first worker recruited in that company and is providing a first urine sample. The sample identification code assigned is therefore E-UK-01-001-U1

The urine sample record sheet should be completed by the researcher (Appendix 2). Following item are recorded:

- Sample code
- Date
- Hour
- Name of the participant
- Number of aliquot tubes for analysis and storage.

Used tips, used washing solution and urine collection containers are thrown in the trash can. The researchers collect all the used trash cans to dispose of them according to their standard practice.

Urine aliquot tubes are then stored on site in freezer at -20°C. If immediate urine processing on site is not possible, urine samples can be transferred in the HBM4EU research team laboratory to perform the processing. In this case, urine samples can be transferred in a cool box. The transfer of the samples and processing should be scheduled for the same day.

Urine sample storage

Urine aliquot tubes are stored at -20°C in each participant laboratory until analysis or transfer to another laboratory for analysis.

Urine sample transfer

To ensure samples transportation at -20°C samples shall be immersed in dry ice using an adequate box.

A shipping date should be arranged between the sample collectors and the laboratory. When arrangements have been finalized, the addressee should be informed of the time and means of transportation.

The deliverable report D.7.2 "Strategy and SOPs for human sample exchange, including ethical demands" includes all information related to the proper conservation and transport of the samples in human biomonitoring studies as well as the conditions of storage until the chemical analysis. The recommendations there referred and included in D7.2. should be followed, namely:

Standard operating procedure for Sample Exchange on a pan-European level to be used in the HBM4EU initiative

-Shipping Category B Biological Substances
-Pro-Forma Invoice
-Sample Transfer Protocol (Manifest)
-Data Transfer Template.

For the analysis of organophosphate flame-retardants biomarkers, samples will be transferred to IPASUM. For the analysis of phthalates biomarkers, samples will be transferred to the University of Antwerpen. For the analysis of effect biomarkers, sample will be transferred to NIOM.

Data reporting

The following information should be obtained from the laboratory undertaking the analysis which is to be entered into the data template:

- Urinary concentration of samples (µg/L)
  - If concentration is below the limit of quantification (LOQ), the result is replaced by <LOQ (for example, **<0.23** if 0.23 is the LOQ). Data below LOQ should not be given as an empty cell, zero concentration or free text (i.e. <LOQ, not detected, n.d., LOQ/2) - please ensure that the **<** is used to identify the result as <LOQ.
- Analytical method used
- LOQ of the analytical method (µg/L)
- Method to calculate the LOQ. The laboratory should test that the reported LOQ concentration can be analysed accurately (RSD < 20%).
- Creatinine concentration (g/L) of each urine sample
- Method to determine creatinine and LOQ (g/L) of the method

Care must be taken to follow the instructions accompanying the data template. The template must NOT be modified in any way - DO NOT add / remove columns, or alter the drop-down lists, or merge cells. If the template is modified, or data has been provided which does not follow the instructions, templates will be returned to the data provider for correction.

Below is the list of the biomarkers to be analysed in urine samples.

Chemicals	Biomarkers
Metals	chromium
	cadmium
	mercury
	lead
Organophosphate flame re-tardants	DPHP (diphenyl hydrogen phosphate)<br>BCIPP (bis(2-choropropyl) phosphate)<br>BDCIPP (bis(1,3-dichoro- -propyl) phosphate)<br>BCEP (bis(2-choroethyl) phosphate)
Phthalates	Monoethyl phthalate (MEP)<br>Monobenzl phthalate (MBzP)<br>Monoisobutyl phthalate (MiBP)<br>Mono-n-butyl phthalate (MnBP)<br>Mono(2-ethylhexyl) phthalate (MEHP)<br>Mono(2-ethyl-5-hydoxyhexyl) phthalate (5OH-MEHP)<br>Mono(2-ethyl-5-oxo-hexyl) phthalate (5oxo-MEHP)<br>Mono(2-ethyl-5-oxo-hexyl) phthalate (5cx-MEPP)<br>Mono-hydroxy-isononyl phthalate (OH-MiNP)<br>Mono-carboxy-isononyl phthalate (cx-MiNP)<br>Mono-hydroxyl-isodecyl phthalate (OH-MiDP)<br>Mono-carboxy-isodecyl phthalate (cx-MiDP)<br>cyclohexane-1,2-dicarboxylic mono hydroxyisononyl ester (OH-MINCH)<br>cyclohexane-1,2-diarboxylic mono carboxyisononyl ester (cx-MINCH)
Metabolomics in urine / effect markers	

Appendix 1: sample and hand washing procedure

1. Take a urine collection container and biological bag from the research team

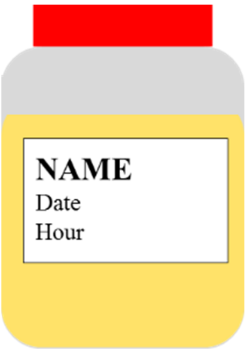

2. Complete the label with your name, date and time of collection

3. Carefully wash your hands following the instructions below

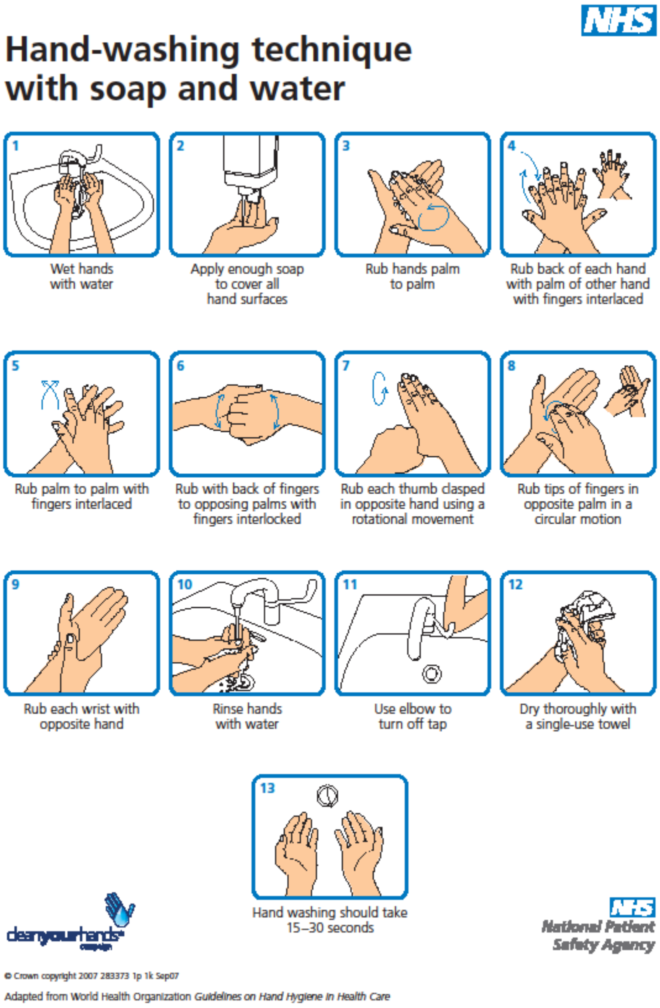

4. Open the container and collect your urine

5. Screw on the cap and place container in the biological bag

6. Immediately return the sample to the research team

Appendix 2: sample record sheet

Company Name:	
Company ID:	

Researcher(s):	
Research Organisation:	

7 Sample ID	8 Date	9 Hour	10 Name	11 Observations	12 Aliquots collected<br>13 for further analysis / storage				
14	15	16	17	18	19 1 x 4 mL metals/creatinine	20 1 x 4mL Flame retardants	21 1 x 4mL phthalates	22 1 x 4mL effect markers	23 2 x 4mL preservation
24	25	26	27	28	29	30	31	32	33
34	35	36	37	38	39	40	41	42	43
44	45	46	47	48	49	50	51	52	53
54	55	56	57	58	59	60	61	62	63
64	65	66	67	68	69	70	71	72	73
74	75	76	77	78	79	80	81	82	83
84	85	86	87	88	89	90	91	92	93
94	95	96	97	98	99	100	101	102	103
104	105	106	107	108	109	110	111	112	113
114	115	116	117	118	119	120	121	122	123
124	125	126	127	128	129	130	131	132	133

Supplementary material S5.5

SOP-5 Settled dust

Authors and Acknowledgements

Lead authors:

This document has been developed by Susana Viegas (ESTeSL), Portugal.

Contributions were received from: Karen Galea (IOM, UK), Miranda Loh (IOM), Sophie Ndaw (INRS, France), Radu Corneliu Duca (LNS, Luxembourg), Paul Scheepers (Radboudumc, the Netherlands), Simo Porras (FIOH, Finland).

This document has been created for the HBM4EU project. HBM4EU has received funding from the European Union's Horizon 2020 research and innovation programme under grant agreement No 733032.

Introduction

This Standard Operating Procedure (SOP) is focussed on the collection of settled dust samples to measure several HBM4EU priority compounds, including metals (lead, inorganic mercury, cadmium, chromium), phthalates and flame retardants in the workplaces of volunteers participating in the workplace biomonitoring studies.

In the scope of this SOP, a settled dust sample is considered to be surface/soil dust adhering to floor surfaces, normally removable by vacuum cleaners.

The purpose of this SOP is to establish a uniform procedure for collecting settled dust samples in the workplaces and is based on internal SOPs employed by IOM to evaluate exposure to metals in the home environment (Loh et al., 2015).

3. The collection and assessment of these occupational hygiene samples will provide information on the level of contamination present in the workplaces and will allow a connection with the exposure found on workers through the use of human biomonitoring (HBM) samples to be made. To sum up, the results will provide information concerning the role of the workplace environment on the workers' exposure to the substances being studied.

4.

2 Summary of the method

5. Settled dust samples are collected by field researchers during workplace visits done to collect contextual information and/or other environmental and biological samples. Vacuumed dust samples will be analysed for metals (lead, inorganic mercury, cadmium, chromium), phthalates and flame retardants (brominated and organophosphate). Samples are to be taken from the workplace where the participating workers spend the majority of their time. This is usually the location where workers perform most of their work tasks and activities. If more than one similar exposure group (SEG) is identified in a specific company, involving different tasks and waste, this might imply the need to collect a sample for more than one workplace/area. Therefore, depending on the participating workers, type of activities develop and risk management measures in place, it may imply collecting separate settled dust samples from more than one workplace area in the same company.

A minimum of 4 grams of coarse dust fraction per settled dust sample is required for analysis. The composite sample will be split and aliquoted in HBM4EU laboratories. One aliquot will be analysed for metals and then other two aliquots will be distributed for specific HBM4EU laboratories to be analysed for phthalates and flame retardants.

6. Materials required for the collection of settled dust samples

7. To undertake the collection of settled dust samples, researchers should ensure that they have the following:

1. Vacuum Supplies

- A Museum Vac (Figure 1)

- Dust collector - Filter bags (Figure 2).

- The field kit which contains:

- ✓ Pre-weighed filter bags (that should be used once for each workplace/area)
- ✓ Sample model already weighed with 4 grams
- ✓ Field sheet (Annex 1) and custody records
- ✓ A 4 m long chain or other suitable devices for measuring area for vacuuming
- ✓ Indelible labelling pen
- ✓ Clean and dry wipes
- ✓ Thermometer and Hygrometer

2. Other Collection Supplies:

- ✓ Disposable nitrile gloves
- ✓ Timer/ stop watch
- ✓ Measure tape
- ✓ Masking tape
- ✓ Zip tie
- ✓ Ziploc bag

3. Cleaning supplies

- ✓ Reagent grade isopropanol and deionized water to clean the vacuum and accessories
- ✓ Laboratory glassware detergent
- ✓ Disposable nitrile gloves
- ✓ Sheet of clean plastic (garbage bag)/Plastic tray
- ✓ Lab brushes to clean the vacuum if needed
- ✓ Toothbrush

- ✓ Tweezers
- ✓ Wide mouth glass jar or stainless-steel bucket
- ✓ New, clean, large plastic garbage bags

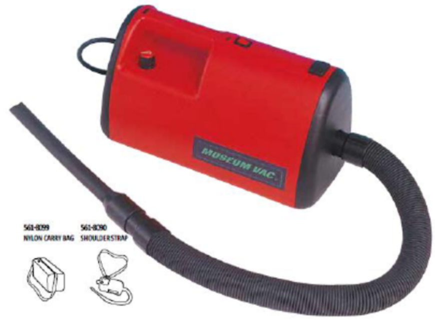

Figure 1. Museum Vac

(https://www.universityproducts.com/museum-vac-vacuum-with-dial-suction-control.html)

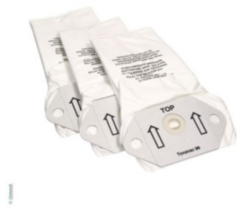

Figure 2. HEPA Filter bags

8. Procedure for defining the sampling strategy and samples collection

4.1 Sampling strategy

- Samples should be collected near the end of a full shift in a workplace occupied by workers engaged in the biomonitoring campaign and are from a specific workplace area.
- At each workplace/area sampled relevant contextual information should also be collected, namely: tasks undertaken in that area, type of waste processed and number of workers that occupied that workplace and workplace cleaning procedures (e.g. when last cleaned, type of cleaning process). This should be recorded in the Field sampling sheet (Annex 1).

4.2 Samples collection

- Make sure the vacuum crevice head tool is cleaned before every sample collection. If the team member goes from one workplace to another workplace without returning to the laboratory, the crevice head tool must be cleaned in the field.
- Clean the crevice head tool using first isopropyl alcohol (IPA) and then dry with paper towels. Use the wipes to dry both the outer and accessible inner surfaces of the sampling apparatus and allow the vacuum inlet to air dry.
- Filter bags are prepared under controlled conditions in the laboratory prior to the company visit. Filter bags are pre-weighed. This information is recorded for the entire filter (with the pre-assigned sample ID number).
- Assembly of the vacuum is performed in the company.

The following collection procedure should be followed:

- Once in the workplace, put on a pair of disposable gloves.
- Insert the pre-weighed filter bag and make sure the filter is well adjusted.
- Place the 4 m long (1 m^2) chain on the floor and FORM A SQUARE with approximately 1 m long sides. Keep the perimeter and the chain taut.
- If there is insufficient room on the floor to form a 1m^2, fold the chain to serve as the perimeter of a 0.25 m^2 square and sample multiple locations in the available space in the room, or, lay out the chain in the shape of a rectangle. NOTE: This rectangle will not provide an area of 1 m^2, thus the sides of the rectangle must be recorded to compute the true area of the rectangle. Record the dimensions (length X width) of the sampled area under "comments" on the field sheet.
- Plug in the vacuum cleaner and check for proper operation (if not doing strange noises and if the vacuum is constant). Record the temperature and relative humidity before sampling.
- Start the timer/stop watch and the vacuum simultaneously.
- Vacuum the area within the chain thoroughly. Sweeping with vacuum must be slow and deliberate but plan to cover the entire 1 m^2 in 2 minutes. Thorough coverage is essential.
- Sweep across the 1 m^2 surface with firm even pressure once in the first minute. Then change your position and sweep across the same 1 m^2 (within the chain boundary) at an angle. Sampling at orthogonal/perpendicular directions is preferable; however, when not possible due to space constraints, diagonal sampling is acceptable.
- Repeat previous steps in the same area if more dust is needed to obtain 4 grams (e.g. by visual checking and comparing using a sample model already weighed in the lab).
- Ensure the vacuum crevice head is pointing up and turn off the vacuum cleaner and move to an area where the air current is minimal. Remove the head of the crevice head and estimate that at least 4 grams of dust have been collected and if not, sample additional area.

- If the yield is sufficient, remove the filter carefully and close the open end by rolling it down. Then secure the rolled end with a piece of scotch tape and place in the Ziploc bag.
- Place the sample in the sample container (already identified with the sample numbers) and seal it. Complete the Field Sheet and Chain-of-Custody Record after placing the sampler in the transport container.
- Change the filter and repeat the procedures for other workplace/area in the same company if needed.
- Transport the sample to the laboratory protected from the light.
- Keep the dust samples at room temperature (about 20-25ºC) until analysis.

5. Sample traceability and contextual information

A standardised convention will be used to assign unique identification codes for all samples collected. The identification code convention is as follows:

E-waste (E) - Country ID (XX) - Company ID (XX) - Sample ID (SDX).

'E' is to denote that the samples and data relate to the e-waste occupational study.

Country ID 'XX' is the country code, using the ISO Alpha-2 country codes for the participating countries (http://www.nationsonline.org/oneworld/country_code_list.htm).

Country	ISO Alpha-2 country codes
Belgium	BE
Finland	FI
Germany	GE
Latvia	LV
Luxembourg	LU
The Netherlands	NL
Poland	PL
Portugal	PT
United Kingdom	UK

Company ID 'XX' is a two-digit running number of companies in each country (e.g. 01 for the first company recruited, 02 the second and so forth).

Sample ID 'SDX' where SD denotes the type of sample collected (settled dust), followed by an one-digit identifier (X) to identify the running number of sample (e.g. 1 for the first sample, 2 for the second and so forth).

The following scenario is provided to illustrate the application of this convention. A settled dust sample is collected in Portugal. It is the first sample taken in the first company recruited. The sample identification code assigned is therefore E-PT-01-SD1.

The unique sample identification code will be clearly stated on the labelled self-seal bag. A Field sheet (Annex 1) should be completed when collecting the settled dust samples.

6. Storage of collected settled dust samples

9. The collected samples should be stored in a clean box at room temperature before transportation to the laboratory.

7. Quality control

To check for contamination during the sampling procedure, transportation and storage, field blanks should be collected for each sampling survey. In this case the Filter Packet will be taken to the workplace sampling site but it will remain sealed in the Ziploc freezer bag, and experience the same transportation conditions as other valid samples both pre- and post-sample collection.

These samples should be treated in the same way as the exposure samples, using the same procedures as previously described, but omitting the vacuum procedure. The average mass found in the field blank should be subtracted from the corresponding mass found in the samples. In the event of elevated concentrations being observed in the blank samples these will be investigated and used to flag any suspect samples.

The number of field blanks should be no less than 10% of the number of samples, however, it is recommended that one field blank is collected per company.

8. Transportation of settled dust samples to laboratory

Local arrangements will need to be put in place with respect to the transportation of the samples to the laboratory. For example, in some instances the samples may be driven by the researcher to the laboratory whereas in others, courier delivery may be necessary.

Samples can be transported at room temperature to the agreed analytical laboratory ideally the same day however if this is not possible no later than the next day. Details of the numbers of samples being sent, sample identification codes, requested analysis and contact details of the responsible researcher should accompany the samples.

It is recommended that a hard copy of this information be included with the samples and that an electronic version is issued to the receiving laboratory at the time of sending the samples. This will allow sample **numbers and identification codes to be checked upon receipt at the laboratory.**

Whilst no storage stability tests have been established within the HBM4EU project it is recommended that all collected samples are analysed as soon as possible by the receiving laboratory.

9. Reporting

It is important that information entered into the sample record sheet (Appendix 1) is recorded as per the instructions given in this SOP as this information will need to be entered into the data template for the overall e-waste study.

Care must be taken to follow the instructions accompanying the data template. The structure, drop down lists etc of the data template must NOT be modified in any way. In the event that the template is modified or data has been provided which does not follow the instructions, templates will be returned to the data provider for correction.

References

UA-F-7.011. June 13, 1997 Field Collection and Post Handling of Indoor Floor Dust Samples.

HBM4EU Project – Chromates study: SOP 7: Standard operating procedure for obtaining dermal wipe samples. Available from URL: https://www.hbm4eu.eu/online-library/ (last accessed 22nd February 2021).

Lebowitz MD, 1993. Study Design (Revision of 31 Dec. 1993) EPA NHEXAS Cooperation Agreement

Loh MM, Sugeng A, Lothrop N, Klimecki W, Cox M, Wilkinson ST, Lu Z, Beamer PI, 2016. Multimedia exposures to arsenic and lead for children near an inactive mine tailings and smelter site. Environ. Res. 146, 331–339. https://doi.org/10.1016/j.envres.2015.12.011

Santonen et al., 2019. Setting up a collaborative European human biological monitoring study on occupational exposure to hexavalent chromium. Environ. Res. 177, 108583. https://doi.org/10.1016/j.envres.2019.108583

Field sheet

**Company Name:**		
**Company ID:**		
**Workplace ID:**		
**Job title/SEG:**		
**Cleaning procedures**	**Mode of cleaning**<br>Vacuum<br>Water based<br>Sweeping with a slot<br>Other (specify)<br>____________________<br>____________________<br>_____________<br>__________________	**Date/hour of last cleaning:**<br>Date: ___/___/___<br>Hour:_______ : __________

**Researcher(s):**	
**Organisation:**	
**Date sampling:**	

**Sample ID**	**Filter Weight**	**Collection duration (00:00)**	**Area sampled**	**Temperature**	**Relative humidity**	**Tasks undertaken in the workplace sampled**	**Type of waste processed**

Supplementary material S5.6

SOP-6 Air sampling of inhalable and respirable dust fraction

Authors and Acknowledgements

The contents of the current SOP draw heavily from "SOP6: Standard operating procedure for air sampling of inhalable and respirable dust fraction and (hexavalent) chromium) developed for the HBM4EU occupational biomonitoring study on hexavalent chromium and other harmful chemicals (Porras et al., 2019). As such the contributors listed here as those that contributed to this original SOP.

Lead authors:

This document has been developed by Dr Radu-Corneliu Duca (PhD) from the Unite Environmental Hygiene and Human Biomonitoring, National Health Laboratory (LNS) Luxembourg; and Dr Katrien Poels (PhD) and Prof. Lode Godderis from the Laboratory for Occupational and Environmental Health of the KU Leuven University (Belgium).

Contributions were received from:

Karen Galea (IOM, UK), Kate Jones (HSL, UK), Mirja Kiilunen, Henna Veijalainen, Evgeny Parshintsev, Simo Porras and Tapani Tuomi (FIOH, Finland) and Sophie Ndaw (INRS, France).

This document has been created for the HBM4EU project. HBM4EU has received funding from the European Union's Horizon 2020 research and innovation programme under grant agreement No 733032.

Scope

This Standard Operating Procedure (SOP) is partially based on MDHS 14/4 *"General methods for sampling and gravimetric analysis of respirable, thoracic and inhalable aerosols"* and describes the air sampling of the inhalable (total) and respirable (alveolar) dust fraction in order to assess workplace exposure.

This SOP is also partially based on internal SOPs of the KU Leuven Laboratory for Occupational and Environmental Hygiene for the determination of the inhalable / respirable dust fractions. In accordance with these SOPs, sampling of these two fractions is performed using an IOM sampler and a Higgins-Dewell cyclone respectively. These samplers are selected as they are in best agreement with the inhalable / respirable convention.

Sampling of the inhalable dust fraction is performed at a flow rate of 2 L/min with an IOM-sampler containing an IOM-cassette fitted with a pre-weighed 25 mm PVC-filter (GLA-5000) or a 25 mm MCE filter (SKC 225-1930). Filter sampling of the respirable dust fraction is performed at a flow rate of 2.2 L/min with a Higgins-Dewell type cyclone (excepting for SKC 225-69 cyclone where a 3.0 L/min flow rate should be used) containing a cyclone cassette fitted with a pre-weighed 25 mm PVC-filter (GLA-5000) or a 25 mm MCE filter (SKC 225-1930). Even though the current SOP mainly focus on the utilisation of Higgins-Dewell type cyclones, other cyclones types might be used, if the international standards are followed mainly concerning the most appropriate flow-rate. For instance, SKC GS-3 respirable dust cyclone (225-103) with

25 mm three-piece filter cassette (225-3-25LF) and a flow rate of 2.75 L/min, conform to the ISO 7708 standard, could alternatively be used. Nevertheless, the other terms of utilisation for cyclones should be in agreement with the current SOP.

After drawing air over the pre-weighed filter (cassette in the instance of the IOM sampler), the filter (cassette in the case of the IOM sampler) is re-weighed and the concentration of particulate matter is calculated from the mass difference and the sampled air volume. The metals (i.e. Cr, Cd, Hg, Pb) determination can be performed on this same filter, depending on the agreed analytical laboratory.

Depending on the agreements made with the project team and the analytical laboratories assigned to the air sample analysis, the following methods can be used for further metals analysis:

- OSHA Method ID-125G ‘Metal and metalloid particulates in workplace atmospheres (ICP analysis)’ (OSHA, 2002)
- NIOSH Method 7302 'ELEMENTS by ICP (Microwave Digestion)' (NIOSH, 2014)

Materials required for the collection of air samples

Below is a list with the different materials needed for performing air measurements. Samplers, as well as assembled IOM cassettes and cyclone cassettes (preloaded with pre-weighed filters) should be provided by the agreed analytical laboratory.

- IOM inhalable sampler, in conductive plastic (SKC 225-70A) or stainless steel (SKC 225-76A)
- IOM-cassettes (25 mm), in conductive plastic (SKC 225-71A) or stainless steel (SKC 225-75A), with transport clip and cover.
- Higgins-Dewell cyclone, in conductive plastic (JS Holdings, FH022) or SKC cyclone, in conductive plastic (SKC 225-69)
- Cyclone cassettes (25 mm), in conductive plastic, with metal support grid and transport clip (SKC 225-62)
- Spare o-rings for sampling heads
- MCE (mixed cellulose ester) filter, diameter 25 mm, pore size 0.8 μm (SKC-filter SKC 225-1930) should be used or alternatively PVC membrane filter, GLA-5000, Pall 66466, diameter 25 mm, pore size 0.8 μm, 100/package (VWR 514-0466).
- High flow air pump, capable of operating for up to 8 hours at a flow rate of 2 L/min (inhalable dust fraction with IOM sampler) or 2.2 L/min (respirable dust fraction with Higgins-Dewell cyclone) or 3 L/min (respirable dust fraction with SKC cyclone), with battery charger, e.g. Gilian GilAir-5 pump (Sensidyne), AirCheck 52 pump (SKC 224-52), Sidekick pump (SKC 224-52MTX). If other samplers are used, researchers also need to check that the pumps are capable at running at the required sampler flow rates for a period of up to 8 hours.
- Flow rate calibrator, e.g. Defender 510 (BIOS DryCal), calibrated against a primary standard, capable of measuring the required flow rates
- IOM Calibration adaptor (SKC 391-01)
- Protective pump pouches (e.g. SKC 224-88 for Sidekick pump) and belts/harnesses to allow sampling equipment to be attached to wearer

- Supply of clips to attach sampling heads to participants (if not already on sampling head)
- Sufficient lengths of flexible tubing of suitable diameter for making a leak proof connection from the sampling head to the pump
- Calibrated timepiece, to chronometrate exact sampling time
- Sample record sheets (Appendix 1)

Recommendations and precautions for air sampling

- Users of this SOP should first carry out a suitable, specific risk assessment, prior to performing air measurements. Appropriate health and safety practices should be established in order to ensure compliance with regulatory requirements.
- Sampling is preferably carried out by a person, familiar with collecting personal inhalation measurements, according to good occupational hygiene practices.
- Before air sampling is performed, the type of PVC filters or MCE filters to be used, is defined by the analysing laboratory, according to the laboratory's SOP for analysis. Assembled, pre-loaded cassettes should be provided by the same lab.
- If plastic IOM cassettes and cyclone cassettes are used these should be treated as disposables (single use only), in order to avoid any possible contamination.
- IOM and cyclone samplers should be maintained on a regular basis, and the o-rings checked each time before sampling. Samplers should be well pre-cleaned, checked for any defects and operated according to instructions of the manufacturer (see also SKC Operating Instructions: 'IOM Sampler Instructions', 'IOM O-Ring Fitting Instructions', 'Cyclone Samplers for Respirable Dust-Operating Instructions')
- Tightness of IOM-samplers should be tested secured
- When collecting air samples, researchers must always wear a new pair of powder-free disposable gloves. Care should be taken to avoid cross contamination of samples.
- Air sampling should be performed during a time period, representative for the actual working period of the exposed person, but taking into account that this should be as long as is reasonably practical.
- Air sampling should be interrupted during lunch breaks and pumps should be switched off then and removed from the wearer. The flow rates of the pumps should be checked during this period.
- Spare pumps, sampling heads and cassettes must always be provided so that planned samplings are not compromised.

Air sampling procedure

Sampling for both inhalable and respirable fraction can be performed simultaneously. Therefore, the worker will need to wear two different pumps, preferably on two different sides (right and left side), preferably by alternating the sides of inhalable and respirable fraction collection between workers.

Setting up sampling equipment

- Ensure that IOM and cyclone sampler components are cleaned of any contamination using a detergent solution. Allow the components to dry fully before use.
- Charge the pump overnight with appropriate battery charger
- Set up the pump, sampler, cassette and flow calibrator in a clean area
- Use powder-free gloves

IOM-sampler

- Remove IOM cassette (this will have been pre-weighed with the loaded filter) from its transport clip and remove protective cap.
- Unscrew top plate from IOM sampling head housing body. Ensure the O-rings are positioned correctly (Figure 1).
- Insert the IOM cassette into the IOM housing body. Screw the top plate into the housing body. Tighten securely to achieve a good seal. (Figure 2 for exploded view of an IOM sampler).

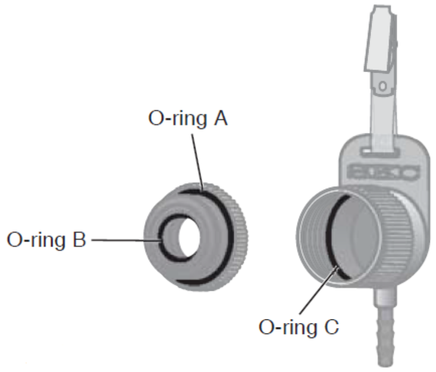

Figure 1. O-ring placement for plastic IOM sampler

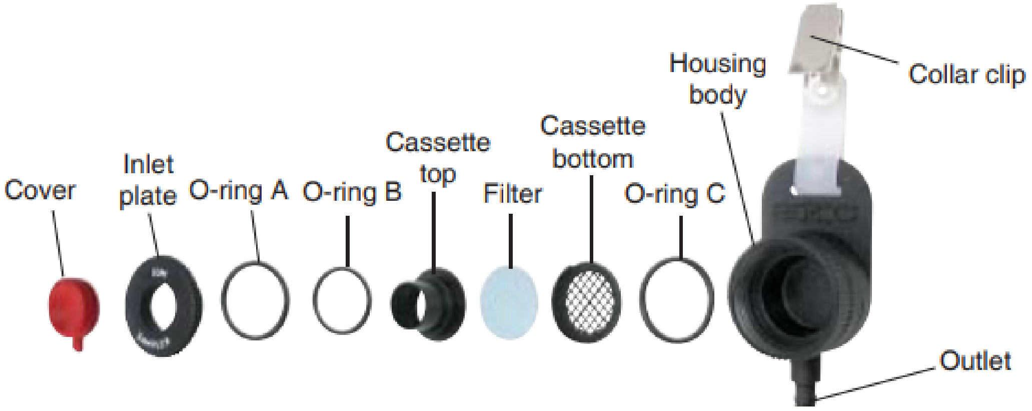

Figure 2. Configuration for plastic IOM sampler and cassette

Cyclone sampler

- Remove cyclone cassette (which contains the filter) from its sealing clip
- Unscrew cyclone sampler top from the sampler body. Ensure the O-rings are positioned correctly (see Figure 3 as way of example but please refer to the manufactures instructions of the cyclone selected for use by your institution).

- Fit the cyclone cassette into the cyclone sampler body with the cassette top upwards. Screw the sampler top into the sampler body. Tighten securely to achieve a good seal. Ensure that the clean and empty grit pot is securely fitted over the ridge around the bottom of the sampler body (see Figure 3).

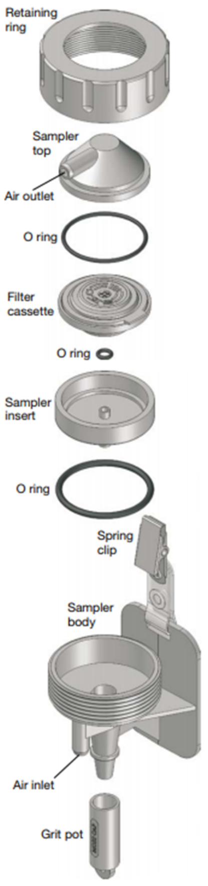

Figure 3. Configuration for plastic cyclone sampler and cassette

- Connect IOM sampler / cyclone to the pump using flexible tubing of suitable diameter for making a leak proof connection from the sampling head to the pump.
- The tubing should be of sufficient length allowing unimpeded movements of the worker.
- Switch on the pump and allow the flow to stabilise for a few minutes
- Attach flow meter to the inlet of the sampler (using a calibration adaptor for IOM sampler)
- Set the flow rate within ± 0.1 L / min of the prescribed flow rate, using a calibrated flow meter (e.g. Defender 510):
    - 2.0 L/min for IOM sampler
    - 2.2 L/min for Higgins-Dewell cyclone sampler
    - 3.0 L/min for SKC cyclone sampler
    - In the event that other samplers are being used, check the manufacturer's instructions to ensure that the correct flow rates are being used.
- Measure and record the flow rate several times (e.g. 6 replicate readings from the calibrated flow meter)

- Disconnect the flow meter
- Perform a leak test by covering the sampler's inlet or kinking the tubing
- If the pump does not stall, this could indicate a leak and should be rectified and procedure above repeated.
- Switch off the pump
- Recap IOM sampler to prevent contamination of the filter

Placement of samplers on participants

- Attach pump(s) to the worker's belt or harnesses so that they cause minimum inconvenience to the worker and safely secure the pump tubing
- Attach IOM and cyclone sampler to the worker's upper chest or lapel using the collar clips, preferably on two different sides (right and left side). Samplers should be placed in the breathing zone, not more than 30 cm away from the nose-mouth region (see Figure 6 and 7).
- Make sure the opening of the IOM cassette is not directed upwards
- Cyclone sampler should be attached with the grit pot pointing downwards
- When ready to begin sampling, remove protective cap from the IOM sampler (cyclone does not have a protective cap).
- Switch on the pump and record the time, using a calibrated timepiece
- Check the sampler and pump periodically during sampling to ensure that the equipment is still working.
- Air sampling should be interrupted during lunch breaks and pumps should be switched off and removed from the worker during this time. If possible, flow rates should be checked during lunch breaks for IOM and cyclone samplers, being adjusted as necessary.

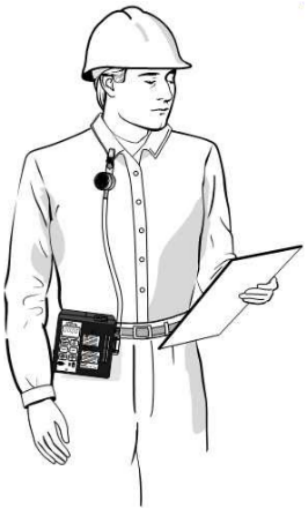

Figure 6. IOM sampler and pump on worker

(© Copyright 2018 SKC Inc.)

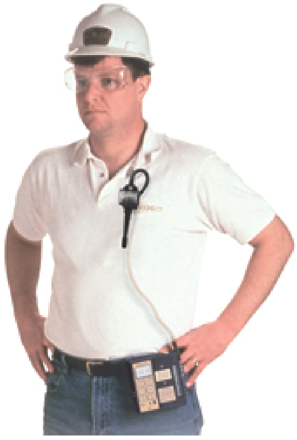

Figure 7. Cyclone sampler and pump on worker (© Copyright 2018 SKC Inc)

At end of sampling period

- Measure and record the flow rate several times (e.g. 6 replicate readings from the calibrated flow meter) before switching off the pump.
- Switch off the pumps and record the time, using a calibrated timepiece.
- Carefully disconnect the samplers from the tubing, without subjecting it to mechanical shocks.
- Cyclones must be always retained upright to avoid contents of the grit pot falling onto the filter
- Remove the IOM cassette from the IOM sampler and attach the protective cap on the IOM cassette and fasten with transport clip. Alternatively, it may be practical to cap the IOM sampler and return to the laboratory for disassembly
- Remove cyclone cassette and fit the sealing clip over the cassette.
- Calculate the average flow rate at the beginning and at the end of the measurement as well as the corresponding relative standard deviation (RSD). If the two flow rates differ by more than 5% or if a RSD value is higher than 2.5%, consider the air sample as invalid.
- Calculate the sampled air volume by multiplying the average flow rate with the sampling duration, for example.
  08:30 IOM sampler placed on worker and started, flow rate = 2000 ml/min
  09:45 flow rate check rate, flow = 1900 ml/min (flow rate reset to 2000 ml/min)
  12:00 Sample off and stopped for lunch (flow rate = 2000 ml/min)
  12:45 lunch break over, sample back on and restarted (flow rate = 2000ml/min)
  13:45 End of sample, sampler stopped (flow rate = 1950ml/min)

Sample period 1; 08:30 – 09:45 (75 mins); average flow rate=(2000+1900)/2 = 1950
Sample period 2; 09:45 – 12:00 (135 mins); average flow rate=(2000+2000)/2 = 2000
Sample period 3; 12:45 – 13:45 (60 mins); average flow rate=(2000+1950)/2 = 1975

Total sample volume = (75 x 1950) + (135 x 2000) + (60 x 1975) = 534750 ml = 534.75 litres

- For each air sample, a sample record sheet should be completed (Appendix 1). Following items are recorded: a unique identification code (including country ID, participant ID and sample ID, as explained below), sampling date, pump ID, start and end time, sampling duration (min), flow rate (L/min) before and after air sampling, sampled air volume (L) and other relevant sampling information (location, activities).

The 8-h TWA for the workers will be calculated at the data analysis stage, using the information provided in the sample record form. It is therefore important that this is fully completed.

Cleaning the IOM and Cyclone Sampler

- Disassemble the IOM and Cyclone Sampler.
- Place parts in an ultrasonic cleaner with water and a wetting agent such as a mild soap. IOM and Cyclone components may also be cleaned with a solvent such as isopropyl alcohol. O-rings should be cleaned separately using water only.
- Clean the components using a clean lint-free paper, cloth, or soft brush. Allow components to dry completely.

Sample traceability and contextual information

A standardised convention will be used to assign unique identification codes for all samples collected. The identification code convention is as follows:

E-waste (E) - Country ID (XX) - Company ID (XX) - Participant ID (XXX) - Sample ID (AX/BX/LCX/RCX/HX/SDX/UX/WX/WBX)

'E' is to denote that the samples and data relate to the e-waste occupational study.

- Country ID 'XX' is the country code, using the ISO Alpha-2 country codes for the participating countries (http://www.nationsonline.org/oneworld/country_code_list.htm).

Country	ISO Alpha-2 country codes
Belgium	BE
Finland	FI
Germany	DE
Latvia	LV
Luxembourg	LU
Poland	PL
Portugal	PT
The Netherlands	NL
United Kingdom	UK

Company ID ‘XX’ is a two-digit running number of companies in each country (e.g. 01 for the first company recruited, 02 the second and so forth).

Participant ID 'XXX' is a three-digit running number of participants in each country (e.g. 001 for the first participant recruited, 002 the second and so forth).

Sample ID ‘AX’ where A denotes the type of sample collected (air), followed by an one-digit identifier (X) to identify the running number of each type of sample for that worker (e.g. 1 for the first sample, 2 for the second and so forth).

The following scenario is provided to illustrate the application of this convention. A worker is recruited in Luxembourg. He is working in the first company recruited. He is the first worker recruited in that company and is providing a first air sample. The sample identification code assigned is therefore E-LU-01-001-A1

Storage of collected air samples

After sampling, air samples should be stored in the original cassettes (with transport or sealing clip). No cooled storage is required. Details of the time period for which the samples are stored before transportation to the lab, should be held.

Quality control

To check for possible contamination during the sampling procedure, transportation and storage, field blanks should be collected for each sampling survey. Submit at least one blank (filter, as well as cassette and cap) for every daily series of inhalable and respirable fraction samples respectively or alternatively for every set of more than 10 inhalable and respirable fraction samples. This blank should be handled in exactly the same way as the sampled filter containing cassette, but with no air drawn. The average mass found in the field blank should be subtracted from the corresponding mass found in the samples.

There are various factors that may affect the validity of the collected aerosol sample, such as: presence of projectile particles entering the sampler, large particles entering the sampler that are outside the inhalable definition, transportation losses (e.g. particles falling off the filter) and sample losses (e.g. wall losses onto the internal walls of the IOM cassette).

In some instances where the aerosol concentrations are unusually variable or there are significant projectile particles present, it is reasonable to assume that the sampler may be unrepresentative of the personal exposure. This should be noted during the sampling and either disregard the result, or treat it as a ‘worst-case’ estimate of personal exposure. If projectile particles are present, then an unpumped sampler positioned next to the pumped sampler may be used to correct for unaspirated particles.

Possible losses on the internal walls of an IOM cassette should be also taken into account. The analyzing laboratory should perform wiping of the inside of the IOM cassette. Moreover, be-

cause wall deposition may not only result from air sampling, but may also occur upon shipment of sampled filters. The inside of the IOM cassette should be swabbed with a fresh filter of the same type as used for the air sampling.

Transportation of air samples to laboratory

After sampling, the labelled filter cassettes, accompanied by the sample record sheets, should be transported to the agreed analytical laboratory, who provided the assembled IOM cassettes and cyclone cassettes. The cassettes should be shipped with the top part directed upwards all the time.

Sample shipment should be ideally the same day as the sampling, however if this is not possible no later than the next day. Details of the numbers of samples being sent, sample identification codes, requested analysis and contact details of the responsible researcher should accompany the samples.

It is recommended that a hard copy of this information be included with the samples and that an electronic version is issued to the receiving laboratory at the time of sending the samples. This will allow sample numbers and identification codes to be checked upon receipt at the laboratory.

Data reporting

It is important that information entered into the sample record sheet (Appendix 1) is recorded as per the instructions given in this SOP as this information will need to be entered into the data template for the overall e-waste study.

With respect to the air samples results, the following information should be obtained from the laboratory undertaking the analysis which is to be entered into the template:

- Air concentration (this must be in μg)
    - If concentration is below the limit of quantification (LOQ), the result is replaced by **<**LOQ (for example, <0.23 if 0.23 is the LOQ). Data below LOQ should not be given as an empty cell, zero concentration or free text (i.e. <LOQ, not detected, n.d., LOQ/2)
      - please ensure that the **<** is used to identify the result as <LOQ.
- Air volume (this must be in m^3)
- Analytical method used
- LOQ of the analytical method (this must be in μg)
- Method to calculate the LOQ (the laboratory should test that the reported LOQ concentration can be analysed accurately (RSD <20 %))

Care must be taken to follow the instructions accompanying the data template. The template must NOT be modified in any way - DO NOT add / remove columns, or alter the drop down lists, or merge cells. In the event that the template is modified or data has been provided which does not follow the instructions, templates will be returned to the data provider for correction.

References

- HSE (2014) MDHS 14/4. General methods for sampling and gravimetric analysis of respirable, thoracic and inhalable aerosols.
  Available from http://www.hse.gov.uk/pubns/mdhs/pdfs/mdhs14-4.pdf
- NIOSH (2014). NIOSH Method 7302 'ELEMENTS by ICP (Microwave Digestion).
  Available from https://www.cdc.gov/niosh/docs/2003-154/pdfs/7302.pdf
- OSHA (1998). OSHA Method ID-215 ‘Hexavalent chromium in workplace atmospheres‘
  Available from https://www.osha.gov/dts/sltc/methods/archive/id215/id215.pdf
- OSHA (2002). OHSA Method ID-125G ‘Metal and metalloid particulates in workplace atmospheres (ICP analysis)’.
  Available from https://www.osha.gov/dts/sltc/methods/inorganic/id125g/id125g.html
  Brief overview available on http://amcaw.ifa.dguv.de/substance/sheets/116-01-S-Chromium%20hexavalent.pdf
- IOM Sampler Instructions and O-Ring Fitting Instructions.
  Available from https://www.skcltd.com/products2/sampling-heads/iom-sampler.html#documentation ; https://www.skcltd.com/images/pdfs/007-05-003.pdf ; https://www.skcltd.com/images/pdfs/007-05-004.pdf
- SKC (2018) Cyclone Samplers for Respirable Dust - Operating Instructions.
  Available from https://www.skcltd.com/images/pdfs/007-04-001_Issue_F_Cyclone_Sampler_Instructions.pdf
- Porras S, Ladeira C, Ribeiro E, Viegas S, Uuksulainen S, Santonen T, Galea K, Cherrie J, Louro H, Ventura C, Silva MJ, Leese E, Jones K, Hanser O, Ndaw S, Robert A, Duca R-C, Poels K, Godderis L, Kiilunen M, Norppa H, Veijalainen H, Parshintsev E, Tuomi T, Ruggieri F, Alimonti A, Koch H, Bousoumah R, Antoine G, Jacoby N, Musgrove D. (2019) HBM4EU occupational biomonitoring study on hexavalent chromium and other harmful chemicals. Standard Operating Procedures (SOPs). WP8 - Targeted field work surveys and alignment at EU level. Available from URL: https://www.hbm4eu.eu/online-library/ (last accessed 22nd February 2021).

Appendix 1: Sample record sheet

**Identification code:**		**Company Name:**	
**Sampling date:**		**Aerosol fraction:**	Inhalable / respirable
**Sampling head used:**		**Filter media used:**	
**Shipping date:**		**Company ID:**	
**Researcher:**		**Participant Name:**	
**Organisation:**		**Participant ID:**	
**Duration of worker shift (mins)**		**Were samples left on and running during worker breaks? If so, what was the duration of this sampled break time**	Yes / No<br>.......................... mins
**Duration of worker breaks during shift (mins)**			

**Pump ID**	**Start time (00:00)**	**End time (00:00)**	**Sampling duration (min)**	**Flow rate (L/min) *before***	**Flow rate (L/min) *after***	**Average flow rate (L/min)**	**Air volume (L)**	**Location of the sampler (left or right side)**	**Activity**
**Period 1**									
**Period 2**<br>**(if required)**									
		**Overall sample duration:**			**Overall average flow rate:**				

Supplementary material S5.7

SOP-7 Obtaining hair samples

Authors and Acknowledgements

Lead authors:

This document has been developed by Emilie Hardy (LNS, Luxembourg) and Radu Corneliu Duca (LNS).

Contributions were received from: Karen Galea (IOM, UK) and Adrian Covaci (UAntwerpen, Belgium)

This document has been created for the HBM4EU project. HBM4EU has received funding from the European Union's Horizon 2020 research and innovation programme under grant agreement No 733032.

Introduction

The purpose of this Standard Operating Procedure (SOP) is to establish a standardised procedure for collecting head hair samples for the assessment of exposure in the e-waste workplace biomonitoring study.

The collection and analysis of this biological matrix will provide information on the level of occupational exposure to metals (and some organic compounds) for e-waste workers. Indeed, hair analysis has become a very useful tool for human biomonitoring [1, 2, 3] since this matrix reflects the level of exposure during a wider time window than urine or blood [4]. Hair analysis offers the potential for evaluation of chronic exposure during several months (depending on the length of the hair strand analysed), while blood and urine represents only recent exposure (from a few hours to several days). Since the hair growth is considered to be 1 cm/month [4,5], segmentation of the hair sample could also provide a detailed history of the exposure for the last months before sampling [4].

This SOP is based on the SOP for scalp hair sampling already employed for the COPHES project [6] and the Society of Hair Testing (SoHT) guidelines for drug testing in hair [4].

The hair samples collected are to be analysed for metals by the Laboratoire National de Santé (LNS, Luxembourg) to investigate the suitability of hair matrix in occupational health studies. Depending on the amount of sample collected and, on the feasibility, the University of Antwerpen (UA) may also determine some organic compounds such as brominated flame retardants and PCBs.

Materials required for the collection of hair samples

To ensure a uniform collection of hair samples for the whole study, researchers should ensure that they have the following materials:

- Powder-free disposable nitrile gloves
- Titanium scissors (at least with undamaged titanium blade coating)
- Alcohol solution prepared using 70% of isopropanol and 30% of deionised water (v/v)
- Paper tissues (or cotton)
- Ruler or measuring tape
- Hair clip
- Kitchen twine or surgical rope
- Permanent marker
- Small paper envelopes (90x140 mm) and cardboard sheets (105x218 mm)
- Sample labels
- Ziplock plastic bags
- Sample record sheets (Appendix 1): one form per sample
- Pens
- A couple of mirrors

Information to communicate to the participants

Participants should be fully informed of the sampling procedure. They will be advised that the sampling has to be done before the work shift and preferably before the workweek in order to

limit external contamination of the hair shaft. They will also be informed in advance that one or a few strands of hair will be collected by cutting as close to the scalp as possible.

Researcher precautions

When collecting each hair sample from the participants, researchers must always wear a new pair of powder-free disposable nitrile gloves.

To avoid cross-contamination between samples and to ensure each participant safety, the scissors and the ruler used for the samplings have to be cleaned/ disinfected with alcoholic solution before and after each sampling and the collection has to be done in an area considered to be free of potential contamination (as far as possible). Since chromium element has to be determined in the collected hair samples, direct contact of hair fibers with stainless steel should be avoided so the scissors material has to be carefully selected and checked (full titanium or undamaged titanium-coated blades).

Researchers should also ensure to collect a sufficient amount for the analysis, i.e. a mass of minimum 500 mg of hair (section diameter of the strand: 1 cm almost equivalent to a big pencil/marker thickness). To avoid "invasive" sampling, i.e. a more aesthetic aspect for the participants, it is possible to combine different small strands cut in the posterior vertex region (see picture below).

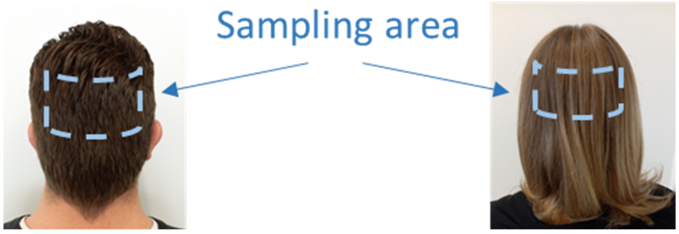

In the case of hair samples longer than 6 cm, it is also particularly important to keep the hair fibers aligned with the root end clearly identified and secured, as far as possible.

Sampling procedures

Participants must be seated in order to facilitate and secure the hair sampling.

Before each sampling, put on a pair of powder-free disposable nitrile gloves and clean/disinfect properly the scissors with a paper tissue moistened with alcohol solution. Measure the length of the hair of the back of the head using a ruler or a measuring tape cleaned/disinfected using the procedure previously mentioned.

After that, two different procedures have to be applied depending on the hair length:

A. Hair shorter than or equal to 6 cm (2.36 inch)

Cut 15-20 strands of hair as close as possible to the scalp from different places on the middle of the back of the head and put them directly on the cardboard sheet. The minimum mass of

sample required is 500 mg (equivalent to 18 strands like the ones presented on the picture (see below).

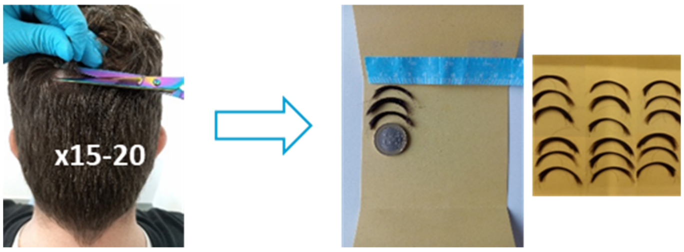

Fold the cardboard sheet in three and place it inside the paper envelope. Label the envelope with the sample identification code and place the envelope in a plastic bag (see pictures below).

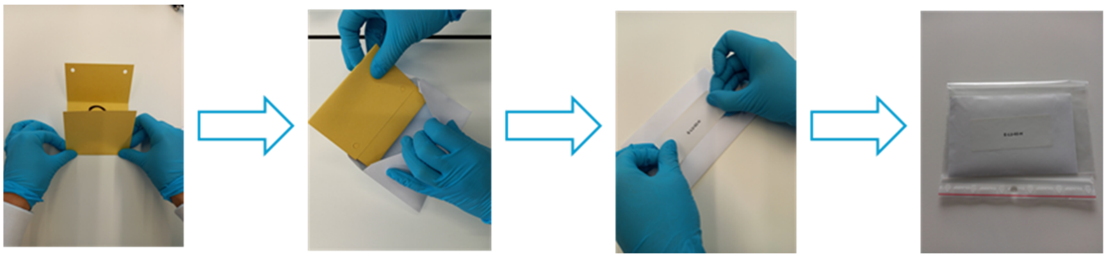

B. Hair longer than 6 cm (2.36 inch)

Grasp and lift the hair to access the middle of the back of the head. A hair clip may be helpful for this.

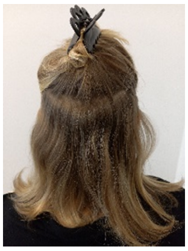

Select a lock of hair and fasten it with the string at 1-2 cm to the scalp. Cut it as close as possible to the scalp.

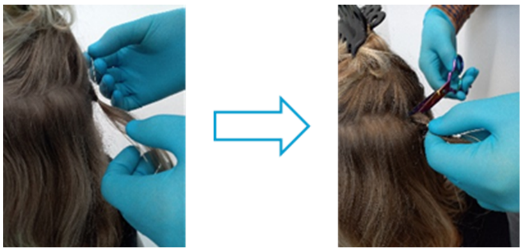

Place it on the carboard sheet and identify the root end on the sheet. Repeat until obtaining the desired amount of sample and add the strands on the sheet (for this size of lock, two strands are necessary).

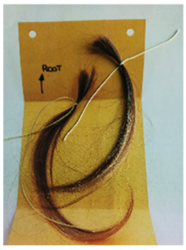

Fold the sheet to secure the strands and keep them well aligned until analysis in the agreed analytical laboratory.

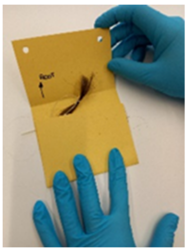

Place the cardboard sheet in the paper envelope, which you will label with the sample identification code.

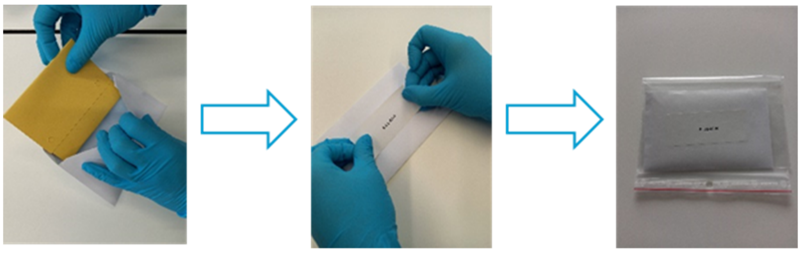

After each sampling, clean/disinfect the scissors and the ruler, dispose of the gloves and fill in the sample record sheet with the participant (Appendix 1). The researcher may also suggest the participant to look in a mirror how much was cut to be reassured.

Sample traceability and contextual information

A standardised convention will be used to assign unique identification codes for all samples collected. The identification code convention is as follows:

E-waste (E) - Country ID (XX) - Company ID (XX) - Participant ID (XXX) - Sample ID (AX/BX/LCX/RCX/HX/SDX/UX/WX/WBX)

'E' is to denote that the samples and data relate to the e-waste occupational study.

Country ID 'XX' is the country code, using the ISO Alpha-2 country codes for the participating countries (http://www.nationsonline.org/oneworld/country_code_list.htm).

Country	ISO Alpha-2 country codes
Belgium	BE
Finland	FI
Germany	DE
Latvia	LV
Luxembourg	LU
Poland	PL
Portugal	PT
The Netherlands	NL
United Kingdom	UK

Company ID ‘XX’ is a two-digit running number of companies in each country (e.g. 01 for the first company recruited, 02 the second and so forth).

Participant ID 'XXX' is a three-digit running number of participants in each country (e.g. 001 for the first participant recruited, 002 the second and so forth).

Sample ID ‘HX’ where H denotes the type of sample collected (hair), followed by an one-digit identifier (X) to identify the running number of each type of sample for that worker (e.g. 1 for the first sample, 2 for the second and so forth).

The following scenario is provided to illustrate the application of this convention. A worker is recruited in Luxembourg. He is working in the first company recruited. He is the first worker recruited in that company and is providing a first hair sample. The sample identification code assigned is therefore E-LU-01-001-H1

The unique sample identification code has to be clearly written on the sample envelope. A sample record sheet and questionnaire with the same identification code should be completed when collecting the hair sample (Appendix 1).

Storage of collected samples

After the collection and before the shipping to the concerned laboratories, the envelopes containing the collected hair samples have to be stored at room temperature in a dry and dark environment, so preferably in a non-transparent cardboard box.

Transportation of samples to laboratory

Local arrangements will need to be put in place with respect to the transportation of the samples to the laboratory. For example, in some instances the samples may be driven by the researcher to the laboratory whereas in others, courier delivery may be necessary.

Since hair is a particularly stable biological matrix (no degradation or any loss of the incorporated compounds is expected), the samples can be sent to the agreed analytical laboratory once the complete collection necessary for the study is done. Details of the numbers of samples being sent, sample identification codes, requested analysis and contact details of the responsible researcher should accompany the samples.

It is recommended that a hard copy of this information be included with the samples and that an electronic version is issued to the receiving laboratory at the time of sending the samples. This will allow sample numbers and identification codes to be checked upon receipt at the laboratory.

Reporting

It is important that information entered into the sample record sheet (Appendix 1) is recorded as per the instructions given in this SOP as this information will need to be entered into the data template for the overall e-waste study.

Care must be taken to follow the instructions accompanying the data template. The structure, drop down lists etc of the data template must NOT be modified in any way. In the event that the template is modified or data has been provided which does not follow the instructions, templates will be returned to the data provider for correction.

References

[1] Smolders R, Schramm KW, Nickmilder M, Schoeters G. (2009), Applicability of non-invasively collected matrices for human biomonitoring, *Environmental Health*, 8:8.

[2] Appenzeller BMR., Tsatsakis AM. (2012), Hair analysis for biomonitoring of environmental and occupational exposure to organic pollutants: State of the art, critical review and future needs, *Toxicology Letters*, 210 (2) 119-140.

[3] Alves A, Kucharska A, Erratico C, Xu FC, Den Hond E, Koppen G, Vanermen G, Covaci A, Voorspoels S. (2014), Human biomonitoring of emerging pollutants through non-invasive matrices: state of the art and future potential, *Analytical and Bioanalytical Chemistry*, 406 (17) 4063-4088.

[4] Cooper GAA., Kronstrand R, Kintz P. (2012), Society of Hair Testing guidelines for drug testing in hair, *Forensic Science International*, 218 (1-3) 20-24.

[5] Harkey MR. (1993), Anatomy and physiology of hair, *Forensic Science International*, 63 (1-3) 9-18.

[6] COPHES Standard Operating Procedure for Scalp Hair Sampling, D3. 1. Annexes, FP7-244237, 21 March 2011.

Appendix 1: Sample record sheet

HBM4EU
science and policy
for a healthy future

Occupational Biomonitor-
ing
e-Waste Study

HAIR SAMPLE RECORD SHEET

**Company Name:**	
**Company ID:**	
**Worker ID:**	
**Worker name:**	
**Job title:**	

**Sample ID:**	
**Sampling date:**	
**Researcher(s):**	
**Organisation:**	

**Participant authorization and sample obtained:**	⃝ Yes<br>⃝ No → details: ______________________________
**Collection at the beginning of the work-week:**	⃝ Yes<br>⃝ No → details: ______________________________
**Collection before the workshift:**	⃝ Yes<br>⃝ No → details: ______________________________
**Sample length (cm):**	
**Natural color of the hair:**	⃝ Black/dark brown<br>⃝ Brown/brunette<br>⃝ Blond<br>⃝ Red<br>⃝ Grey<br>⃝ White
**Cosmetic treatment applied on hair within the last 6 months:**	⃝ None<br>⃝ Dye/Tinting → ______ months ago<br>⃝ Chemical hair structure treatment (perm or hair straightening) → ______ months ago
**Use of curling or straightening iron:**	⃝ Yes → frequency: ______ times / month<br>⃝ No

Appendix 2: Hair sampling guide

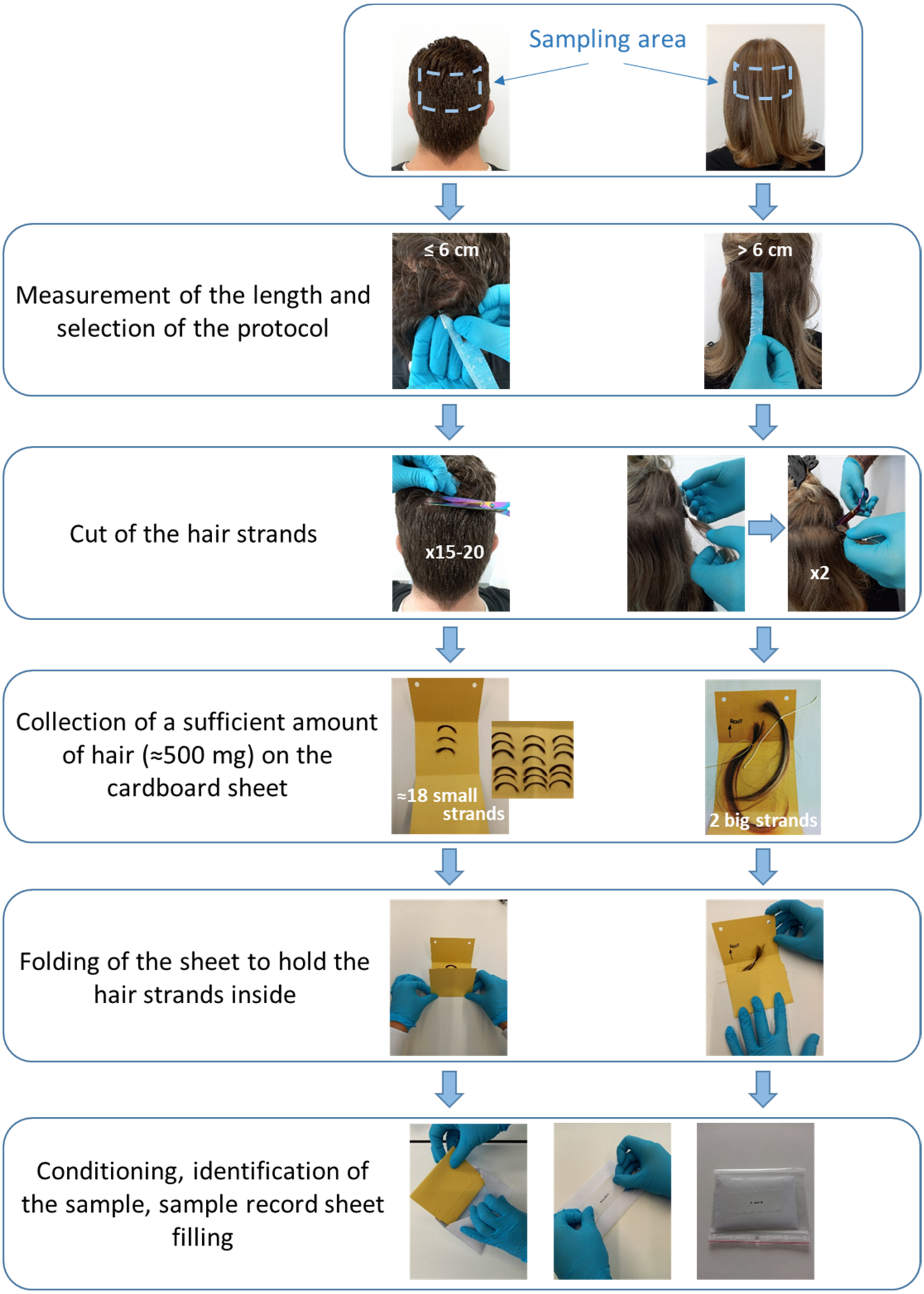

Supplementary material S5.8

SOP-8 Dermal sampling using wipes and wristbands

Authors and Acknowledgements

Lead authors:

This document has been developed by Radu Corneliu Duca (LNS, Luxemburg), Karen Galea (IOM, UK), Paul Scheepers (Radboudumc, the Netherlands)

Contributions were received from:

Martien Graumans and Maurice van Dael (Radboudumc), Tiina Santonen (FIOH, Finland), Kate Jones and Darren Musgrove (HSL, UK) and Ogier Hanser (INRS, France).

This SOP was developed with due consideration of the contents of the "SOP7: Standard operating procedure for obtaining dermal wipe samples" developed for the HBM4EU occupational biomonitoring study on hexavalent chromium and other harmful chemicals (Porras et al., 2019).

This document has been created for the HBM4EU project. HBM4EU has received funding from the European Union's Horizon 2020 research and innovation programme under grant agreement No 733032.

Introduction

The purpose of this Standard Operating Procedure (SOP) is to establish a uniform procedure for collecting wipe and wristband samples for the evaluation of dermal exposure in the e-waste workplace biomonitoring study. The wiping technique is based on the removal of substances from the skin contamination layer by the application of a mechanical, fluid dynamic and/or chemical force to a moist medium that equals or exceeds the force of adhesion. Low-cost silicone wristbands are a non-invasive passive sampling technique that has been used to assess cumulative exposure over several days (e.g. Aerts et al, 2018; Wong S et al., 2019).

The collection and assessment of these occupational hygiene samples will provide information on the level of contamination experienced on the hands and can be used to evaluate protective glove effectiveness and workplace sanitation procedures. Average hand areas will be used in subsequent calculations, these being:

- 535 cm^2 per male hand, 445 cm^2 per female hand (US EPA, 2011).

The SOP is based on internal SOPs employed by IOM to assess dermal exposure to various metals such as zinc and nickel as well as crude oil / base oils using wipe sampling methods, and the protocol presented by Aerts et al, 2018 Environ. Sci. Technol. 2018, 52, 298−307 for the use of wristband passive samplers, respectively. It involves the use of SKC Ghost sampling wipes, which can also be used to collect samples of several metals including Cr, Cd, Pb and Hg, as specified in OSHA Method ID-125G, Addendum B[4] and of Silicon wristbands, which can be used to collect samples of different organic compounds including flame retardants. It should however be noted that recovery, sampling and storage efficiency of the wipe and wristbands sampling method has not been validated within the HBM4EU project.

The goal is to provide a uniform methodology to collect representative samples to determine the study participants' dermal exposure in a standardized manner.

The Ghost wipes are to be analysed for metals using OHSA Method ID125G ‘Metal and metalloid particulates in workplace atmospheres (ICP analysis)’ by laboratories experienced in this analysis.

The Silicon wristbands are to be analysed for flame retardants by laboratories experienced in this analysis, in accordance for instance with the protocol proposed by Wong S et al., 2019.

Materials required for the collection of wipe samples

To undertake the collection of wipe samples, researchers should ensure that they have sufficient quantities of the following:

- Ghost wipes, moistened with deionised water (individually packed). Part No: 225-2414 (200 wipes); part No: 225-2413 (1,000 wipes)
- Silicon wristbands, adult-size (202 mm L × 12 mm W × 2 mm T; weight 5.33 g, SD = 0.10 g) with HBM4EU debossed text (e.g. https://www.promofit.nl/promo-premiums/polsbandjes-bedrukken/siliconen-polsbandjes-bedrukken/

[4] http://www.skcltd.com/products2/9-uncategorised/310-surface-and-skin-1

- The wristbands will be distributed uncleaned. So prior to utilization the wristbands need to be cleaned (30 min with 1:1 ethyl acetate/hexane (v/v) (125ml of each for n=5 wristbands) followed by a second 30 min cleaning with 1:1 ethyl acetate/methanol (v/v) (125ml of each for n=5 wristbands) in an overhead shaker and subsequently dried under N_2 at 40°C (consider to be modified to a higher temperature). Each wristband will be stored in aluminium/LDPE zip lock bags (see Figure 3) at -20°C until further use. Keep 5 cleaned wristbands as blanks to be returned with the field samples for the laboratory to verify the background).

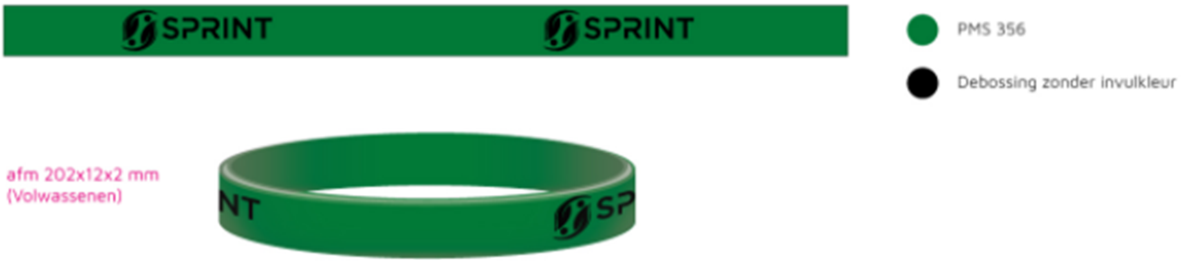

Figure 1: Wristband. The colour will be changed to yellow and the text to *'HBM4EU'*.

- Digestion tubes with screw caps (sufficient number for the wipe samples).
- Aluminium/LDPE ziplock bags for wristband storage
- Sample labels
- Supply of powderless disposal nitrile gloves
- Sample record sheets (Appendix 1)
- Supply of disposable paper towels
- Pens

Participant instructions

Participants should be fully informed of the sampling procedure. They will be advised of the following points before commencing their work shift/work week:

a. For wipe sample collection

- Rings should be removed (wherever possible) and stored securely.
- Participants will be asked to thoroughly wash and dry their hands with soap and water before commencing their work shift. Hands will be dried using fresh disposable paper towels (which can be provided as necessary) or hand dryers. Reusable fabric towels must not be used.
- A wipe sample will then be collected from the dominant hand, prior to the participant commencing their work activity using the standardised wiping procedure.
- Further wipe samples will be collected from the participants dominant hand prior to their refreshment/comfort and lunch and before they finish their work shift. Participants must not wash their hands before the wipe samples are collected.
- The participant should also be informed that if for any reason they do wash their hands between any two wipe sampling periods that they should inform the researcher that this is the case.

b. For wristband samples collection:

- Wristbands will be worn for the entire work-week period (starting on the 1st morning of the working week) only during working activities.
- From the end of each working day to the following work morning wristbands must be placed in their original zip lock bags and stored overnight in a clean area.
- At the end of working week (end of the working day), wristbands must be placed in their original ziplock bags for transport to the lab. Keep cool during transportation and stored at -20°C until shipment.

Researcher precautions

When collecting each wipe or wristband sample from the participants, researchers must always wear a new pair of unpowdered disposable nitrile gloves.

Care should also be taken to avoid cross contamination of samples. It is recommended that where possible the wipes or wristbands are collected from the participants in an area considered to be free of potential contamination e.g. office space, medical room etc. Wipes or wristbands should not be collected from participants in the physical work area where, for example, where e-waste dismantling activities are taking place due to the risk of cross contamination. Appropriate quality assurance procedures must be applied to check this is the case (See Section 9).

Researchers collecting the wipe samples should try to ensure that a consistent amount of pressure is applied when wiping the participants hands. It is recommended that the number of researchers collecting the samples is minimised to one or two per country where possible.

Researchers should ensure that a site-specific risk assessment of their work practices is undertaken prior to commencing the measurement campaign and that all necessary health and safety precautions are adopted and followed.

Sampling procedures

Ensure have a sufficient number of prepared wristbands (one for each participating worker), stored in ziplock bags with a unique number (see Section 7).

Prepare a sufficient number of digestion tubes (for the wipe samples) each labelled with a unique number (see Section 7).

Wear a new pair of clean disposable gloves for each sample. DO NOT use powdered gloves.

Record the sample number and the participant details on the sample record form (Appendix 1).

The following procedures should be used to collect the wipe samples.

- Remove the wipe from its wrapper using gloved hands. Do not use metal tweezers to handle the wipe, as they could contaminate the sample.
- Note on the record sheet if the participant's hands are observed to be wet with water or if participant has washed their hands prior to sampling.

- A standardised wiping technique will be used which involves wiping the whole palmer and dorsal area and fingers of the dominant hand:
    - o Ask the participant what their dominant hand is
    - o The wiping will be done only on the dominant hand, wipe five times the palm of the hand from the top of the hand to the start of the fingers and five times across.
    - o Repeat the procedure to sample the back of the hand.
    - o Fold the wipe in half (with the contaminant side inward) and sample the fingers trying to wipe well in between the fingers (Figure 2).
    - o Wipe twice the palm of each finger, from the top to the fingertip. Repeat, to sample the back of the fingers.

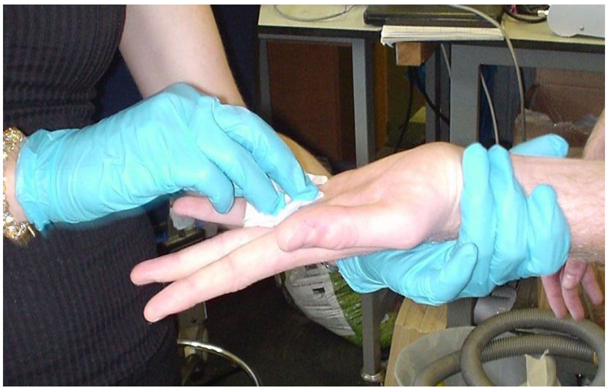

134 **Figure 2:** Dermal sample collected from the fingers

- After wiping, fold the wipe again with the contaminant side inward. Place the wipe immediately in the labelled digestion tube and securely seal using the screw cap.
- Submit one blank wipe for every ten hand wipe samples, treated in the same fashion, but without wiping (See Section 9).

The following procedures should be used to collect the wristband samples

- Remove the pre-cleaned wristband from its zip lock bag (Fig. 3). The wristband is to be worn on the wrist of the dominant hand and must be worn for the entire working-week (starting on the 1st morning of the working week) only during the working hours.
- From the end of each working day to the following work morning wristbands must be placed in their original zip lock bags and stored overnight in a clean area.
- Upon the end of the sampling work-week period the wristbands will be placed in the original zip lock bag and returned to the researcher.
- Submit one blank wristband for every ten wristbands worn at the site. These should be stored in their zip lock bag for the entire working week (See Section 9).

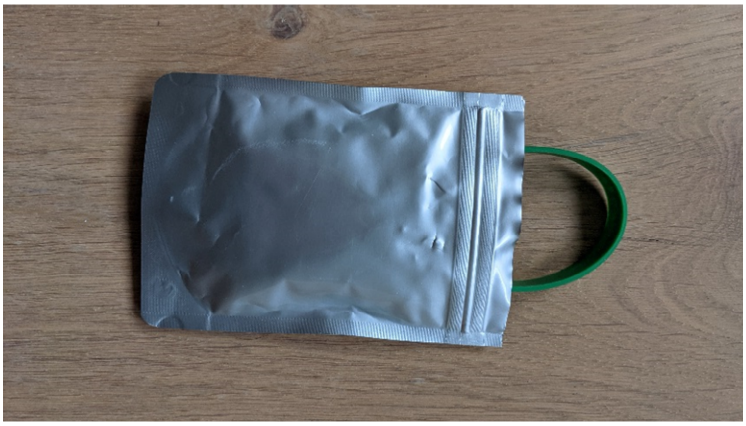

Figure 3: Wristband with zip lock bag

Frequency of sample collection

The number of wipe samples collected will be dependent on the shift duration and number of rest breaks the participant has. The researchers should use their judgment to decide on the numbers of samples to be collected. For example, if the participant works a full shift on e-waste activities and has two rest breaks and one lunch break, there will be five sample collection periods: pre-shift, first break period, lunch, second break period and post-shift, resulting in 5 samples being collected per participant.

A single wristband sample per worker will be taken for the entire work-week period.

Sample traceability and contextual information

A standardised convention will be used to assign unique identification codes for all samples collected. The identification code convention is as follows:

E-waste (E) - Country ID (XX) - Company ID (XX) - Participant ID (XXX) - Sample ID (AX/BX/LCX/RCX/HX/SDX/UX/WX/WBX)

'E' is to denote that the samples and data relate to the e-waste occupational study.

Country ID 'XX' is the country code, using the ISO Alpha-2 country codes for the participating countries (http://www.nationsonline.org/oneworld/country_code_list.htm).

Country	ISO Alpha-2 country codes
Belgium	BE
Finland	FI
Germany	DE
Latvia	LV
Luxembourg	LU
Poland	PL
Portugal	PT

The Netherlands	NL
United Kingdom	UK

Company ID 'XX' is a two-digit running number of companies in each country (e.g. 01 for the first company recruited, 02 the second and so forth).

Participant ID 'XXX' is a three-digit running number of participants in each country (e.g. 001 for the first participant recruited, 002 the second and so forth).

Sample ID. 'WX' denotes the collection of a wipe sample, followed by an one-digit identifier (X) to identify the running number of each type of sample for that worker (e.g. 1 for the first sample, 2 for the second and so forth). 'WB' denotes the collection of a wristband sample.

The following scenario is provided to illustrate the application of this convention. A worker is recruited in Luxembourg. He is working in the first company recruited. He is the first worker recruited in that company and is providing a first wipe sample. The sample identification code assigned is therefore E-LU-01-001-W1. The code for this workers wristband sample is E-LU-01-001-WB1.

The unique sample identification code will be clearly stated on the labelled digestion tube / wristband vial. A sample record sheet should be completed when collecting the wipe samples (Appendix 1).

Storage of collected samples

The collected wipe samples can be stored at room temperature in a clean box before transportation to the laboratory. Since no standard recommendations are available for wristbands, a stricter approach is recommended for their storage after the sampling period. Thus, the collected wristband samples can be kept preferably cool (or at room temperature but not more than 24h). If longer storage period is required the samples should be kept at -20°C prior to the transport to laboratory as well as in the lab prior to analysis.

Quality control

To check for contamination during the sampling procedure, transportation and storage, field blanks should be collected for each sampling survey.

These samples should be treated in the same way as the exposure samples, using the same procedures as previously described. The average mass found in the field blank should be subtracted from the corresponding mass found in the samples. In the event of elevated concentrations being observed in the blank samples these will be investigated and used to flag any suspect participant wipe samples.

The number of field blanks should be no less than 10% of the number of dermal samples. It is recommended that at least one wipe blank is collected, with further wipe blanks to be collected for every 10 wipe samples obtained. This recommendation also applies to the collection of blank wristband samples.

Transportation of samples to laboratory

Local arrangements will need to be put in place with respect to the transportation of the samples to the laboratory. For example, in some instances the samples may be driven by the researcher to the laboratory whereas in others, courier delivery may be necessary.

Samples should be transported to the agreed analytical laboratory ideally the same day however if this is not possible no later than the next day. Details of the numbers of samples being sent, sample identification codes, requested analysis and contact details of the responsible researcher should accompany the samples.

It is recommended that a hard copy of this information be included with the samples and that an electronic version is issued to the receiving laboratory at the time of sending the samples. This will allow sample numbers and identification codes to be checked upon receipt at the laboratory.

Whilst no storage stability tests have been established within the HBM4EU project it is recommended that all collected samples are analysed as soon as possible by the receiving laboratory and certainly within no more than 14 days of collection.

Reporting

It is important that information entered into the sample record sheet (Appendix 1) is recorded as per the instructions given in this SOP as this information will need to be entered into the data template for the overall e-waste study.

Care must be taken to follow the instructions accompanying the data template. The structure, drop down lists etc of the data template must NOT be modified in any way. In the event that the template is modified or data has been provided which does not follow the instructions, templates will be returned to the data provider for correction.

References

Aerts R, Joly L., Szternfeld P, Tsilikas K, De Cremer K, Castelain P, Aerts JM, Van Orshoven J, Somers B, Hendrickx M, Andjelkovic M, Van Nieuwenhuyse A. (2018). Silicone Wristband Passive Samplers Yield Highly Individualized Pesticide Residue Exposure Profiles. *Environmental Science & Technology*, *52*(1), 298–307.

Wang S, Romanak KA, Stubbings WA, Arrandale VH, Hendryx M, Diamond ML, Salamova A, Venier M. (2019) Silicone wristbands integrate dermal and inhalation exposures to semi-volatile organic compounds (SVOCs). *Environment International*, 132:105104.

OHSA Method ID125G 'Metal and metalloid particulates in workplace atmospheres (ICP analysis)'. URL: https://www.osha.gov/dts/sltc/methods/inorganic/id125g/id125g.pdf (last accessed: 22 February 2021).

Porras S, Ladeira C, Ribeiro E, Viegas S, Uuksulainen S, Santonen T, Galea K, Cherrie J, Louro H, Ventura C, Silva MJ, Leese E, Jones K, Hanser O, Ndaw S, Robert A, Duca R-C, Poels K, Godderis L, Kiilunen M, Norppa H, Veijalainen H, Parshintsev E, Tuomi T, Ruggieri F, Alimonti A, Koch H, Bousoumah R, Antoine G, Jacoby N, Musgrove D. (2019) HBM4EU occupational biomonitoring study on hexavalent chromium and other harmful chemicals. Standard Operating Procedures (SOPs). WP8 - Targeted field work surveys and alignment at EU level. Available from URL: https://www.hbm4eu.eu/online-library/ (last accessed: 22 February 2021).

US EPA (2011). Exposure Factors Handbook 2011 Edition (Final Report). U.S. Environmental Protection Agency, Washington, DC, EPA/600/R-09/052F, 2011. URL: https://cfpub.epa.gov/ncea/risk/recordisplay.cfm?deid=236252 (last accessed: 22 February 2021).

Appendix 1: Sample record sheet

**Company Name:**	
**Company ID:**	
**Worker ID:**	
**Name (family name, initial):**	
**Job title:**	
**Dominant hand of worker:**	

**Researcher(s):**	
**Organisation:**	
**Date sampling:**	

Wipe Sample collection

**Collection round**	**Sample ID**	**Time collected (00:00)**	**Gloves worn prior to collection (Y/N)**	**Type gloves worn**	**Hands washed prior to collection (Y/N)**	**Hands observed to be wet (Y/N)**
Pre-shift						
Break 1						
Lunch						
Break 2						
End shift						

Blank						

Wristband sampler

Sample ID	Placed on dominant hand? (Y/N)	Date wrist-band donned (dd:mm:yyy)	Time donned (00:00)	Day wristband collected (dd:mm:yyyy)	Time collected (00:00)

Questions for the participant when collecting the wristband

Did you encounter any problems or any deviations from the wearing instruction for the wristbands over the work-week?	Yes / No
If yes, please provide more detail.	Reason:

Supplementary material S5.9

SOP-9 Buccal cells sampling including sample storage and transfer

Authors and Acknowledgements

Lead authors

Henriqueta Louro and Maria João Silva, National Institute of Health Dr. Ricardo Jorge (INSA), Lisbon, Portugal

Contributions were received from: Ana Tavares (INSA), Julia Catalán, Kukka Aimonen, Hannu Norppa (FIOH, Finland) and Maurice van Dael (Radboudumc, the Netherlands).

The text has been partially adapted for the HBM4EU occupational chromate study based on the document SOP 3: Procedure for obtaining human samples, prepared under WP 7, Task 7.2, D 7.3.

This document has been created for the HBM4EU project. HBM4EU has received funding from the European Union's Horizon 2020 research and innovation programme under grant agreement No 733032.

Introduction

Genomic Instability has been observed in workers involved in processing electronic waste (Wang et al, 2018; Li et al., 2014; Liu et al., 2009).

The buccal micronucleus assay is a minimally invasive approach for measuring DNA damage, cell proliferation, cell differentiation and cell death in exfoliated buccal cells (Bolognesi et al., 2015). It offers a great opportunity to evaluate in a clear and precise way the appearance of genetic damage whether it is present as a consequence of occupational or environmental risk, being reliable, fast, relatively simple, cheap, and minimally invasive and causes no pain (Torres-Bugarín et al., 2014). Previous studies suggested that this effect biomarker can be related to waste exposure. One work reported micronuclei and other nuclear anomalies in exfoliated buccal cells from urban solid waste collectors and recyclers in southern Brazil (Brina et al 2017), and other works indicated higher frequencies of buccal micronuclei and other nuclear abnormalities such as karyolytic and karyorrhectic cells in waste pickers women (Franco de Diana et al., 2018). More importantly, increased frequencies of micronucleus, karyolysis, and pycnosis in the exfoliated buccal cells in scavenging teenagers at Alaba International market has been related to E-waste indiscriminate disposal and primitive recycling processes, possibly due to elevated Serum Pb, Ni, Cd, and Cr Levels (Alabi et al., 2020). Thus, this effect biomarker seems to be suitable to assess local effects from e-waste exposure. On the other hand, studies on workers exposed to diisocyanates utilized in the manufacture of polyurethane foam have reported increased genotoxic effects (Lindberg et al., 2011). Both toluene diisocyanate- and 4,4′-methylenediphenyl diisocyanate- exposed workers showed increased frequencies of micronuclei in peripheral blood lymphocytes (Norppa et al., 2000; Bilan, 2004) and in buccal epithelial cells (Norppa et al,, 2000), confirming the suitability of this assay to assess diisocyanates.

This guideline is intended to be used in the framework of the Human Biomonitoring Initiative (HBM4EU). The Standard Operating Procedure (SOP) for buccal cells sampling provides the general procedure for the collection, storage and transfer of buccal cell samples to be analysed within the diisocyanate and the e-waste occupational studies.

Precautions in the pre-analytical phase

Although quality control measures are often absent from the pre-analytical phase, it is essential to avoid, or at least minimise, sample misidentification and possible sources of contamination. In this regard, two main groups of factors should be considered:

Influencing factors:

> Alcohol consumption, medication intake and smoking or diet, are influencing factors that can modify the levels of MN in buccal cells. This information will be collected through questionnaire (SOP 2) and should be taken into account when analysis the results.

Interfering factors

> Buccal cell samples should be immediately fixed after collection and sent to the Laboratory in fixative medium to avoid cells degradation or morphology alteration. Tubes have to be appropriately coded to avoid misidentification.

Buccal cells Sampling

Sampling schedule

Post-shift collection is preferred.

Buccal cell collection takes approximately 10 minutes per worker.

Sampling material

The following materials and equipment will be necessary for sampling

- Small-headed toothbrushes (2-cm head length) or cytologic brushes are preferential or you can use standardised swabs (e.g.: https://isohelix.com/products/isohelix-dna-buccal-swabs/)
- 30–50 mL polystyrene containers or test tubes labeled LC (left cheek) and RC (right cheek), 2 per worker
- Saccomanno's fixative (50% alcohol which contains approximately 2% of Carbowax 1540), 20 ml per worker

Instructions for buccal cells sampling

The method presented here is described in detail by Bolognesi and Fenech (2019), and is standardized and widely used by others (Thomas and Fenech, 2011; Bolognesi et al., 2013; 2017 Thomas et al., 2009). At the end of this section, an alternative procedure is provided for samples that will be processed up to 24h after collection, without being transported.

1. For each participant prepare two 30-ml polystyrene containers or test tubes, labeled with individual code and LC (left cheek) and RC (right cheek), each containing 10 ml of Saccomanno's fixative.

2. Before buccal cell collection, the mouth of the subject should be rinsed twice thoroughly with 30 ml of water to remove excess debris.

> *! CAUTION Human samples should be considered as infectious and the appropriate safety precautions should be taken.*

3. Gently but firmly rotate a small-headed toothbrush (2-cm head length) 10 times against the inside of the cheek wall in a circular motion starting from the middle and gradually increasing in circumference to produce an outward spiral effect.

> *Use a different toothbrush for each cheek.*
>
> *! CAUTION It is important to remember not to revisit the mouth with the same toothbrush, so as to avoid the introduction of the fixative to the mucosal lining. Use a new toothbrush for resampling.*

4. The head of the brush is then placed into the fixative container and rotated such that the cells are dislodged and released into the suspension.

> *The cell sampling is performed on the inside of both cheeks to maximize cell collection and to obtain an homogeneous cell suspension, avoiding unknown biases that may be caused by sampling one cheek only.*

5. The sampling brush should be discarded as risk waste after sampling.

6. Tightly seal the tops of the fixative containers and cover in parafilm to prevent leakage during transit from the remote collection location to the laboratory.

7. The containers are then returned to the laboratory for analysis by a courier service, and the laboratory should be informed of their shipment and anticipated arrival date, so that they can be processed as soon as possible after receipt.

8. Buccal cell suspension fixed in Saccomanno's solution can be stored at 4º C for months, but need to be washed with buccal buffer and centrifuged one or two times to be rehydrated before proceeding with next steps.

Alternative procedure:

In step 1, use Buccal cell buffer instead of Saccomanno's fixative for collecting the cells.

Buccal cell buffer preparation: 1.6 g of Tris–HCl (0.01 M), 37.2 g of ethylenediaminetetraacetic acid (EDTA) tetra sodium salt (0.1 M), and 1.2 g of NaCl (0.02 M). Weigh and dissolve in 600 mL of Milli-Q water. Make up the volume to 1000 mL. Adjust pH to 7.0 using 5 M HCl and autoclave at 121 ºC for 30 min. The buffer will last for up to 3 months when stored at room temperature.

Proceed with buccal cells washes and cell spreading within 24h (Thomas et al., 2009).

Sample traceability and contextual information

A standardised convention will be used to assign unique identification codes for all samples collected. The identification code convention is as follows:

E-waste (E) / Diisocyanate (D) - Country ID (XX) - Company ID (XX) - Participant ID (XXX) - Sample ID (LCX/RCX)

'E' is to denote that the samples and data relate to the e-waste occupational study.

'D' is to denote that the samples and data relate to the diisocyanate occupational study.

Country ID 'XX' is the country code, using the ISO Alpha-2 country codes for the participating countries (http://www.nationsonline.org/oneworld/country_code_list.htm).

Country	ISO Alpha-2 country codes
Belgium	BE
Finland	FI
France	FR
Germany	DE
Latvia	LV
Luxembourg	LU
Poland	PL
Portugal	PT
The Netherlands	NL
United Kingdom	UK

Company ID 'XX' is a two-digit running number of companies in each country (e.g. 01 for the first company recruited, 02 the second and so forth).

Participant ID 'XXX' is a three-digit running number of participants in each country (e.g. 001 for the first participant recruited, 002 the second and so forth).

Sample ID 'LCX' where LC denotes the type of sample collected (buccal cells, left cheek), followed by an one-digit identifier (X) to identify the running number of each type of sample for that worker (e.g. 1 for the first sample, 2 for the second and so forth).

The following scenario is provided to illustrate the application of this convention. A worker is recruited in Portugal. He is working in the first company recruited for the E-waste study. He is the first worker recruited in that company and is providing first two samples of buccal cells. One sample

from the left cheek (LC) and one from the right cheek (RC). The sample identification codes assigned are therefore:

E-PT-01-001-LC1

E-PT-01-001-RC1

Conservation, transport and storage of the samples

Processing and storage of collected buccal samples at the local laboratory

Buccal cells fixed in Saccomanno's solution can be stored at 4ºC, allowing preservation of the cell suspensions at 4 ºC for months before processing (Bolognesi and Fenech, 2019).

Transportation of the samples to the laboratory

As a general rule, samples should be shipped to the laboratory as soon as possible. During transportation, the storage conditions precluded above should be maintained.

To **ensure samples transportation at +4°C** (max +10°C) ice packs shall be used, placed at the bottom and along the sides of the styrofoam box, making sure, however, that the samples will not freeze.

A shipping date should be arranged between the sample collectors and the laboratory. When arrangements have been finalized, the addressee should be informed of the time and means of transportation.

The deliverable report *D.7.2 "Strategy and SOPs for human sample exchange, including ethical demands"* includes all information related to the proper conservation and transport of the samples in human biomonitoring studies as well as the conditions of storage until the chemical analysis. The recommendations there referred and included in D7.2. should be followed, namely:

- Standard operating procedure for Sample Exchange on a pan-European level to be used in the HBM4EU initiative
- Shipping Category B Biological Substances
- Pro-Forma Invoice
- Sample Transfer Protocol (Manifest)
- Data Transfer Template.

Storage of the samples in the laboratory until analysis.

Once in the laboratory that will perform the analysis, the storage conditions described in the section 4.1. should be maintained, unless other specific procedure exists in the analytical lab. In addition, the slides remaining after testing will be preserved at least up to the end of the project, unless otherwise stated by national rules.

Further procedures are described in each methodology SOP.

References

Alabi OA, Adeoluwa YM, Bakare AA. Elevated Serum Pb, Ni, Cd, and Cr Levels and DNA Damage in Exfoliated Buccal Cells of Teenage Scavengers at a Major Electronic Waste Dumpsite in Lagos, Nigeria. Biol Trace Elem Res. 2020;194(1):24–33. doi:10.1007/s12011-019-01745-z

Bilban M. Mutagenic testing of workers exposed to toluene-diisocyanates during plastics production process, Am. J. Ind. Med. 45 (2004) 468–474.

Bolognesi C, Fenech M. 2019. Micronucleus Cytome Assays in Human Lymphocytes and Buccal Cells. Methods Mol Biol. 2019;2031:147-163. doi: 10.1007/978-1-4939-9646-9_8.

Bolognesi C, Knasmueller S, Nersesyan A, et al. Inter-laboratory consistency and variability in the buccal micronucleus cytome assay depends on biomarker scored and laboratory experience: results from the HUMNxl international inter-laboratory scoring exercise. Mutagenesis. 2017;32(2):257–266. doi:10.1093/mutage/gew047

Bolognesi C, Knasmueller S, Nersesyan A, Thomas P, Fenech M. The HUMNxl scoring criteria for different cell types and nuclear anomalies in the buccal micronucleus cytome assay - an update and expanded photogallery. Mutat Res. 2013;753(2):100–113. doi:10.1016/j.mrrev.2013.07.002

Bolognesi C, Roggieri P, Ropolo M, et al. Buccal micronucleus cytome assay: results of an intra- and inter-laboratory scoring comparison. Mutagenesis. 2015;30(4):545–555. doi:10.1093/mutage/gev017

Brina KR, Carvalho TS, Ardenghi PG, Basso da Silva L. Micronuclei and other nuclear anomalies in exfoliated buccal cells of urban solid waste collectors and recyclers in southern Brazil. Chemosphere. 2018;193:1058–1062. doi:10.1016/j.chemosphere.2017.11.119

Franco de Diana D, Segovia Abreu J, Castiglioni Serafini D, et al. Increased genetic damage found in waste picker women in a landfill in Paraguay measured by comet assay and the micronucleus test. Mutat Res Genet Toxicol Environ Mutagen. 2018;836(Pt B):19–23. doi:10.1016/j.mrgentox.2018.06.011

Li K, Liu S, Yang Q, et al. Genotoxic effects and serum abnormalities in residents of regions proximal to e-waste disposal facilities in Jinghai, China. Ecotoxicol Environ Saf. 2014; 105, 51–58. doi:10.1016/j.ecoenv.2014.03.034

Lindberg HK, Korpi A, Santonen T, Säkkinen K., Järvelä M., Tornaeus J., Ahonen N., Järventaus H., Pasanen A-L., Rosenberg C., Norppa H. Micronuclei, hemoglobin adducts and respiratory tract irritation in mice after inhalation of toluene diisocyanate (TDI) and 4,4'-methylenediphenyl diisocyanate (MDI). Mutat Res. 2011;723(1):1–10. doi:10.1016/j.mrgentox.2011.03.009

Liu Q, Cao J, Li KQ, et al. Chromosomal aberrations and DNA damage in human populations exposed to the processing of electronics waste. Environ Sci Pollut Res Int. 2009;16(3):329–338. doi:10.1007/s11356-008-0087-z

Norppa H., Bernardini S., Wikman H., Järventaus H., Rosenberg C., Bolognesi C., Hirvonen A. Cytogenetic biomarkers in occupational exposure to diisocyanates: influence of genetic polymorphisms of metabolic enzymes, Environ. Mol. Mutagen. 35 (Suppl. 31) (2000) 45.

Thomas P, Fenech M. Buccal micronucleus cytome assay. Methods Mol Biol. 2011;682:235–248. doi:10.1007/978-1-60327-409-8_17

Thomas P, Holland N, Bolognesi C, et al. Buccal micronucleus cytome assay. Nat Protoc. 2009;4(6):825–837. doi:10.1038/nprot.2009.53

Tolbert PE, Shy CM, Allen JW. Micronuclei and other nuclear anomalies in buccal smears: methods development. Mutat Res. 1992;271(1):69–77. doi:10.1016/0165-1161(92)90033-i

Torres-Bugarín O, Zavala-Cerna MG, Nava A, Flores-García A, Ramos-Ibarra ML. Potential uses, limitations, and basic procedures of micronuclei and nuclear abnormalities in buccal cells. Dis Markers. 2014;2014:956835. doi:10.1155/2014/956835.

Vidyalakshmi S, Nirmal RM, Veeravarmal V, Santhadevy A, Aravindhan R, Sumathy. Buccal Micronuclei Assay as a Tool For Biomonitoring DNA Damage in Oral Lichen Planus. J Clin Diagn Res. 2016;10(7):ZC05–ZC7. doi:10.7860/JCDR/2016/17074.8072

Wang Y, Sun X, Fang L, et al. Genomic instability in adult men involved in processing electronic waste in Northern China. Environ Int. 2018;117:69–81. doi:10.1016/j.envint.2018.04.027

Annex 1 – Buccal cells Sampling form

Confidential Data – do not send with sample

Study Identification (Diisocyantes/ E-waste): ______________________________

Worker Identification:

Country: ____

Company name and name of department: ___________________________________

Worker name: ____

Position: ____

Control? __________(Yes/No)   Exposed? __________ (Yes/No)

Date: __________________________

Code Number:

Send with sample for analysis

***Questionnaire*:**

Date: ______________

Code Number: ___________________

Buccal Sample:

Date: ________________ Time of sampling: _____________________________

Buccal Code Number: ____

Number of Samples collected from this individual: _______________

Notes/Observations:

____

____

____

Supplementary material S5.10

SOP-10 Comparing occupational hygiene measurements with exposure estimates generated using the Advanced REACH Tool via the TREXMO model

Authors and Acknowledgements

Lead authors:

This document has been developed by Karen Galea (IOM, UK) and Susana Viegas (ESTeSL-IPL, Portugal).

Contributions were received from:

Radu-Corneliu Duca (LNS, Luxembourg), Katrien Poels (KU Leuven University, Belgium) and Sanni Uuksulainen (FIOH, Finland).

This document has been created for the HBM4EU project. HBM4EU has received funding from the European Union's Horizon 2020 research and innovation programme under grant agreement No 733032.

Scope

Many different approaches can be used for estimating occupational exposure to chemical substances. More recently, and probably primarily due to regulatory influence following the introduction of the REACH regulations, the use of predictive exposure models is becoming more frequent as it is not possible for the occupational hygiene community to collect a sufficient number of exposure measurements to generate estimates for all relevant exposure scenarios (Fransman, 2017; Landberg et al., 2018).

When applying exposure assessment modelling tools, users are required to select options from a number of possible input parameters. Hence, results obtained with the tools could be affected by factors such as the professional experience and judgment of the tool user and access to an appropriate level of information.

Several Tier 1 screening models such as ECETOC-TRA, MEASE, ART 1.5 and others are recommended for use under REACH (ECHA, 2016) and were evaluated under the E-TEAM project of which the results have been reported in several papers (Lamb et al, 2015, 2017, Tischer et al, 2017, van Tongeren et al, 2017). Lamb et al (2017) reported a between-user reliability exercise where exposure estimates ranged over several orders of magnitude for the same exposure situation by different users. It was also noted that the amount of contextual information provided in the situations could have potentially affected the level of variation between users. To explore this further a standardised proforma will be used in the HBM4EU occupational studies to collect contextual information about the work activities observed during the measurement campaign. For each visit, an exposure scenario will be generated.

At a later stage (and without knowledge of the results of the measurement campaigns), participants with differing knowledge about the workplace environments and activities will be given the generated workplace exposure scenarios and asked to use a selected REACH model, ART 1.5 via the

TREXMO (TRanslation of EXposure MOdels) tool with participants being asked to estimate inhalation exposure. TREXMO is a tool primarily developed to efficiently and reliably assess the wide variety of exposure situations using the available occupational exposure models. TREXMO integrates six commonly used occupational exposure models: ART v.1.5, Stoffenmanager® version 4.0, ECETOC TRA v.3, MEASE v.1.02.01, EMKG-EXPO-TOOL and EASE v.2.0, although we will only be using the outputs of ART 1.5 (the higher exposure assessment tool). Comparisons of the exposure estimates generated between the different types of users will be made, with these estimates also being compared with the actual exposure measurement results.

This Standard Operating Procedure (SOP) is partially based on earlier work undertaken by the IOM which has focused on the evaluation of exposure models (e.g. Lamb et al, 2015;2017; van Tongeren et al, 2017). This SOP is focused on: a. the collection of contextual information to inform the development of exposure scenarios to be used in the modelling exercises and b. the administration of the modelling exercise to participants. Details of how the collected data will be analysed is not provided in this SOP.

Contextual information to be collected during site visits

A standardised proforma will be used to gather relevant contextual information during the field survey measurement campaigns (Appendix 1). Information to be gathered will be from researchers first hand observations of the workplaces and activities taking place there and will include, for example, details of the risk management measures in place and used, operational conditions, materials generated, used etc. This proforma is to be completed on the same day that the air and other environmental samples are collected.

Generation of exposure scenarios for use in modelling exercise

The competed proformas will be returned to KG (karen.galea@iom-world.org) and SV (susana.viegas@ensp.unl.pt), who will use these to generate exposure scenarios to be used in the modelling exercise.

A standardised single A4 page format will be used for the generated exposure scenarios to minimise participant uncertainty from differences in layout of the descriptive information. An example of what these may look like is provided in Appendix 2 (this example being from the IOM E-TEAM project).

Model to be used

Participants will be asked to generate inhalation exposure estimates for the various scenarios using the higher tier REACH model, ART 1.5. This will be via the TREXMO tool, http://trexmo.chuv.ch/.

Who should complete the modelling exercise?

Each participating country where air samples will be collected as part of the biomonitoring campaigns, are invited to participate. In each country the following participants will be directly involved:

- The occupational hygienist / researcher who completed the contextual information template and collected the air and and other environmental samples.
- A member of the project team who did not visit the sites.
- An individual experienced in exposure assessment but who has no direct experience of the projects or sites where exposure to the chemicals being studied was assessed.

The modelling exercise

Overview

Participants will be asked to complete a short background questionnaire. They will then be provided electronically with a pack containing simple instructions for completing the exercise, instructions on how to use TREXMO (ART 1.5), the exposure scenarios and supporting worksheets. Details of how the assessments are to be returned will also be provided.

Background questionnaire

A short background questionnaire will be administered to collect key information on participants' experience in relation to the measurement campaign and also their use of modelling tools.

The participants will be requested to provide the following information:

- Organisation they work for.
- Years of experience in exposure assessment.
- The nature of their involvement in the measurement campaigns, e.g. if they personally collected the contextual information / air and other environmental samples at the sites; if they did not attend any site visits; had no involvement with the studies being developed.
- Previous experience with the use of the model.

How to use TREXMO

A simple guide to gaining access to TREXMO and how to use the ART 1.5 model for the purposes of the exercise will be provided. Guidance and screenshots detailing the required tool outputs will also be included.

Exposure situations

Depending on the number of exposure situations which are eventually generated, participants may receive these in batches or all at once.

For each exposure scenario, participants will be instructed to undertake an inhalation exposure assessment using ART 1.5, even where the scenario may be outwith the scope for the tool. For each exposure scenario they will be asked to complete a worksheet to record their results .

For each exposure scenario issued, participants will be required to document systematically the following contextual information on the worksheet:

- Previous experience of the given exposure scenario.

- Instances where they found choice or description of parameter types difficult, i.e. the level of uncertainty in their choice for example when selecting substance characteristics or risk management measures.
- The outputs derived by the tool.
- Their perception of the level of over/ under-estimation of the exposure estimate generated by the tool.

Participants will be asked to complete the given exposure scenario and return the completed worksheet within a specified period of time. A reminder will be issued in the event of non-receipt.

Data preparation

The exposure assessment outputs will be harvested from the returned worksheets and questionnaires and tabulated for analysis in Microsoft Excel spreadsheets.

References

ECHA Guidance Part D. European Chemical Agency, 2016. In: Guidance on Information Requirements and Chemical Safety Assessment, Chapter R.14, version 3.0, Helsinki, Finland.

Fransman W. (2017) How accurate and reliable are exposure models? Annals of Work Exposures and Health; 61(8): 907-910. https://doi.org/10.1093/annweh/wxx068

Landberg HE, Westberg H, Tinnerberg H. 2018.Evaluation of risk assessment approaches of occupational chemical exposures based on models in comparison with measurements. Safety Science, 109: 412–420

Lamb J, Galea KS, Miller BG, Spankie S, Van Tongeren M, Hazelwood G. 2015. Evaluation of Tier 1 Exposure Assessment Models under REACH (eteam) Project – Substudy report on between-user reliability exercise (BURE) and workshop. Dortmund. Avaliable from. https://www.baua.de/EN/Service/Publications/Report/F2303-D22.pdf?__blob=publicationFile&v=2

Lamb J, Galea KS, Miller BG, Hesse S, Van Tongeren M. 2017. Between-User Reliability of Tier 1 Exposure Assessment Tools Used Under REACH. Annals of Work Exposures and Health,61(8):939-53. https://doi.org/10.1093/annweh/wxx074

Tischer M, Lamb J, Hesse S, van Tongeren M. 2017. Evaluation of Tier One Exposure Assessment Models (ETEAM): Project Overview and Methods. Annals of Work Exposures and Health, 61 (8): 911–920. https://doi.org/10.1093/annweh/wxx066

Van Tongeren M, Lamb J, Cherrie JW, Maccalman L, Basinas I, Hesse S. 2017. Validation of lower tier exposure tools used for REACH: comparison of tools estimates with available exposure measurements. Annals of Work Exposures and Health. 61(8):921–938. https://doi.org/10.1093/annweh/wxx056

Appendix 1: Contextual information proforma

**Worker Information:**	
Date:	
Participant ID:	
Job Title:	
Shift Length (hours)	

**General working environment**	
Location	Indoor / Outdoor
Room temperature	°C
Approximate size of room where the participant works	m^3
Ventilation in room	Natural / Mechanical / Both
Air change rate	air changes per hour
Free text description of how well room is ventilated	
General impression of cleanliness and tidiness of workplace	Poor / Good / Excellent
Frequency of cleaning per day and how	

**General hygiene provision**	
Hand-washing facilities in immediate work location?	Yes / No
Do workers shower at end of the shift?	Yes / No
Separate eating/ drinking area?	Yes / No
Others:	

**Training**

Workers received previous training on the health risks associated with their work?	Yes / No
If yes, please specify	

Activities and tasks within these	
Description of activities taking place in this environment and numbers of workers involved (for each activity and in total)	
Description of tasks undertaken by workers and how they may be exposed:	
Process classification (continuous/batch) across operations and tasks	
Level of automation, mechanisation and manual interventions in process	
Any tools being used / manipulated and how this is done?	Description of tools used :<br><br>Please shortly describe the tools utilization for the different task:<br><br>Time spent on each task / activity during the day

Approximate working distance of worker from the exposure source	……………m<br><br>If different distances for different workers please specify
Any observed differences in working behavior between workers involved in same activity?	
Were the tasks /activities observed on the day typical of usual work activities?	Yes / No<br><br>If no, were samples/information collected during periods of lower / higher work rates?<br><br>How do these differ over the course of a week/year?

**Nature and sources of hazardous substance**	
Physical state of substance:	solid……………. Dustiness: ………………..<br><br>liquid……………. Vapour pressures (Pa) at 20°C: …………….....
Concentration of hazardous substances being assessed in preparation/product?	☐ <1%<br>☐ 1-5 %<br>☐ 5-25%<br>☐ >25%<br>☐ 100%
Sources of emission and subjective assessment of where emissions may be high	

**Operational conditions**	
Frequency and duration of exposure of workers conducting the	

tasks that can imply exposure (e.g. Task X: 120 min. twice a shift)	
Amount of substance handled:	Kg per shift
Use rate (include units):	
Process conditions that can be relevant (e.g. heated bath, high current applied)	
Process temperature	°C
Level of automation (e.g. manual)	

**Risk management measures (description and comment on each of these**	
Segregation – description	
Enclosure	
Local exhaust ventilation controls, description and comment on position, use, effectiveness	
Suppression techniques	
Control rooms description – time spent (minutes or hours)	
Others	

**PPE**

RPE usage by worker during activities, cleaning/storage regime	Type of RPE used: ……………………………………<br><br>Supplier APF: …………………<br><br>% of time/tasks being used:
Protective gloves	Type: ……………………………………<br><br>% of time/tasks being used:

Appendix 2: Example of exposure scenarios to be generated

Example exposure situation from E-TEAM project (Lamb et al, 2017).

Situation 15: Packing of Nickel Metal Powder

Please assess inhalation and dermal exposure to **nickel** in the situation described below.

When entering data into the tools during the exercise, please use the CAS number, molecular weight and vapour pressure value given in the table below.

1. General Description of Exposure Situation

This situation describes the packing of nickel powder in drums.

The operator removes excess powder (Product R) from a pre-weighed drum using a hand scoop and places the surplus material into a storage bin located at the packing station (Work Area R). If the containers are below the required weight, the operator uses the scoop to transfer powder back from the storage bin into the drum.

The operator then fixes a sealing cap onto an open aperture on the top of the drum.

The packing station is provided with local exhaust ventilation at the filling point. An air assisted filtering visor fitted with P3 filters is worn. All packing operators wear cotton overalls and safety boots. Gloves are not worn during scooping of powders.

The activity takes place at room temperature (20°C) in a small room with general ventilation.

The activity takes place for approximately 3 hours per 8-hour shift.

2. Product/ Substance Information

Product	Supplier	Substance Name	CAS Number	Molecular Weight/ gmol^{-1}	Vapour pressure at 20°C/ Pa	Concentration of Nickel in Product R (%)
Product R	Supplier R	Nickel	7440-02-0	59	1 (Negligible)	100

Supplementary material S5.11

SOP-11 Communication plan for the occupational studies

Authors and Acknowledgements

Lead authors:

This document has been developed by Susana Viegas from Escola Superior de Tecnologia da Saúde de Lisboa (ESTeSL), Instituto Politécnico de Lisboa, Portugal.

Contributions were received from: Karen Galea (IOM, UK), Bernice Schaddelee-Scholten (TNO, the Netherlands), Radia Bousoumah, Sophie Ndaw (INRS, France) and Tiina Santonen (FIOH, Finland).

This document has been created for the HBM4EU project. HBM4EU has received funding from the European Union's Horizon 2020 research and innovation programme under grant agreement No 733032.

General introduction

In the scope of HBM4EU project two further occupational studies will be developed.

The first is focused in diisocyanate exposure in the manufacturing and repair of large vehicles (non-booth spraying of e.g. boats/planes), the use of diisocyanate based hot-melt glues in different sectors, and construction sector, which includes different sources of diisocyanates exposure (floorings/screeds, insulation). Its main aims are to provide new data on the exposure to diisocyanates in specific sectors based on harmonized sampling protocols, test the usability of different biomarkers in the assessment of exposure to diisocyanates, and use the collected data to validate the PBPK model developed. The study will be conducted during 2020-2021 in five countries: Belgium, Finland, France, The Netherlands and United Kingdom.

The second study is dedicated to occupational exposures in the e-waste handling sector. The main aim is to contribute to awareness of potential hazards and stimulate good work practices that will lead to further improve protection of the worker's health from the risk of exposure to toxic components, including that of combined exposures. The study will include the assessment of exposure to several HBM4EU priority compounds, including metals (lead, inorganic mercury, cadmium, chromium), phthalates, and flame retardants. The study will be performed in Belgium, Finland, Germany, Hungary, Latvia, Luxembourg, The Netherlands, Poland, Portugal and United Kingdom.

This Communication Plan SOP is designed to ensure that all the relevant results of these studies are communicated to all study participants (companies, workers and equally to controls) and national and EU stakeholders. Dissemination of information to the scientific community is also covered. The results will be presented in different manner dependent of the type of participant (e.g. individual levels for workers and or aggregated levels to companies). Effective and consistent communication with participants and stakeholders will help ensure that the results obtained are accessible to support organizational learning and used for decision-making. This will happen at the companies and workers level but also at regulatory level. To sum up, it is CRUCIAL to consider that communication is very relevant and it is a way to

guarantee that the tools and data being developed in the scope of HBM4EU will be available to be used by risk assessors.

This SOP has been created with the premise in mind that every participating country is obliged to follow the HBM4EU documents and guidance concerning ethical aspects and also the national ethical committees.

Communication Plan

Considering the above a communication plan is proposed for both occupational studies (Table 1). This communication plan describes the different target audiences, the objectives/actions desired, the message content for each stakeholder/group, the method of choice to convey the message and, finally, the best moment in time for the message to be communicated. Additionally, it is important to consider that this is a living document and it will be reviewed and revised as necessary throughout the full duration of the occupational studies.

Table 1 – Current communication plan

Stakeholder/group	Objectives/actions desired	Data to be communicated	Delivery methods/venue	When
Study participants (workers and controls)*	Fulfill conditions set in informed consent procedure.<br>Inform the workers about their individual results to support behavioral change if needed	Individual results of exposure as per study information leaflet and consent form. In some countries they will only be provided to the study persons on request. Extra care will be given to the communication of unexpected results (procedure as specified in approved study protocol).	Depending on the country, for example via occupational physician of the company, or directly by the responsible researcher/physician of the national research group. Workers are given an opportunity to discuss on their results and on their meaning.	When the results are available for each company engaged in the project.
Participating companies**	Inform companies about the exposure results to support the risk assessment, definition of priorities and improve risk management measures.	Results of the occupational hygiene (air and wipe/settled dust) samples).<br>Results of the exposure biomarkers specified in information leaflet and informed consent form will be provided in an aggregated manner	Technical report be issued to the authorised person who consented to the company participating (unless otherwise specified)	When the results are available for each company engaged in the project.

Stakeholder/group	Objectives/actions desired	Data to be communicated	Delivery methods/venue	When
		guaranteeing that workers identities are not perceived; Recommendations for Risk Management Measures (RMM) if needed.		
HBM4EU partners	Provide study results and recommendations with respect to the outlined study aims and objectives. This intends to be a continuing process.	Exposure data obtained in each country will be combined with the other countries and statistical analysis will be performed	Deliverable report, presentations in consortium meetings and training school (lessons learned)	When the results are ready, by the due date of Deliverable report to HBM4EU.
HBM4EU partners and other interested partners	Inform about the methodology to be employed in the occupational exposure monitoring campaigns	Developed finalized SOPs	HBM4EU training school<br>Website<br>Publications<br>Linkedin	When SOPs have been finalized.
Scientific and professional community	Inform about the most suitable biomarkers for each substance, exposure levels, variables that influence exposure, occupational settings with higher exposure, Risk Management Measures (RMM) with higher efficacy	Exposure data obtained in each country will be combined with the other countries and statistically analysis will be performed.	Scientific publications in peer reviewed journals; presentations at conferences and seminars (at national and international level) and online webinars	Begins already when the methodology has been established, and continues when the results are ready.
HBM4EU stakeholders (ECHA, EU-OSHA and national contact points via their focal contact point network, DG Santé, DG Employment) and other interested parties (industry associations, workers unions, national authorities,<br>ISES-Europe and European occupational hygiene associations)	Inform about more suitable biomarkers, occupational settings with higher exposure, evaluate the impact of the regulatory actions already in place, the support of new regulatory actions if needed.	Aggregated exposure data	Articles in HBM4EU newsletter<br>Policy brief<br>HBM4EU webpages<br>Presentations in HBM4EU stakeholder forum. Deliverable report<br>Webinar/seminar on the results targeted to key stakeholders. Slides from the Webinar published in web.<br>Information is shared also using social media channels.	Dates to be defined. Communication begins already when the studies start and continues throughout the study.
Targeted communication to ECHA Committees if relevant (e.g. RAC).	Evaluate the impact of the regulatory actions already in place, the support of new regulatory actions if needed.	Aggregated exposure data	Presentation in ECHA	When the results and deliverable report are ready

Stakeholder/group	Objectives/actions desired	Data to be communicated	Delivery methods/venue	When
National industry stakeholders, workers unions (optional, depending on country)	Inform about the exposures in different occupations, recommendations for the assessment and management of exposure.	Aggregated exposure data	Press release in national language, description of the study published in the website of the participating institutes. Possibly short articles in national journals targeted to relevant industry fields. Use of social media to distribute information also nationally.	This is the responsibility of each participating institute (dates do be defined)

* Communication starts in the first contact companies/workers and information about why they should participate in the study and what are the hazards related to their exposures should be provided since the beginning of the study. ** Preferably done before any reporting to HBM4EU, stakeholders and scientific community.
