# Supplementary material for: HBM4EU Occupational Biomonitoring Study on e-Waste—Study Protocol
Source: Int J Environ Res Public Health. 2021 Dec 9;18(24):12987. doi: 10.3390/ijerph182412987 (PMC8701897; doi:10.3390/ijerph182412987)
Supplement: Supplementary file 1 [file ijerph-18-12987-s001.zip › Supplementary material S6.pdf]

Study Protocol

HBM4EU Occupational Biomonitoring Study on e-Waste—Study Protocol

HBM4EU E-Waste Study Team: The HBM4EU e-waste study team consists of Kukka Aimonen [11], Lasma Akulova [17], Radia Bousoumah [10], Argelia Castaño [13], Adam Clarke [7], Adrian Covaci [14], Maurice van Dael [1], Marta Esteban López [13], Thomas Göeno, Martien Graumans [1], Ogier Hanser [10], Beata Janasik [16], Kate Jones [7], Laura Komarovska [17], Sirpa Laitinen [11], Carla Martins [12], Linda Matisane [17], Inese Martinsone [17], Rodrigo Moreira [8], Darren Musgrove [7], An van Nieuwenhuyse [2,4], Ana Nogueira [19], Linda Paegle [17], Hermínia Pinhal [19], Tiina Rantio [11], Edna Ribeiro [18], Anita Seile [17], Marina Silva [18], Sílvia Reis Santos [19], Marjo Vänskä [11], Riitta Velin [11], Célia Ventura [8,9] and Wojciech Wasowicz [16]

[1] Radboud Institute for Health Sciences, Radboudumc, Nijmegen, The Netherlands
[2] Department of Health Protection, Laboratoire national de santé (LNS), Dudelange, Luxembourg
[3] Institute of Occupational Medicine (IOM), Edinburgh, EH14 4AP, UK
[4] Centre for Environment and Health, Department of Public Health and Primary Care, KU Leuven (University of Leuven), Kapucijnenvoer 35, 3000 Leuven, Belgium
[5] IDEWE, External service for prevention and protection at work, Heverlee, Belgium
[6] Department of Public Health, University of Copenhagen, Copenhagen, Denmark
[7] Health & Safety Executive, Buxton, SK17 9JN, UK
[8] INSA, National Institute of Health Dr. Ricardo Jorge, Department of Human Genetics, Lisbon, Portugal
[9] Centre for Toxicogenomics and Human Health, NOVA Medical School, Universidade NOVA de Lisboa, Lisbon, Portugal
[10] INRS, French National Research and Safety Institute, Vandœuvre-lès-Nancy, France
[11] Finnish Institute of Occupational Health, Helsinki, Finland
[12] NOVA National School of Public Health, Public Health Research Centre, Universidade NOVA de Lisboa, 1600–560 Lisbon, Portugal; Comprehensive Health Research Center (CHRC), Portugal.
[13] Instituto de Salud Carlos III, Madrid, Spain
[14] Toxicological Center, University of Antwerp, 2610 Wilrijk, Belgium
[15] Institute and Outpatient Clinic of Occupational, Social and Environmental Medicine, Friedrich-Alexander-Universität Erlangen-Nürnberg, Germany
[16] Nofer Institute of Occupational Medicine, Lodz, Poland
[17] Rīga Stradiņš University, Dzirciema street 16, Rīga, LV-1007, Latvia
[18] Health & Technology Research Center, ESTeSL- Escola Superior de Tecnologia da Saúde de Lisboa, Instituto Politécnico de Lisboa. Lisbon, Portugal
[19] INSA, National Institute of Health Dr. Ricardo Jorge, Department of Environmental Health, Lisbon, Portugal
